# ReCAST: Single-Cell-Informed Tumour Microenvironment Profiling for Translational Prognostic Assessment in Esophageal Cancer

**DOI:** 10.3390/biomedicines14102298

**Published:** 2026-10-09

**Authors:** Nahlah Makki Almansour

**Affiliations:** Department of Biology, College of Science, University of Hafr Al Batin, Hafr Al Batin 31991, Saudi Arabia; nahlama@uhb.edu.sa

**Keywords:** precision oncology, cell-state deconvolution, single-cell reference, tumour microenvironment, prognostic modelling, external validation, esophageal cancer, biomarker evaluation

## Abstract

**Background/Objectives:** Single-cell transcriptomics provides detailed maps of the tumour microenvironment, but translating these maps to large clinical cohorts requires methods that can recover biologically meaningful cell states from routinely available bulk transcriptomes and establish whether these states provide information beyond standard clinical factors. The objective of this study was to develop ReCAST, a donor-aware method that estimates tumour microenvironment cell states from bulk tumour transcriptomes, and to test whether these estimates improved survival prediction in esophageal cancer beyond routine clinical variables. **Methods:** ReCAST constructed outcome-independent cell-state anchors from single-cell transcriptomes by first aggregating expression within donors and then across donors, followed by robust non-negative projection of bulk tumour profiles. The framework was evaluated using synthetic mixtures, 720 genuine-cell pseudo-bulk mixtures from 60 held-out donors, cross-study mixtures from an independent single-cell cohort, an independent adenocarcinoma single-cell cohort, and independently measured tumour compositions in 182 TCGA esophageal cancers. Biological validity was examined against Human Protein Atlas cell-type annotations. ReCAST-derived tumour microenvironment features were subsequently tested for incremental prognostic value beyond age, sex, histology, and stage using repeated cross-validation, and the clinical prediction models were evaluated by locked external validation in an independent cohort of 60 patients with squamous-cell carcinoma. **Results:** ReCAST recovered 13 biologically coherent cell states whose markers showed strong enrichment for corresponding independent cell-type annotations (fold enrichment 5.1–29.8; FDR < 10^−15^). Cell-state profiles were reproducible across held-out donors and independent studies and donor-aware reference construction improved cross-study transfer. ReCAST outperformed BisqueRNA and unbalanced optimal transport on held-out pseudo-bulk mixtures and, because it worked on within-sample ranks, extended cell-state profiling to data without raw counts, such as variance-stabilised, microarray or summarised reference data; where raw counts were available, count-based methods (MuSiC, DWLS, BayesPrism and a CIBERSORT-type ν-support-vector regression) achieved lower errors and were the preferred complement. In clinical tumours, inferred immune composition tracked independently measured lymphocyte infiltration and leukocyte fraction. Routine clinical variables remained the strongest predictors of survival and were transferred to the independent squamous-cell carcinoma cohort (Uno C-index 0.61); adding ReCAST-derived features produced no incremental improvement (ΔUno C-index −0.033, 95% CI −0.089 to 0.023), a result that was consistent within each histology and with an adenocarcinoma-matched reference. **Conclusions:** ReCAST provides reproducible cell-state profiles across donors, studies and histologies and extends tumour microenvironment profiling to expression data without raw counts. In esophageal cancer, routine clinical variables already capture the prognostic information carried by cell-state composition, which positions cell-state profiling as a tool for biological characterisation rather than risk prediction. Establishing measurement validity and clinical utility separately provides a robust route for evaluating molecular biomarkers.

## 1. Introduction

Esophageal cancer remains one of the most lethal solid malignancies, with a five-year survival of below one in five in most registries and a rising incidence of adenocarcinoma in Western populations [1,2]. Squamous-cell carcinoma and adenocarcinoma differ in aetiology, molecular features, and treatment response, yet routine prognostication still rests largely on the anatomical stage, and patients within the same stage group experience markedly different outcomes [3,4,5]. Precision oncology aims to close this gap by reading the biology of each tumour directly from molecular data, so that the choice of treatment follows a molecular diagnosis rather than anatomical extent alone [6].

Much of that biology is cellular. A tumour is an ecosystem in which malignant cells coexist with lymphoid, myeloid, and stromal populations, and the balance among these compartments shapes invasion, immune evasion, and the response to therapy [7,8,9]. Single-cell transcriptomics has catalogued these states in esophageal cancer at a high resolution [10,11], but the single-cell profiling of every patient is neither routine nor affordable, whereas bulk transcriptomes exist for thousands of tumours. Each bulk profile, however, averages expression over malignant, immune and stromal cells, so it has to be interpreted against the molecular heterogeneity that multi-omic analyses have described across tumour types [12]. Reference-based deconvolution bridges the two: a single-cell atlas defines the expression of each cell state, and the composition of a bulk tumour is inferred as a mixture of those states [13,14,15,16]. Accurate estimates of tumour, immune, and stromal fractions support tumour-purity correction, immune-contexture profiling, patient stratification for immunotherapy research, and the interpretation of bulk biomarkers in their cellular context.

Two questions determine whether such estimates are useful, and they are distinct. The first concerns measurement: does the method recover cell-state composition accurately, across donors, laboratories, and expression platforms? Estimates depend heavily on how the reference is constructed and on the robustness of the estimator to platform differences and to expression that the reference does not represent [16,17,18]. The second concerns clinical value: once composition is measured well, does it improve the prediction of patient outcomes beyond the clinical variables already available? The two are frequently conflated. Immune features derived from bulk transcriptomes have already been assembled into prognostic models, for example, an immune prognostic model for glioblastoma built from ssGSEA enrichment scores [19], but whether such features add information to the clinical variables already at hand is a different question from whether they describe the tumour microenvironment accurately. A biologically faithful cell-state profile need not carry prognostic information that stage, histology, and age do not already capture, and prognostic claims made in small cohorts are prone to optimism unless the incremental value is tested directly against established clinical predictors with appropriate internal and external validation [20,21,22,23,24,25,26].

The objective of this study was to develop a reference-based method that estimates cell-state composition consistently across donors, studies and expression platforms, and to test whether the resulting features improve survival prediction in esophageal cancer beyond routine clinical variables. Here, we present ReCAST, a donor-aware, reliability-weighted cell-state projection framework, and evaluate it along both axes. ReCAST constructs outcome-independent state anchors by summarising single-cell data within each donor before combining donors, weighting genes by external protein evidence from the Human Protein Atlas [27,28], projecting bulk tumours by Huber-reweighted non-negative least squares [29,30], and reporting a normalised residual that flags expressions that the reference does not explain. We establish its technical accuracy on synthetic, donor-held-out, and cross-study pseudo-bulk mixtures built from genuine single-cell counts; compare it with complete implementations of MuSiC, BisqueRNA, and DWLS [15,16,31] and BayesPrism [32] and the ν-support-vector regression core of CIBERSORT [13]; quantify the contribution of each design component and the number of reference donors the construction needs; validate tumour, lymphocyte, and leukocyte estimates against independently measured compositions in the Cancer Genome Atlas [33,34]; test whether anchors built from squamous-cell carcinoma transfer to adenocarcinoma, using an independent adenocarcinoma single-cell cohort [35]; and interpret the recovered states against independent single-cell annotations [36]. We then ask the translational question explicitly, testing whether ReCAST-derived features add prognostic information to a clinical model in 182 TCGA esophageal cancers under repeated nested cross-validation and evaluating the clinical models in an independent, non-overlapping external cohort with discrimination, calibration, and decision-curve analyses. The study is therefore built as a sequence, in which the measurement is validated first and its clinical utility is tested second, rather than as the development of a new prognostic model.

## 2. Materials and Methods

### 2.1. Study Design and Data Sources

**Figure 1:**
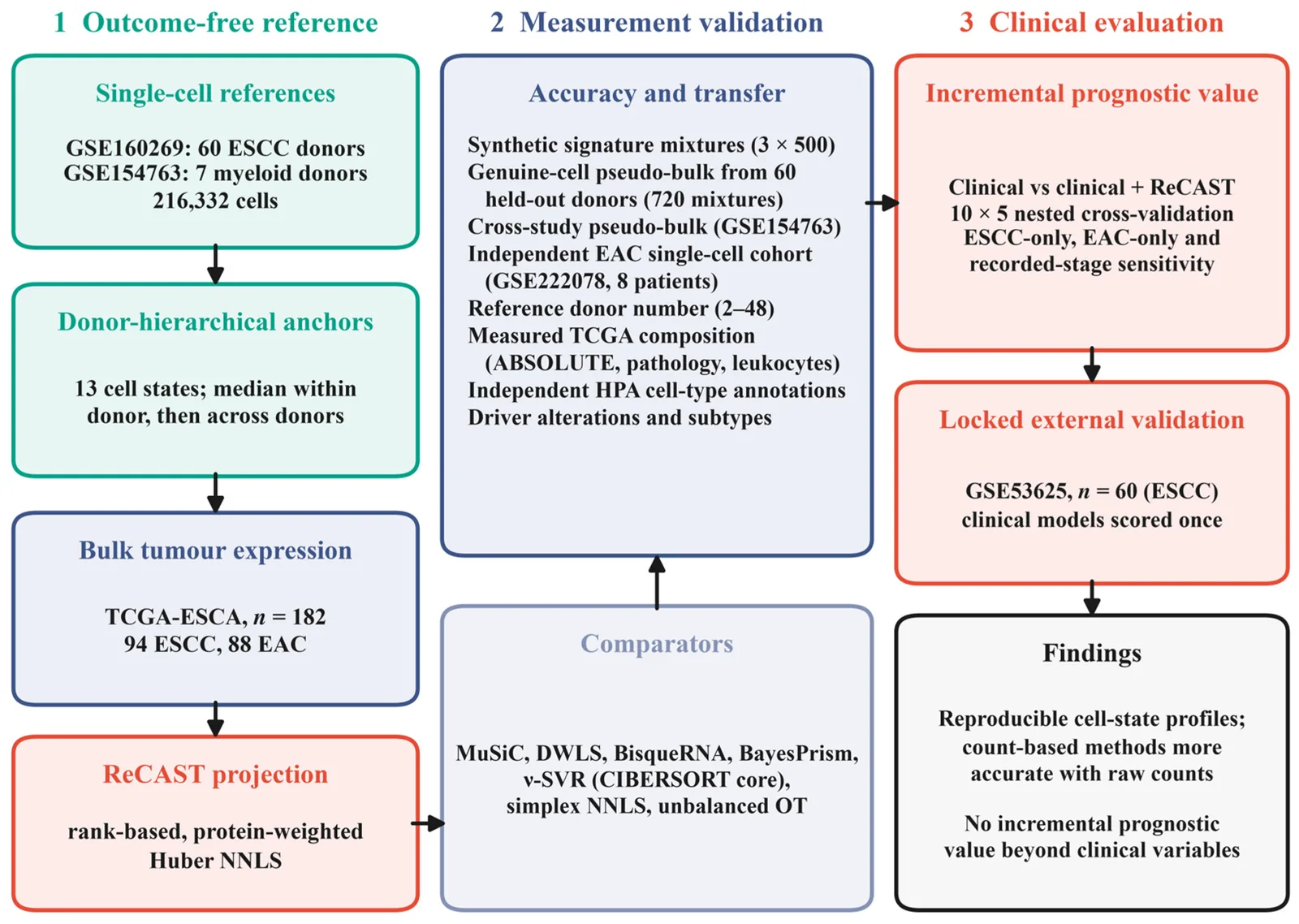
Study design. An outcome-free single-cell reference is summarised into donor-hierarchical cell-state anchors, and bulk tumour expression is projected onto them. The representation is validated on known mixtures, on genuine-cell pseudo-bulk from held-out donors, from an independent study and from an independent adenocarcinoma cohort, and against measured tumour composition, alongside seven comparators. Its features are then tested for incremental prognostic values against a clinical model, with histology- and stage-restricted sensitivity analyses, and the clinical models are carried to a locked external cohort.

This retrospective computational study had two linked components: the technical and biological validation of cell-state estimation, and an independent assessment of the incremental prognostic value of the resulting features. The overall design is shown in Figure 1. The survival analysis followed a version-controlled protocol fixed before the confirmatory benchmark (primary endpoint, feature panels, candidate models, split manifest, and external-validation policy). Analyses added during the preparation of this article are identified as such in Section 2.6, Section 2.7, Section 2.8, Section 2.9 and Section 2.10: cell-level donor-held-out and cross-study pseudo-bulk validation, comparisons with MuSiC, BisqueRNA and DWLS, controlled component analysis with shuffled-weight controls, validation against measured tumour composition, biological interpretation against independent annotations, characterisation of the unknown-expression score, parameter-sensitivity analysis, and dependence-aware uncertainty for repeated cross-validation. Analyses added during the peer review are described in the same sections: BayesPrism and ν-support-vector regression comparators, an independent adenocarcinoma single-cell reference, a reference donor-number simulation, T-cell compartment aggregation, associations with driver alterations, time-truncated discrimination, and histology-restricted and complete-case sensitivity analyses. These analyses used fixed seeds, did not modify the locked estimator or its parameters, and were not tuned on any evaluation set. The study is reported in line with TRIPOD + AI [25].

Bulk RNA-sequencing counts, variance-stabilised expression [37], clinical annotations, and marker–paper composition annotations for the TCGA esophageal cancer cohort (TCGA-ESCA) were obtained from the Genomic Data Commons [33,38]. Single-cell references were GSE160269, which provides raw unique-molecular-identifier (UMI) counts for CD45-positive and CD45-negative cells from esophageal squamous-cell carcinoma and adjacent tissue [10] and the esophageal subset of GSE154763, a pan-cancer atlas of tumour-infiltrating myeloid cells [11]. An independent adenocarcinoma single-cell cohort, GSE222078, provided counts for 52,387 cells from the tumours of eight patients with EAC, four treatment-naive and four after neoadjuvant chemotherapy, and from two adjacent normal samples [35]. Somatic mutation calls for TCGA-ESCA (TCGA PanCancer Atlas MC3 [39]) were obtained through cBioPortal [40]. Gene-level protein-evidence annotations and single-cell-type specificity annotations were taken from the Human Protein Atlas [27,28,36]. External survival validation used the microarray series GSE53624 and GSE53625 [41,42]. All data were public and de-identified.

### 2.2. Cohorts and Endpoint

One primary tumour per TCGA patient was retained for squamous-cell carcinoma (ESCC) and adenocarcinoma (EAC), giving 182 patients with 77 deaths (94 ESCC, 88 EAC). The endpoint was overall survival; one non-positive survival time was set to one day. The stage was taken from the TCGA marker–paper annotation and, where this was missing, from the GDC pathological and then clinical AJCC stages. Stage could not be assigned in 11 patients (9 EAC, 2 ESCC): none of these fields was recorded for 10 of them, six of whom came from a single tissue-source site, and one was recorded as stage unknown because the M category had not been assessed. These patients were kept with an explicit unknown-stage indicator rather than excluded, and a complete-case sensitivity analysis was reported (Section 2.10). Follow-up was summarised by the reverse Kaplan–Meier method [43]. An overlap audit showed that GSE53625 was a SuperSeries containing all 119 patients of GSE53624, so the external test set was restricted to the 60 GSE53625 patients absent from GSE53624 (33 deaths), all with ESCC.

### 2.3. Single-Cell Reference and Donor-Hierarchical Anchors

The reference comprised 216,332 cells from 67 donors (208,659 cells from GSE160269 and 7673 myeloid cells from GSE154763). Cell-state labels were the major-lineage annotations distributed with each study; GSE154763 myeloid subclusters were harmonised to dendritic cells (DCs), mast cells, and monocytes/macrophages (Mono/Macro). For every combination of donor *d*, sample *s*, and state *k*, UMI counts were summed over cells and converted to log-normalised expression, *p*_g_ = log(1 + 10^4^ *c*_g_/∑_h_ *c*_h_), where *c*_g_ is the summed count of gene *g*; for GSE154763, the provided normalised values were averaged. This yielded 965 donor–sample–state profiles over 13,614 genes shared by both studies.

Anchors were then formed hierarchically. Profiles were first summarised within donor by the gene-wise median across that donor’s samples, giving one profile per donor and state, and state anchors were the gene-wise median across donors:

*a*_kg_ = median*_d_* _∈ *D*(*k*)_ [median*_s_* _∈ *S*(*d*,*k*)_ *p*_dskg_], (1)

where *D*(*k*) is the set of donors contributing state *k* and *S*(*d*,*k*) the samples of donor *d* containing that state. Collapsing within each donor before combining donors gives each donor one vote per state, so that donors sampled more deeply, or contributing several tissue samples or fine subclusters, cannot dominate an anchor. States represented by at least two donors were modelled, giving 13 states: malignant cells, fibroblasts, endothelial cells, pericytes, B cells, plasma cells, conventional CD4 T cells (CD4Tconv), exhausted CD8 T cells (CD8Tex), regulatory T cells (Treg), proliferating T cells (Tprolif), DC, mast cells, and Mono/Macro. No outcome information entered reference construction.

### 2.4. ReCAST Projection

**Figure 2:**
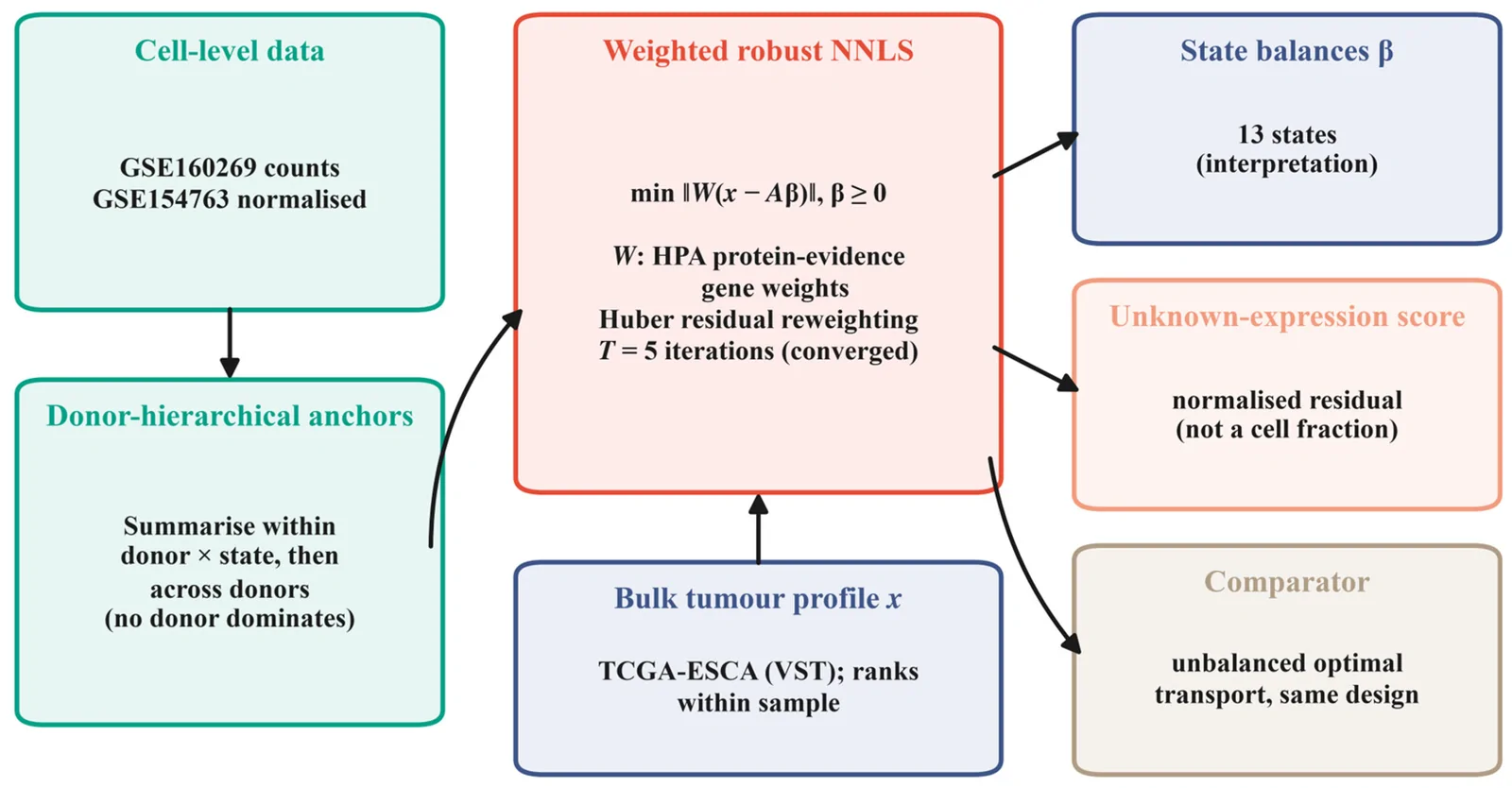
The ReCAST projection. Cell-level data are summarised within donor and state and then across donors, so that no donor dominates an anchor; bulk profiles are projected by protein-evidence-weighted robust non-negative least squares with Huber residual reweighting, returning state balances and a normalised unknown-expression score. Entropic unbalanced optimal transport is evaluated as a comparator on the same design.

**Genes and rank normalisation.** Let *G* be the genes shared by the reference and the bulk data set. Each anchor was converted to within-profile ranks, *r*_kg_ = rank_g∈G_(*a*_kg_)/|*G*|. Ranks make the representation invariant to monotone transformations of expression, so the same anchors apply to count-derived, variance-stabilised, or microarray data without platform-specific rescaling. The projection is summarised in Figure 2.

**Protein-evidence gene weights.** Each gene received a reliability value *ρ*_g_ from its Human Protein Atlas existence evidence (evidence at protein level 1.15; transcript level 1.00; predicted 0.75; uncertain 0.60; unannotated 1.00), bounded to [0.25, 1.25]. The gene weight was *w*_g_ = *ρ*_g_^λ^ with λ = 0.25. These annotations are external to both the reference and the target data.

**Marker selection.** For each state, the weighted specificity contrast was

*c*_kg_ = *w*_g_ (*r*_kg_ − median_j≠k_ *r*_jg_), (2)

and the *m* = 40 genes with the largest positive contrast were selected; the union over states formed the marker set *M* (326 genes for TCGA-ESCA).

**Design matrix and bulk representation.** The design matrix had entries *A*_gk_ = *w*_g_*r*_kg_/‖*w*∘*r*_·k_‖_2_, g ∈ *M*, so that each state column has a unit norm. For a bulk sample, marker expression was ranked within the sample, weighted, and scaled to the unit norm, ${\overset{\;\;\;\;\sim }{x}}_{g}$ = *w*_g_ rank(*x*_g_)/‖*w*∘rank(*x*)‖_2_.

**Robust non-negative estimation.** State coefficients were estimated by iteratively reweighted non-negative least squares:

(3) $$\beta^{\left(t\right)}={\mathrm{argmin}}_{\beta \;\geq \;0}\;\sum_{g\in M}\;{v_{g}}^{\left(t-1\right)}\;{\left({\overset{\;\;\;\;\sim }{x}}_{g}-\sum_{k}\;A_{\mathrm{gk}}\;\beta_{k}\right)}^{2},$$

starting from *v*_g_^(0)^ = 1. Each weighted problem was solved exactly with the Lawson–Hanson active-set algorithm [29]. After each iteration, the residuals *e*_g_ = ${\overset{\;\;\;\;\sim }{x}}_{g}$ − (*A*β^(t)^)_g_ were standardised by a robust scale, σ = 1.4826·MAD(*e*), and Huber weights were updated as *v*_g_ = 1 if |*e*_g_|/σ ≤ δ and *v*_g_ = δσ/|*e*_g_| otherwise, with δ = 1.5 [30]. Genes whose bulk expression departs strongly from every anchor combination are thereby down-weighted rather than allowed to distort the fit. The locked implementation performs *T* = 5 reweighting iterations; convergence was verified by the change in coefficients between successive iterations (Section 3.1).

**Outputs.** The estimated composition was *π*_k_ = β_k_/∑_j_β_j_. Two fit diagnostics were retained: the unknown-expression score

(4) $$U=\|\overset{\;\;\;\;\sim }{x}-A\beta {\|}_{2}/\|\overset{\;\;\;\;\sim }{x}{\|}_{2}$$

and the reconstruction cosine between $\overset{\;\;\;\;\sim }{x}$ and *A*β. *U* summarises how much of the sample’s marker expression the anchors cannot reproduce; it is not an estimate of an unknown cell fraction.

**Parameter selection.** The values *m* = 40, λ = 0.25, δ = 1.5, *T* = 5, and the two-donor threshold were fixed in the locked configuration before any validation or outcome analysis and were not tuned on evaluation data. Their influence was examined afterwards in a sensitivity analysis (Section 2.6).

### 2.5. Established Deconvolution Methods

ReCAST was compared with three complete, widely used single-cell-reference deconvolution implementations, each run with its recommended inputs: raw single-cell UMI counts with cell-state and donor labels, and raw bulk counts. MuSiC (version 1.0.0) weights informative genes by cross-donor consistency [15]; BisqueRNA (version 1.0.5) was run in reference-based mode, which learned gene-specific bulk transformations [16]; and DWLS (version 0.1.0) built a signature from differential expression and solved a dampened weighted least-squares problem [31]. DWLS’s differential-expression step was run with its own test and signature-selection procedure through Seurat version 5.5.1 [44]. The single-cell reference supplied to these methods retained up to 25 cells per sample and states from the same training donors used by ReCAST; the DWLS differential-expression step was run on at most 10 cells per donor and state for tractability. Two estimators applied to the ReCAST design itself were also included: simplex-constrained least squares and entropic unbalanced optimal transport [45,46,47]. Two further count-based comparators were added during peer review. BayesPrism (version 2.2.3) [32] was run on raw counts with malignant cells declared as the tumour component and with its recommended removal of ribosomal, mitochondrial and sex-chromosome genes. CIBERSORTx [14] (https://cibersortx.stanford.edu/, accessed on 13 June 2026) was distributed as a licensed web and container service, so its published ν-support-vector regression (ν-SVR) core [13] was implemented directly: a signature of the 100 genes per state with the largest fold change over the next-highest state was built from the reference cells in counts per million, each sample was fitted by linear ν-SVR with ν of 0.25, 0.5 or 0.75 chosen by root-mean-square error, and negative coefficients were set to zero. Both methods used the same reference cells and bulk inputs as MuSiC, BisqueRNA and DWLS.

### 2.6. Validation of Cell-State Estimation

**Signature mixtures.** As a first technical check, 500 synthetic mixtures per scenario were generated from the anchor signatures under clean, platform-shift (random power transformation with noise), and unknown-component (up to 40% out-of-reference signal) conditions, and state-composition mean absolute error (MAE) was compared between ReCAST and unbalanced optimal transport.

**Donor-held-out pseudo-bulk.** Mixtures were then built by summing genuine cell counts, so that library size, sparsity, and cell-to-cell heterogeneity were those of real data. The 60 GSE160269 donors were divided into five donor-level folds. In each fold, anchors and the references for MuSiC, BisqueRNA, and DWLS were constructed from the training donors only, and 12 mixtures per held-out donor were generated from that donor’s tumour cells: state fractions were drawn from a symmetric Dirichlet distribution over the states the donor contributed (at least 10 cells each), and 2000 cells were sampled accordingly. The truth was the realised cell fraction of each state.

**Cross-study pseudo-bulk.** To test the transfer between independent studies, anchors built from all GSE160269 donors were applied to mixtures of GSE154763 tumour myeloid cells (seven donors, 40 mixtures each), after harmonising both studies to the DC, mast-cell, and Mono/Macro labels; for this analysis, GSE154763 counts were recovered exactly from the distributed normalised values (median absolute deviation from integers 5 × 10^−5^). The reverse direction applied myeloid anchors built from GSE154763 to GSE160269 myeloid mixtures. Accuracy was evaluated on the relative composition of the harmonised states, with mass assigned to other states reported separately. A matched within-study control used GSE160269 myeloid mixtures from held-out donors. No parameter of any method was adjusted on these test sets.

**Independent adenocarcinoma reference.** The distributed counts carried small non-integer corrections and were rounded to integers. GSE222078 cells with at least 200 detected genes and less than 25% mitochondrial counts were log-normalised; 2000 highly variable genes were reduced to 30 principal components and partitioned into 40 clusters by k-means. Each cluster was assigned the lineage with the highest mean standardised marker score (epithelial, T/NK, B, plasma, monocyte/macrophage, dendritic, mast, endothelial, fibroblast or pericyte) when that score exceeded 0.5 and led the next lineage by at least 0.25; other clusters were left unassigned. Epithelial cells from tumour samples were labelled malignant, and T cells were divided into CD8Tex, CD4Tconv, Treg and Tprolif states by cell-level marker scores, giving 28,187 tumour cells in the 13 modelled states. Three questions were asked. First, each EAC patient’s state profiles were correlated with the locked anchors and compared with held-out ESCC donors. Second, 240 pseudo-bulk mixtures (30 per patient, 2000 cells each) were projected onto the locked ESCC-derived anchors, onto anchors built from the other seven EAC patients, and onto combined ESCC and EAC anchors; MuSiC, BisqueRNA, DWLS, BayesPrism and the ν-SVR core were run on the same mixtures with the GSE160269 cell reference used for TCGA. Accuracy was summarised over four lineages (malignant, lymphoid, myeloid and stromal) because the EAC states were assigned here by marker scoring rather than by the original authors. Third, the TCGA EAC tumours were re-projected onto a histology-matched reference, in which the malignant anchor came from the eight EAC patients and the other anchors from pooled ESCC and EAC donors, and agreement with measured composition, the unknown-expression score, the histology contrasts and the prognostic contrast were recomputed.

**Measured tumour composition.** In the 182 TCGA-ESCA tumours, estimated malignant fractions were compared with ABSOLUTE tumour purity derived from copy-number data [34,48] and with pathology estimates of percent tumour cells and tumour nuclei; estimated lymphoid fractions with pathology percent lymphocyte infiltration; and estimated immune (lymphoid plus myeloid) fractions with the estimated leukocyte fraction reported by the TCGA Research Network [33]. Agreement was summarised by Spearman correlation with 2000-sample bootstrap confidence intervals and differences between estimators by paired bootstrap on identical resamples. MuSiC, BisqueRNA, and DWLS were applied to the raw TCGA counts with a GSE160269 cell reference. Agreement was also estimated separately within ESCC and within EAC.

**Metrics and inference.** The primary accuracy metric was per-mixture MAE between estimated and true state fractions; the root-mean-square error and the within-mixture Pearson correlation were secondary. Because mixtures from the same donor are correlated, 95% confidence intervals and paired differences between methods were obtained by resampling donors (2000 replicates). To separate misassignment among T-cell states from the loss of T-cell signals, the four T-cell states (CD4Tconv, CD8Tex, Treg and Tprolif) and the six lymphoid states were also summed and compared with the true totals.

**Parameter sensitivity and convergence.** On donor-held-out folds, the marker number (*m* = 20, 40, 80), Huber constant (δ = 1.0, 1.345, 1.5, 2.0, and no reweighting), weight exponent (λ = 0, 0.25, 0.5, 1), and iteration number (*T* = 1–20) were varied one at a time around the locked values, and the relative change in β between successive iterations was recorded.

### 2.7. Component Analysis

The contribution of each design element was tested by removing it while holding everything else fixed, on identical mixtures: (i) no donor balancing, replacing donor-hierarchical anchors with cell-count-weighted pooled profiles; (ii) no donor collapse, taking the median over all sample-level profiles; (iii) no protein weighting (λ = 0); (iv) no robust reweighting (*T* = 1, ordinary NNLS); and (v) all three principal components removed. To test whether the protein annotations carried gene-specific information rather than acting as a generic perturbation, 20 shuffled-weight controls permuted the reliability values across genes before marker selection and fitting.

**Reference donor number.** To find how many reference donors the donor-hierarchical construction needed, anchors were rebuilt in each donor-held-out fold from random subsets of 2, 3, 4, 6, 8, 12, 16, 24 and 36 of the 48 training donors (10 draws per size) and from all training donors, and the same held-out mixtures were projected. Three versions were compared on identical subsets: locked ReCAST; a simplified ReCAST-core that keeps the donor-hierarchical anchors but omits protein weighting and robust reweighting (λ = 0, T = 1); and the locked estimator with cell-pooled anchors.

### 2.8. Biological Interpretation of Recovered States

For each state, the 40 anchor markers selected in the locked fit were compared with Human Protein Atlas single-cell-type annotations [36], which were independent of the protein-existence evidence used for weighting. A marker was counted as annotated to the matching cell type when that type appeared among the gene’s enhanced, enriched, or group-enriched single-cell types (for example, B cells for the B state; esophageal basal, suprabasal, and apical cells and keratinocytes for malignant ESCC cells). Enrichment against all annotated genes in the reference was tested with the hypergeometric distribution, and all state-by-cell-type tests were corrected by the Benjamini–Hochberg procedure [49]. Consistency across donors was measured by the Spearman correlation between each held-out donor’s state profile and the anchors built without that donor, and by whether the matching anchor was the best-correlated one. Consistency across studies was measured by the overlap of myeloid markers selected independently in GSE160269 and GSE154763. Distinguishability was assessed from pure single-state pseudo-bulk samples (500 cells) of held-out donors, recording the fraction of each sample assigned to every state, together with anchor-to-anchor rank correlations. State stability was additionally assessed by 500 donor-bootstrap replicates of marker selection, calling a state–gene pair stable at a selection frequency of at least 0.70. Differences in the estimated composition between EAC and ESCC were tested per state by the Mann–Whitney test with Benjamini–Hochberg correction; reproducibility was assessed across estimators and by requiring significance in both halves of 500 random half-splits of the cohort.

**Driver alterations and molecular subtypes.** As an exploratory and descriptive analysis, state fractions were related to non-synonymous somatic mutations in canonical esophageal cancer drivers mutated in at least 5% of the 180 patients with sequencing data (14 genes), to ERBB2 amplification and CDKN2A silencing from the marker-paper annotation, and to the TCGA molecular subtypes. The rank of each state fraction was regressed on each alteration with histology as a covariate, with Benjamini–Hochberg correction across all 208 tests; the EAC–ESCC difference in each state was re-estimated after adjustment for all 16 alterations together; and subtypes were compared within histology by the Kruskal–Wallis test. These analyses describe co-occurrence and do not test causal or functional relationships.

### 2.9. Characterisation of the Unknown-Expression Score

Anchors were built from 48 GSE160269 donors and mixtures from the remaining 12. The unknown-expression score was compared with unperturbed mixtures under three kinds of perturbation: omission of a state from the anchors while that state was present in mixtures at fractions of 0 to 0.5 (malignant cells, fibroblasts, Mono/Macro, CD8Tex, B cells, endothelial cells, plasma cells), with the complete anchors as control; technical noise, by binomial down-sampling of library depth (10^6^ to 10^4^ UMIs) and gene-wise multiplicative gamma noise (coefficient of variation 0.1–1.0); and platform difference, by gene-specific log-normal bias (σ = 0.25–1.5) and by applying GSE160269 anchors to GSE154763 rather than GSE160269 myeloid mixtures. Responses were summarised as the shift in mean score in the units of the baseline standard deviation and as the area under the receiver operating characteristic curve (AUROC) separating perturbed from unperturbed mixtures.

### 2.10. Prognostic Models and Incremental Value

Feature panels were fixed in advance. The clinical panel contained age, sex, histology, and stage indicators (seven variables). The ReCAST panel added eight compact features: four log-ratio balances (tumour versus microenvironment, immune versus stromal, myeloid versus lymphoid, and exhausted/regulatory versus conventional T cells), normalised state diversity, matched mass, the unknown-expression score, and the reconstruction cosine. A pathway panel added 24 outcome-independent MSigDB 2026.1 pathway scores [50,51,52], and a full panel combined all families. The 13 raw state fractions were used for interpretation only. SurvivalPFN, a prior-fitted tabular foundation model adapted to censored survival [53], was the prespecified primary backbone; elastic-net Cox regression, random survival forest, and XGBoost with an accelerated-failure-time objective were prespecified comparators [54,55,56,57,58], and SurvPFN [59] was evaluated on the clinical panel on the same splits.

Models were evaluated by ten repetitions of five-fold patient-level cross-validation (50 outer folds) with a locked split manifest; imputation, scaling, and hyperparameter selection were fitted within training partitions. The primary endpoint was Uno’s inverse-probability-of-censoring-weighted concordance index [60], with Harrell’s concordance, time-dependent AUC, an integrated Brier score (IBS), and calibration as secondary measures [61,62,63]. Incremental value was the paired within-fold difference between the clinical-plus-ReCAST and clinical panels on the same backbone. Outer folds of repeated cross-validation share patients and training data, so fold-level differences are not independent. Uncertainty was therefore expressed with the corrected repeated cross-validation *t* interval [64,65], in which the variance of the mean difference is inflated from *s*^2^/*J* to (1/*J* + *n*_test_/*n*_train_)*s*^2^ with *J* − 1 degrees of freedom, and with a patient-level cluster bootstrap that resamples the 182 patients while keeping all out-of-fold predictions fixed [66]. The number of repetitions in which the mean difference favoured each panel is also reported.

**Sensitivity analyses.** The paired contrast was repeated with Uno’s concordance restricted to event times within 1, 2 and 3 years, so that conclusions did not rest on sparse late follow-up, and within ESCC, EAC and recorded-stage patients using the locked out-of-fold predictions. Because models trained on the whole cohort could still be dominated by one histology, elastic-net Cox, the strongest clinical model, was also refitted under the same nested scheme within ESCC-only (94 patients, 32 deaths), EAC-only (88 patients, 45 deaths) and recorded-stage-only (171 patients, 69 deaths) cohorts, and with ReCAST features recomputed from the histology-matched reference (Section 2.6).

### 2.11. External Validation and Clinical Utility

External validation addressed one question: whether the clinical prediction models transfer to an independent cohort. In line with the prespecified protocol, it was applied to the two clinical models, SurvivalPFN and elastic-net Cox tuned within TCGA, and the June-2026 challenger SurvPFN was scored on the same patients. The incremental value of ReCAST features was established by paired internal comparison (Section 2.10), which used all 182 patients over 50 outer folds; a cohort of 60 patients and 33 deaths was suited to test whether a fixed model transferred, but not to resolve C-index differences in the size examined internally. Because the external cohort consists entirely of ESCC, the external evidence applies to that histology, and EAC is covered by the histology-specific analyses described in Section 2.6 and Section 2.10. Clinical models were refitted on the full TCGA cohort and scored once on the 60 non-overlapping GSE53625 patients, without recalibration or any use of external outcomes for model development. Discrimination and prediction error were reported with 5000 patient-level bootstrap intervals, calibration by intercept and slope at one and three years, and clinical utility by inverse-probability-of-censoring-weighted decision-curve analysis at one and three years [67,68].

### 2.12. Software and Reproducibility

Analyses were run in Python 3.12.14 (NumPy 2.5.3, SciPy 1.18.1, pandas 2.3.3, scikit-learn 1.9.1, scikit-survival 0.28.0, XGBoost 3.4.1, lifelines 0.30.3) and R 4.4.3 (MuSiC 1.0.0, BisqueRNA 1.0.5, DWLS 0.1.0, Seurat 5.5.1, BayesPrism 2.2.3, e1071 1.7.17) under fixed seeds. The pipeline records source-file hashes, the split manifest, pinned environments, and all per-fold and external predictions. The generative artificial-intelligence tool used during this work and its role are declared in the Acknowledgments Section.

## 3. Results

### 3.1. Study Overview and Cohorts

The analysis proceeded from the technical validation of cell-state estimation, through biological interpretation, to an independent assessment of prognostic value (Figure 1). The development cohort comprised 182 TCGA-ESCA primary tumours with 77 deaths, divided almost evenly between squamous-cell carcinoma (94) and adenocarcinoma (88). The locked external test set comprised the 60 GSE53625 patients confirmed absent from GSE53624, of whom 33 died; this set was entirely squamous-cell and had a higher event fraction than the development cohort (55.0% versus 42.3%), making it a demanding transportability test (Table 1). Median follow-up by the reverse Kaplan–Meier method was 608 days (about 20 months) in the development cohort and 1666 days (about 55 months) in the external cohort. In TCGA-ESCA, 69 of the 77 deaths occurred within three years and 17 patients remained at risk at three years (Table 1).

The reference summarised 216,332 cells from 67 donors into 965 donor-by-state profiles and 13 modelled states over 13,614 shared genes. Marker selection returned 326 genes for the TCGA-ESCA gene space. Fitting was well behaved: the relative change in state coefficients fell from 5.8 × 10^−2^ at the second reweighting iteration to 6.6 × 10^−4^ at the fifth and 1.8 × 10^−4^ at the sixth in pseudo-bulk mixtures, with the same profile in the 182 tumours (7.0 × 10^−2^ to 6.9 × 10^−4^), so the locked five iterations corresponded to converged estimates.

### 3.2. Accuracy of Cell-State Estimation

**Figure 3:**
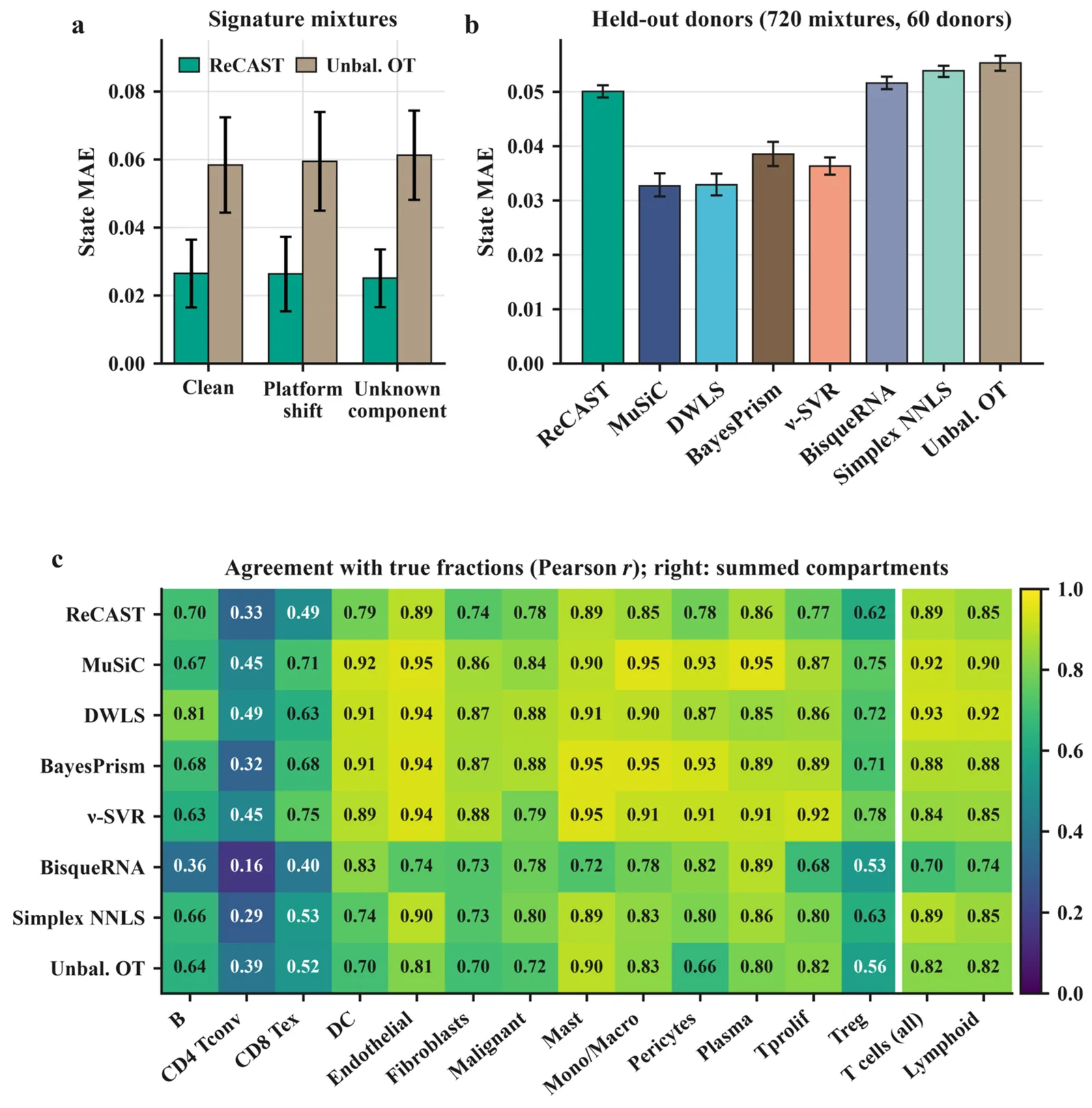
Accuracy of cell-state estimation. (**a**) Composition error on 500 synthetic signature mixtures per scenario for ReCAST and unbalanced optimal transport (mean ± SD). (**b**) Composition error on 720 pseudo-bulk mixtures summed from genuine cells of 60 held-out donors, with donor-clustered 95% confidence intervals, including BayesPrism and the ν-SVR core of CIBERSORT. (**c**) Per-state agreement (Pearson r) between estimated and true fractions across the held-out mixtures; the two right-hand columns sum the four T-cell states and the six lymphoid states.

On 500 synthetic mixtures per scenario, ReCAST recovered compositions with a mean absolute error (MAE) near 0.026 and was about twice as accurate as entropic unbalanced optimal transport (0.058–0.061), with lower Jensen–Shannon divergence and higher reconstruction fidelity in clean, platform-shift, and unknown-component scenarios (Figure 3a, Table 2).

Because mixtures generated from a signature can flatter the estimator that uses it, accuracy was then assessed on pseudo-bulk mixtures summed from genuine cells of donors withheld from anchor construction (720 mixtures, 60 donors). ReCAST achieved an MAE of 0.0501 (95% CI 0.0489–0.0512), more accurate than BisqueRNA (0.0516), simplex-constrained least squares on the same design (0.0538, paired difference 0.0038, 95% CI 0.0032–0.0043) and unbalanced optimal transport (0.0553, paired difference 0.0052, 95% CI 0.0036–0.0068). The two count-based reference methods were even more accurate: MuSiC reached 0.0327 and DWLS 0.0329, a paired advantage over ReCAST of 0.0174 (95% CI 0.0150–0.0197) and 0.0172 (0.0152–0.0192), respectively (Figure 3b, Table 2). BayesPrism and the ν-SVR core, added during review, performed in line with the other count-based methods (MAE 0.0385, 0.0363–0.0408, and 0.0363, 0.0347–0.0379), confirming that count-based estimation achieved the lowest errors when raw counts were available (Figure 3b, Table 2). Per state, ReCAST tracked true fractions closely for the states that dominate bulk tissue—endothelial cells (*r* = 0.89), mast cells (0.89), plasma cells (0.86), monocytes/macrophages (0.85), dendritic cells (0.79), pericytes (0.78), malignant cells (0.78), and fibroblasts (0.74)—and less closely for the T-cell subsets that shared most of their transcriptome (CD4Tconv 0.33, CD8Tex 0.49, Treg 0.62), an ordering shared by every method tested (Figure 3c). At the level of the T-cell compartment, estimates were accurate: summed into one compartment, ReCAST T-cell estimates agreed with the true fractions at r = 0.89 and lymphoid estimates at r = 0.85, close to MuSiC (0.92 and 0.90) and DWLS (0.93 and 0.92) (Figure 3c). The lower subset-level correlations therefore reflect the transcriptional similarity of conventional, exhausted, regulatory and proliferating T cells, a property shared by every method, and T-cell results are interpreted at the compartment level in this study.

### 3.3. Transfer Between Studies and to Measured Tumour Composition

**Figure 4:**
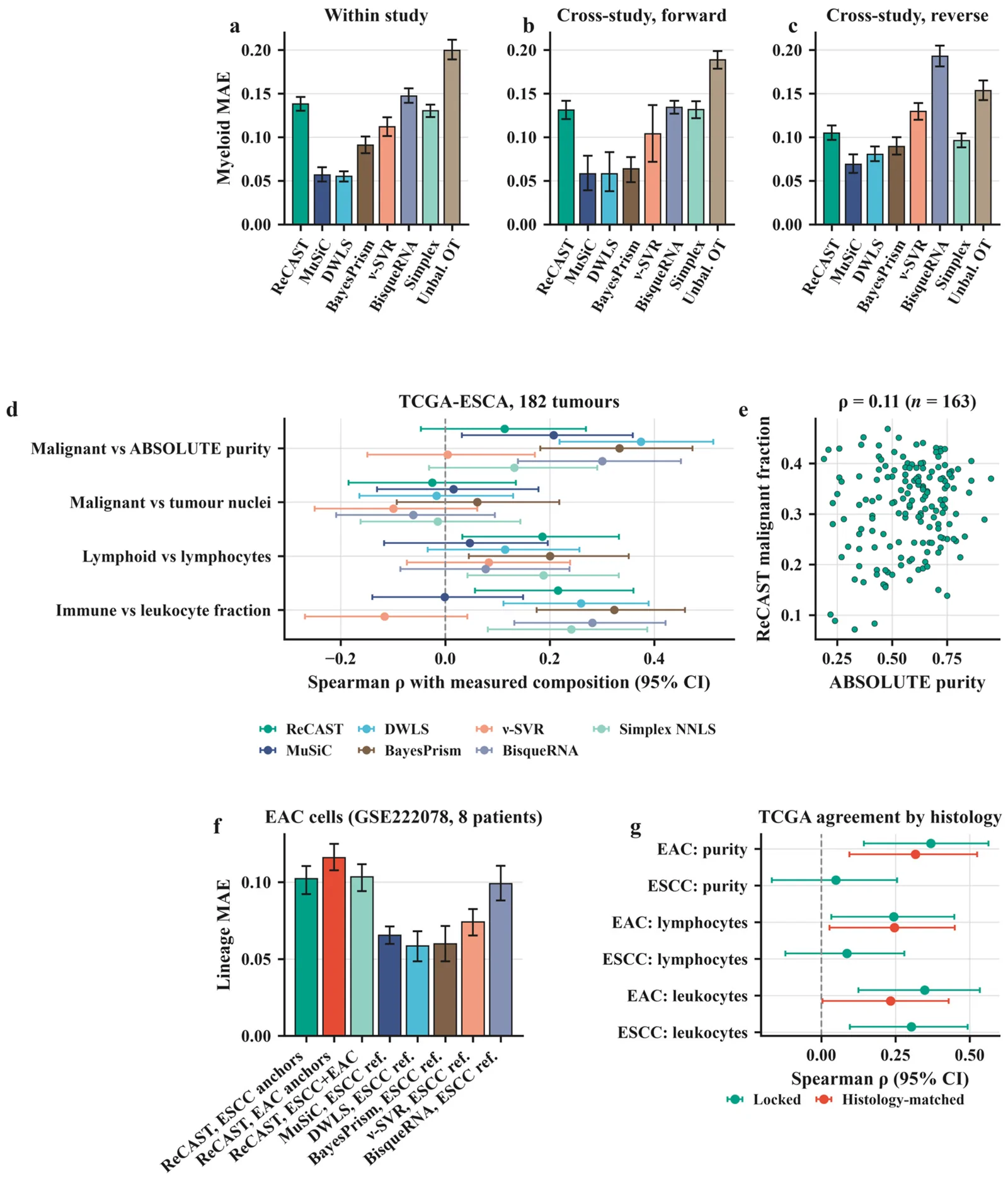
Transfer between studies and to measured composition. (**a**–**c**) Myeloid composition error for mixtures from held-out donors of the same study (**a**), for GSE154763 mixtures projected onto GSE160269 anchors (**b**), and for GSE160269 mixtures projected onto GSE154763 anchors (**c**). (**d**) Agreement of estimated composition with independently measured composition in 182 TCGA-ESCA tumours (Spearman rho, bootstrap 95% CI). (**e**) ReCAST malignant fraction against ABSOLUTE purity. (**f**) Lineage-level composition error on 240 pseudo-bulk mixtures of adenocarcinoma cells (GSE222078) projected onto ESCC-derived anchors, onto EAC anchors built from the other seven patients and onto combined anchors, and for count-based methods with an ESCC cell reference (patient-clustered 95% CI). (**g**) Agreement with measured composition within each histology for the locked and the histology-matched reference.

Anchors built from all GSE160269 donors were applied to mixtures of myeloid cells from GSE154763, a different cohort profiled in a different laboratory, with labels harmonised to dendritic cells, mast cells, and monocytes/macrophages. ReCAST recovered the relative myeloid composition with *r* = 0.83 and MAE 0.131, indistinguishable from its performance on the matched within-study control (MAE 0.138, *r* = 0.73), so transfer between studies cost nothing relative to the transfer between donors of the same study (Figure 4a,b). In the reverse direction, myeloid anchors built from GSE154763 recovered composition in GSE160269 mixtures with MAE 0.105 and *r* = 0.85 (Figure 4c). Both cross-study directions again ranked ReCAST above unbalanced optimal transport (0.189 and 0.153) and, in the reverse direction, above BisqueRNA (0.193), while MuSiC and DWLS remained more accurate (0.058–0.080). BayesPrism and the ν-SVR core reached 0.064 and 0.104 in the forward direction and 0.089 and 0.129 in the reverse direction (within-study control 0.091 and 0.112).

In the 182 TCGA-ESCA tumours, estimates were compared with composition measured independently of transcriptome deconvolution (Figure 4d,e, Table 3). ReCAST lymphoid estimates tracked pathology lymphocyte infiltration more closely than MuSiC, BisqueRNA and DWLS (ρ = 0.19, 95% CI 0.04–0.32; simplex least squares on the same design was equivalent at 0.19), and its immune estimates tracked the leukocyte fraction estimated by the TCGA Research Network (ρ = 0.22, 0.06–0.36) at a level comparable to BisqueRNA (0.28) and DWLS (0.26), whereas MuSiC showed no association on this measure (ρ = 0.00). For the malignant compartment, DWLS agreed most closely with ABSOLUTE purity (ρ = 0.38), followed by BisqueRNA (0.30) and MuSiC (0.21), while ReCAST reached ρ = 0.11 (−0.06 to 0.28); none of the methods, including ReCAST, agreed with the pathologist’s percentage of tumour cells recorded on the adjacent section (ρ between −0.21 and −0.07). Agreement with the measured immune content was therefore the more reproducible signal across methods in real bulk tumours. BayesPrism showed similar or stronger agreement (ABSOLUTE purity ρ = 0.33, leukocyte fraction 0.32, lymphocyte infiltration 0.20), whereas the ν-SVR core, which lacked the cross-platform correction that CIBERSORTx applied to single-cell signatures, agreed less closely in bulk tumours (0.00, −0.12 and 0.08; Table 3).

Anchors built from squamous-cell carcinoma transferred well to adenocarcinoma (Figure 4f,g, Supplementary Figure S13 and Table S14). In the independent EAC cohort, each patient’s immune-cell profiles correlated with the matching locked anchor as closely as profiles of held-out ESCC donors (median ρ 0.84–0.91, against 0.77–0.86), and the matching anchor ranked first in at least 75% of patients for every immune state. EAC malignant profiles were matched to the malignant anchor in every patient (median ρ 0.71, against 0.88 in ESCC), and EAC fibroblasts aligned most closely with the adjacent pericyte anchor, so the histology-related shift was confined to the malignant and stromal states. On 240 pseudo-bulk mixtures of EAC cells, the locked ESCC-derived anchors gave a lineage-level MAE of 0.102 (95% CI 0.092–0.111), lower than anchors built from the other seven EAC patients (0.116, 0.108–0.125) and similar to the combined ESCC and EAC anchors (0.103); at the level of the 13 states, the two references gave similar errors (0.057 and 0.054), close to the 0.050 obtained for held-out ESCC donors. ReCAST was among the most stable estimators across histologies: its state-level error rose by only 0.007 on moving from ESCC to EAC cells, against 0.006–0.046 for the count-based methods, of which DWLS and BayesPrism gave the lowest lineage-level errors (0.059 and 0.060). In TCGA-ESCA, agreement with the measured composition was clear in EAC, where the malignant fraction tracked ABSOLUTE purity at ρ = 0.37 (0.14 to 0.56) and the immune fraction tracked the leukocyte fraction at 0.35 (ESCC: 0.05 and 0.30), a pattern shared by the count-based methods (purity ρ 0.42–0.53 in EAC and 0.18–0.32 in ESCC). The locked reference therefore needed no histology-specific adjustment: replacing the malignant anchor of EAC tumours with one built from EAC cells gave a purity of ρ = 0.32, leukocyte fraction of 0.23 and a similar unknown-expression score (mean 0.436 against 0.433).

### 3.4. Contribution of Individual Design Components

**Figure 5:**
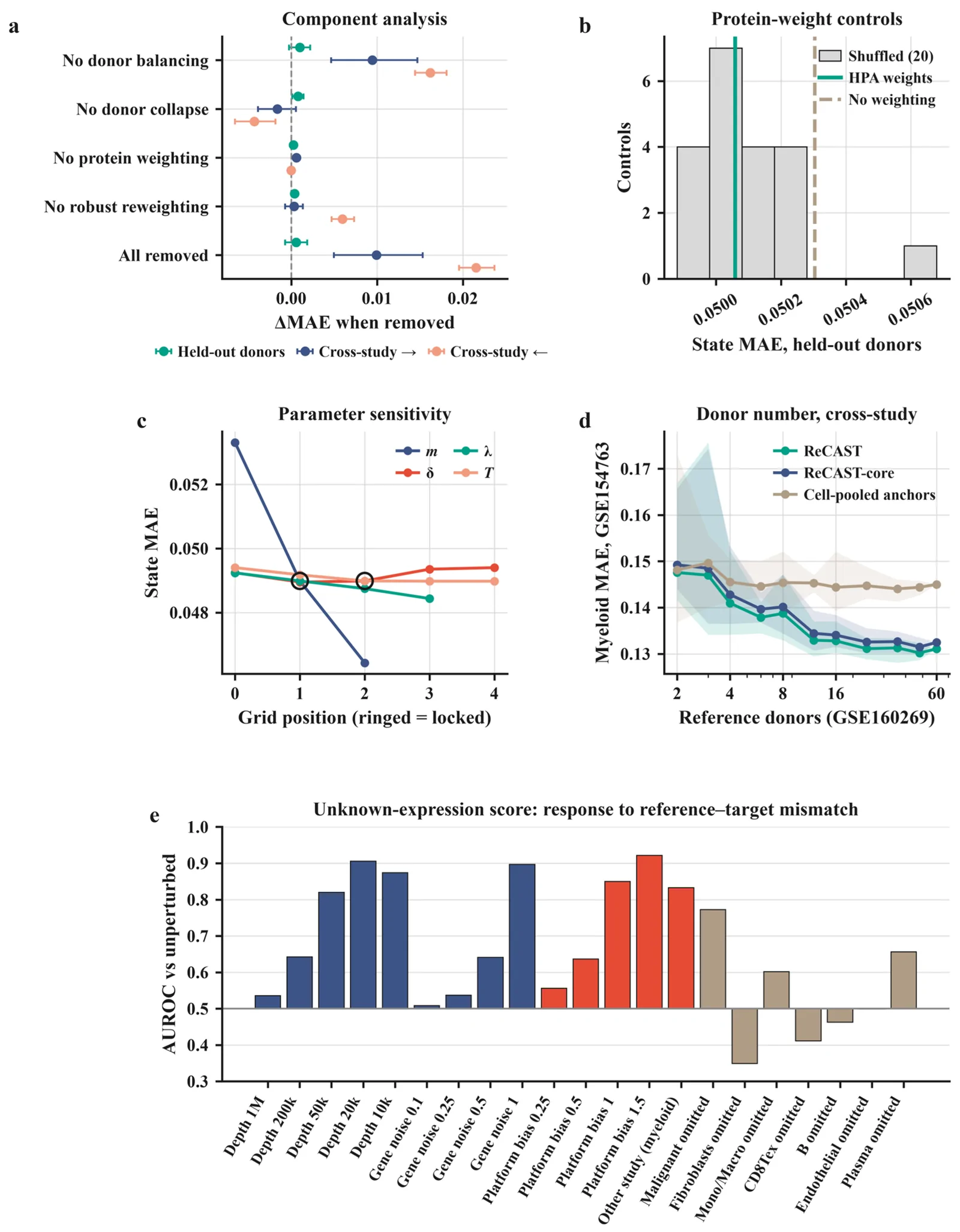
Design components and the unknown-expression score. (**a**) Change in composition error when each component is removed, on identical mixtures, with donor-clustered 95% confidence intervals; positive values indicate that the component improves accuracy. (**b**) Locked protein-evidence weights against 20 shuffled-weight controls and no weighting. (**c**) Sensitivity to marker number, Huber constant, weight exponent, and iteration number; the locked value of each parameter is ringed. (**d**) Cross-study composition errors (GSE160269 anchors applied to GSE154763 myeloid mixtures) when anchors are built from 2 to 60 reference donors (mean and 2.5th–97.5th percentiles over 10 draws per size) for locked ReCAST, the simplified ReCAST-core and cell-pooled anchors. (**e**) Discrimination of perturbed from unperturbed mixtures by the unknown-expression score; blue bars, technical noise (library depth and gene-wise noise); red bars, platform differences (gene-specific bias and another study); brown bars, omission of a state from the anchors.

Removing one component at a time from identical mixtures showed where the accuracy of the framework comes from (Figure 5a, Table 4). Donor-hierarchical aggregation was the component that mattered: replacing it with cell-count-weighted pooled anchors increased errors in every design—by 0.0010 (95% CI −0.0002 to 0.0022) in donor-held-out mixtures, 0.0094 (0.0047–0.0147) in the forward cross-study design, 0.0162 (0.0144–0.0181) in the reverse direction, and 0.0160 (0.0111–0.0210) in the within-study myeloid control; the benefit was therefore largest exactly where the reference and the target differed the most. Removing all three components (ordinary non-negative least squares on pooled anchors) produced the same pattern (up to 0.0215, 0.0195–0.0237). Huber reweighting contributed modestly and consistently in the cross-study reverse direction (0.0060, 0.0047–0.0073) and in the myeloid control (0.0019, 0.0008–0.0028). Protein-evidence weighting had no detectable effect on accuracy: removing it changed error by at most 0.0006, and the 20 shuffled-weight controls bracketed the locked value in every design (for example, donor-held-out MAE 0.0501 for the locked weights against 0.0499–0.0507 for shuffled weights, empirical *p* = 0.48). The reliability prior as implemented spans a narrow range (0.25–1.25 raised to λ = 0.25) and behaves as a near-neutral component of the estimator.

Accuracy was insensitive to the remaining parameters (Figure 5c). Across the grid, donor-held-out MAE varied only between 0.0464 and 0.0533: Huber constants of 1.0 to 2.0 and unreweighted fitting spanned 0.0490 to 0.0494; weight exponents of 0 to 1 spanned 0.0484 to 0.0492; five reweighting iterations were indistinguishable from ten or twenty (0.0490); and marker number was the only parameter with a visible effect, with the error decreasing from 0.0533 at 20 markers per state to 0.0490 at the locked 40 and 0.0464 at 80.

ReCAST was robust to reference size within a study and gained accuracy with reference size across studies (Figure 5d, Supplementary Figure S14 and Table S13). On held-out donors of the same study, mean MAE was 0.0491 with anchors built from two donors and 0.0497 with all 48 training donors, and additional donors made the anchors more stable, narrowing the 2.5th–97.5th percentile range across random donor subsets from 0.044 to 0.056 with two donors to 0.046–0.053 with eight and 0.049–0.050 with all donors. In cross-study transfer to GSE154763, accuracy improved steadily with the number of reference donors, from an MAE of 0.148 with two donors to 0.138 with six, 0.133 with 12 and 0.131 with all 60, whereas cell-pooled anchors remained near 0.145 at every size. The advantage of donor-hierarchical aggregation therefore grew with the reference, from 0.001 to 0.005 with two to four donors to 0.007 with six (favoured in 100% of draws) and 0.012–0.014 from 12 donors, while within a study the two constructions performed alike (differences below 0.002). The simplified ReCAST-core, which keeps donor-hierarchical anchors but omits protein weighting and Huber reweighting, was within 0.002 of the locked estimator in both settings.

### 3.5. The Recovered States Reproduce Independent Cell-Type Definitions

**Figure 6:**
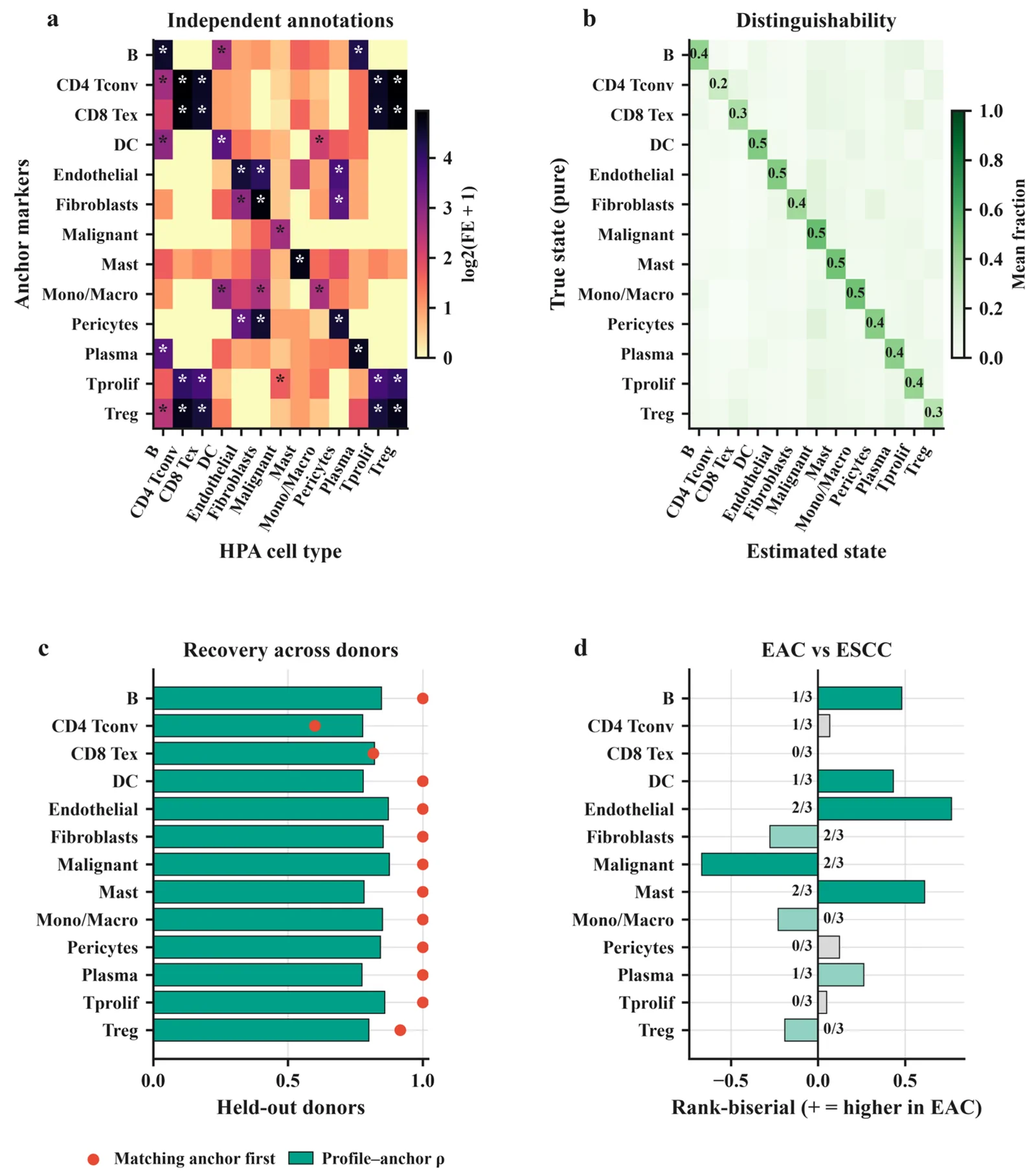
Biological interpretation of the recovered states. (**a**) Enrichment of each state’s anchor markers for independent Human Protein Atlas single-cell-type annotations (asterisks, FDR < 0.05). (**b**) Mean estimated composition of pure single-state pseudo-bulk samples from held-out donors. (**c**) Agreement between each held-out donor’s state profile and anchors built without that donor, and how often the matching anchor ranked first. (**d**) Differences in estimated composition between adenocarcinoma and squamous-cell carcinoma; labels give the number of established methods agreeing in direction at FDR < 0.05; dark teal bars mark states that differed at FDR < 0.05 and also replicated in random half-splits, light teal bars states that differed at FDR < 0.05 without replicating, and grey bars states that did not differ significantly.

Anchor markers were compared with Human Protein Atlas single-cell-type annotations, which were independent of the protein-existence evidence used for weighting (Figure 6a). Every state was enriched for markers annotated to its matching cell type (all FDR <10^−15^): 85% of malignant-state markers were annotated to esophageal epithelial or keratinocyte types, 82% of CD8Tex and 80% of CD4Tconv markers to T or NK cells, 75% of Mono/Macro markers to monocyte and macrophage types, and 65–70% of mast-cell, pericyte, plasma-cell, regulatory T-cell, and fibroblast markers to their matching types, with fold enrichments of 5.1 to 29.8. The enrichment matrix was diagonal apart from the expected overlap among T-cell states and between fibroblasts and pericytes.

States were also recovered consistently across donors and studies (Figure 6c). For every held-out donor, the donor’s own state profile correlated with anchors built without that donor at a median Spearman ρ of 0.77 to 0.88, and the matching anchor was the closest of the 13 anchors for 100% of donors in 10 of 13 states (CD4Tconv 60%, Treg 92%, CD8Tex 82%). Pure single-state samples from held-out donors were assigned to the correct state most often in 11 of 13 states (100% for malignant cells, dendritic cells, endothelial cells, mast cells, monocytes/macrophages, pericytes, and plasma cells; 70% for CD4Tconv), with a mean self-assignment of 0.24 to 0.52 of total mass and the residual mass distributed over transcriptionally adjacent states (Figure 6b). Myeloid marker selection replicated between the two independent studies: of 40 markers per state, 29 mast-cell, 23 dendritic-cell, and 20 monocyte/macrophage markers were selected in both studies against 0.13 expected by chance (all *p* < 10^−40^), and anchor rank profiles correlated at ρ = 0.96–0.97.

Estimated composition differed between histologies in a reproducible subset of states (Figure 6d). Endothelial (rank-biserial 0.77), malignant (−0.67, higher in ESCC), mast-cell (0.61), B-cell (0.48), and dendritic-cell (0.43) fractions differed at FDR < 10^−6^, and each of these five replicated in both halves of at least 99% of 500 random half-splits of the cohort; two to three of the established methods agreed in direction at FDR < 0.05 for the endothelial, malignant, mast-cell, and fibroblast contrasts. Differences in T-cell subsets and pericytes did not survive correction or did not replicate in half-splits and are not interpreted.

The histology contrasts were independent of driver alterations. None of the 208 associations between a state and an alteration reached a false-discovery rate of below 0.10 after adjustment for histology, and the EAC–ESCC differences in endothelial, malignant, mast-cell, B-cell and dendritic-cell fractions remained after adjustment for all 16 alterations together (all adjusted FDR < 0.001), with effect sizes changing by 16% at most (Supplementary Table S15). Within ESCC, cell-state composition differed between the C1 and C2 molecular subtypes in mast-cell, CD4Tconv and monocyte/macrophage fractions (FDR 0.034–0.046); EAC subtypes could not be compared because 78 of the 80 classified tumours were chromosomally unstable. The endothelial, malignant and B-cell differences were robust to the reference used for the EAC tumours, histology-matched or pooled, while the mast-cell and dendritic-cell differences varied with the reference and are interpreted with caution. These results are descriptive and support no causal or functional interpretation.

### 3.6. The Unknown-Expression Score Indicates Reference–Target Mismatch

The normalised residual behaved as a sensitive indicator of mismatch between the reference and the target, and its response was strongest for technical and platform differences (Figure 5e). Reducing library depth raised the score progressively (AUROC against unperturbed mixtures 0.54 at 10^6^ UMIs, 0.64 at 2 × 10^5^, 0.82 at 5 × 10^4^, and 0.91 at 2 × 10^4^), as did gene-wise multiplicative noise (AUROC 0.54 at a coefficient of variation of 0.25 and 0.90 at 1.0) and gene-specific platform bias (0.64 at σ = 0.5, 0.85 at σ = 1.0, 0.92 at σ = 1.5). Applying anchors across studies also raised the score relative to matched within-study mixtures (AUROC 0.83). Omitting a state from the anchors while it was present in mixtures produced a weaker and state-dependent response (AUROC 0.77 for malignant cells and 0.66 for plasma cells at a 50% spike; near 0.5 for the remaining states). The score is therefore best read as a quality-control signal for reference–target mismatch—depth, noise, and platform differences included—rather than as a measure of unrepresented biology, and it should not be interpreted as an unknown cell fraction. In the 182 TCGA-ESCA tumours, the score occupied a narrow range (mean 0.412, SD 0.028), consistent with a uniformly processed cohort.

### 3.7. Incremental Prognostic Value

**Figure 7:**
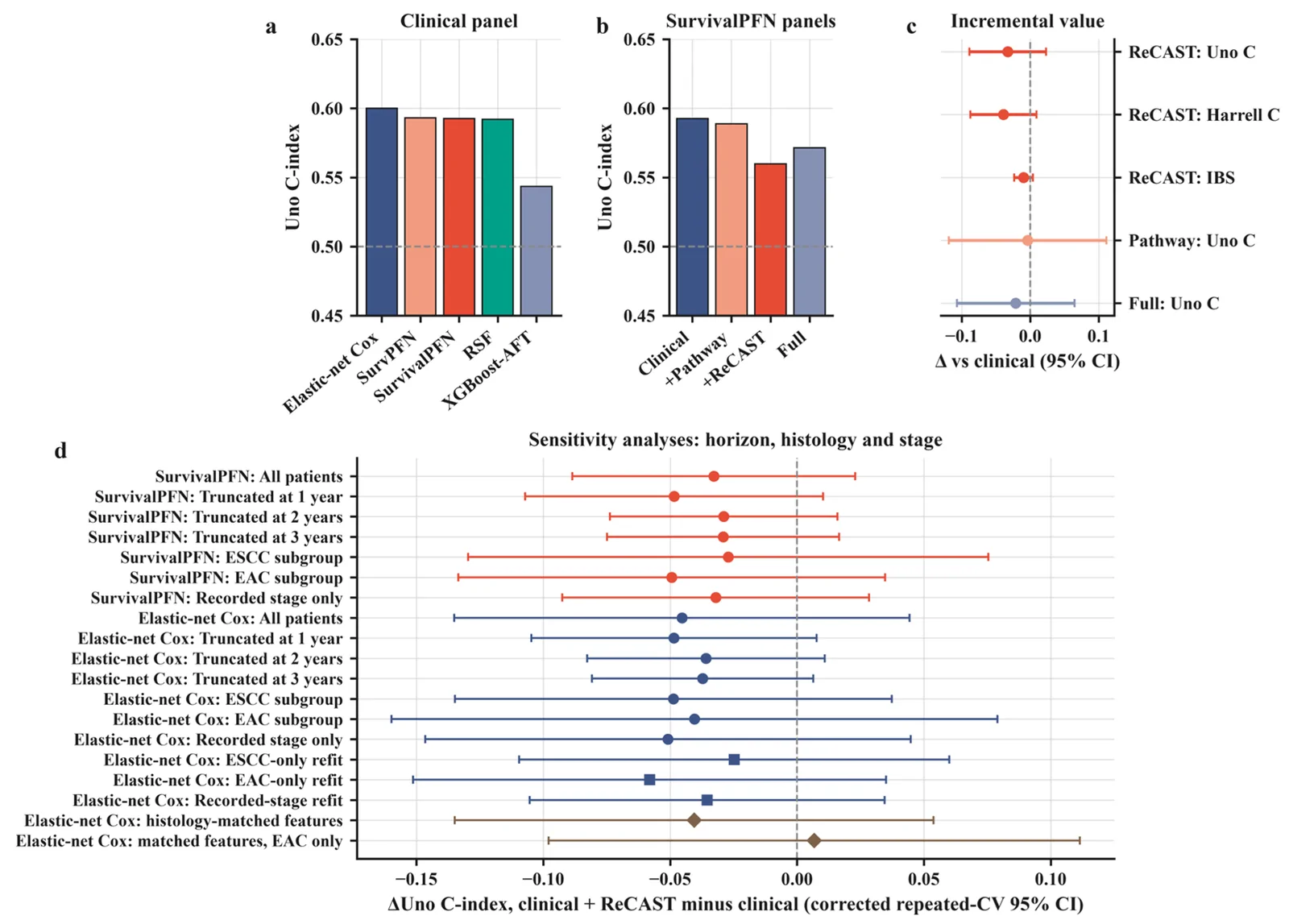
Prognostic evaluation over 50 outer folds. (**a**) Clinical-panel performance of the five prediction models. (**b**) Effect of feature panels on the SurvivalPFN backbone. (**c**) Paired within-fold differences relative to the clinical panel, with corrected repeated cross-validation 95% confidence intervals. (**d**) Sensitivity analyses: discrimination truncated at 1, 2 and 3 years, subgroup evaluation of the locked predictions, elastic-net Cox refitted within ESCC-only, EAC-only and recorded-stage-only cohorts, and features from the histology-matched reference; red, SurvivalPFN; dark blue, elastic-net Cox; brown, elastic-net Cox with features from the histology-matched reference; circles, locked out-of-fold predictions; squares, elastic-net Cox refitted within each cohort; diamonds, histology-matched features.

On the clinical panel of seven routinely recorded variables, the five prediction models occupied a narrow band over the 50 outer folds: elastic-net Cox achieved the highest mean Uno C-index (0.600), followed by SurvivalPFN and the June 2026 SurvPFN challenger (both 0.593) and random survival forest (0.592), with XGBoost-AFT lowest (0.544); random survival forest achieved the lowest integrated Brier score (0.221), ahead of SurvivalPFN (0.224) and elastic-net Cox (0.227) (Figure 7a, Table 5). Clinical variables alone therefore achieved the strongest performance among the evaluated models, and this is the level against which any additional feature family is judged.

Adding the compact ReCAST features to the same backbone did not improve prediction (Figure 7b,c, Table 6). Mean Uno C-index moved from 0.593 to 0.560 and the integrated Brier score from 0.224 to 0.234. Because outer folds of repeated cross-validation share patients and training data, the paired difference was summarised with the corrected repeated cross-validation interval: ΔUno C = −0.033 (95% CI −0.089 to 0.023; *p* = 0.24), ΔHarrell C = −0.039 (−0.087 to 0.009), and ΔIBS = −0.010 (−0.023 to 0.004). The direction was consistent—the mean difference favoured the clinical panel in all 10 repetitions, and a patient-level cluster bootstrap gave −0.033 (−0.066 to −0.000)—but the dependence-aware interval included zero, so the evidence supported no gain rather than a demonstrated loss. Pathway scores were neutral (ΔUno C = −0.004, −0.119 to 0.111) and the full feature set fell between the two (−0.021, −0.107 to 0.065). The same comparison on elastic-net Cox gave ΔUno C = −0.045 (−0.135 to 0.044). The prespecified criterion for incremental value, a positive paired difference with an interval excluding zero, was not met. Because the representation had already been validated against known composition, independent annotations, and measured tumour composition, and because no outcome information entered feature construction, this contrast reflects the relationship between cell-state composition and survival in this cohort rather than a failure of the measurement step.

This result was robust across follow-up horizons, histologies and staging completeness (Figure 7d, Supplementary Table S16). With discrimination restricted to events within 1, 2 and 3 years, the SurvivalPFN difference was −0.048 (−0.107 to 0.010), −0.029 (−0.074 to 0.016) and −0.029 (−0.075 to 0.017). In the locked out-of-fold predictions, it was −0.027 (−0.130 to 0.075) in ESCC, −0.049 (−0.134 to 0.035) in EAC and −0.032 (−0.093 to 0.028) in patients with recorded stage. Refitting elastic-net Cox inside each cohort gave −0.025 (−0.110 to 0.060) for ESCC only, −0.058 (−0.151 to 0.035) for EAC only and −0.035 (−0.105 to 0.035) for recorded stage only, and features recomputed from the histology-matched reference gave −0.041 (−0.135 to 0.054) overall and 0.007 (−0.098 to 0.112) in EAC. Every interval included zero, confirming that the main conclusion held independently of histology, staging completeness and length of follow-up.

### 3.8. External Validation and Clinical Utility of the Clinical Models

**Figure 8:**
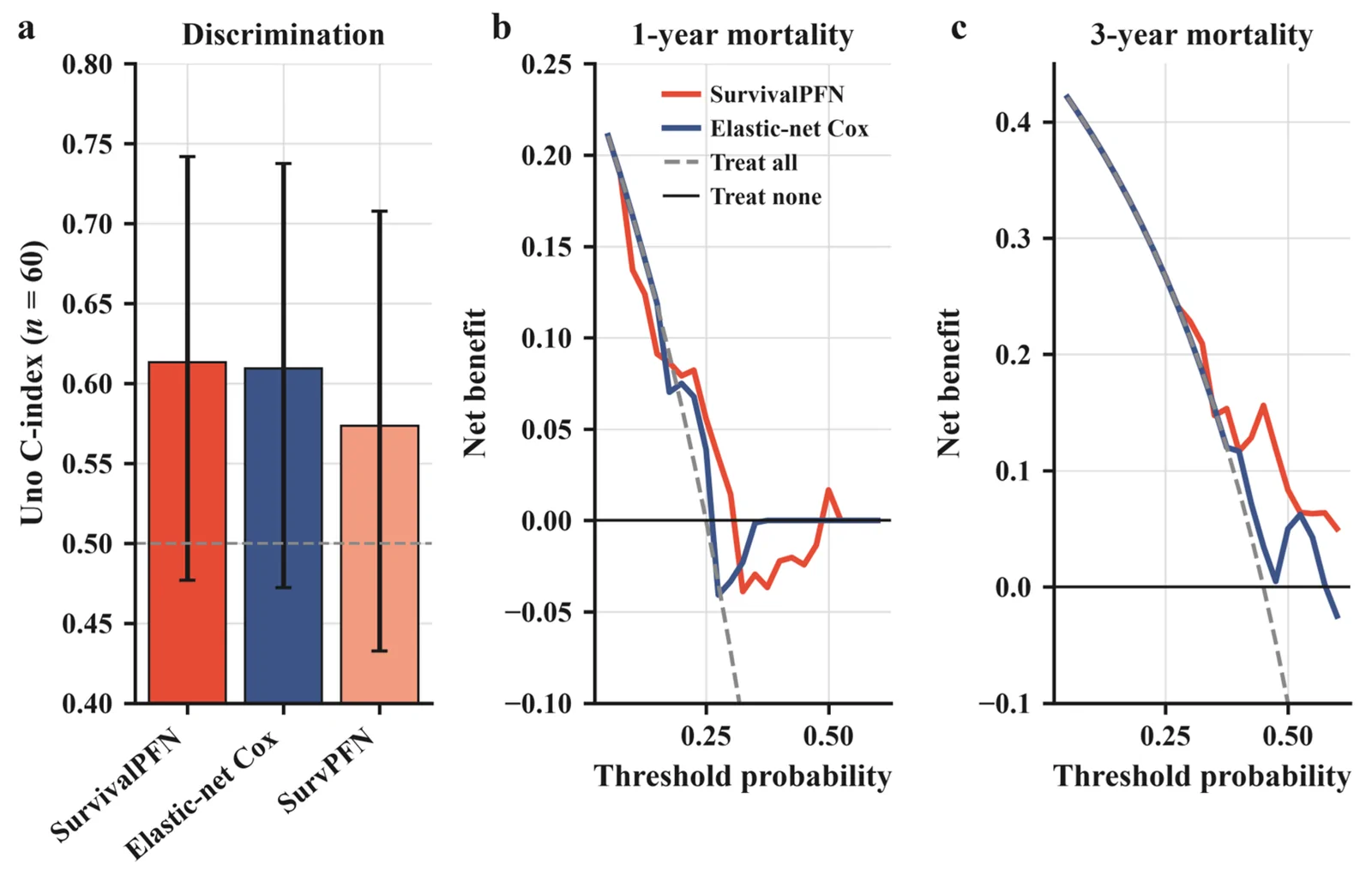
Locked external validation of the clinical prediction models on 60 non-overlapping GSE53625 patients with ESCC. (**a**) Discrimination with bootstrap 95% confidence intervals. (**b**,**c**) Decision-curve analysis at the prespecified one-year (**b**) and three-year (**c**) horizons against treat-all and treat-none.

Refitted on the complete development cohort and scored once on the 60 non-overlapping external patients, the clinical models retained their discrimination in a cohort of different histological composition and event rate (Figure 8a, Table 7). SurvivalPFN reached a Uno C-index of 0.613 (bootstrap 95% CI 0.477–0.742) and elastic-net Cox 0.609 (0.472–0.738), with the June 2026 challenger at 0.574 (0.433–0.708); all point estimates exceeded chance, and the intervals are wide at this sample size, so the ranking among models is not resolved. Time-dependent AUC was higher at three years than at one year for both leading models (SurvivalPFN 0.631 versus 0.586). Calibration slopes at one year were 0.45 for SurvivalPFN and 0.59 for elastic-net Cox, indicating that predicted risks were more extreme than the observed risks; because recalibration was deliberately withheld from the locked test set, these values describe out-of-the-box transfer, and recalibration estimated outside the test set is the step required before absolute risks are reported. Discrimination within the development cohort was carried mainly by the squamous-cell subgroup (Uno C 0.646 for SurvivalPFN) relative to adenocarcinoma (0.536), with no risk-by-histology interaction surviving correction (all FDR ≥ 0.13).

The decision-curve analysis placed the clinical models above both default strategies over a clinically relevant range at the longer horizon (Figure 8b,c). At three years, SurvivalPFN exceeded treat-all and treat-none across threshold probabilities of approximately 0.30 to 0.60 and elastic-net Cox across 0.40 to 0.58; at one year, the advantage was confined to a narrower band around 0.20 to 0.30. The analysis is exploratory in the sense that thresholds were fixed in advance but not chosen with clinicians.

## 4. Discussion

This study separated two questions that are usually answered together: how accurately a cell-state projection measures tumour composition, and whether the resulting features improve prognosis. Both were answered with independent evidence, and the answers differed.

On the measurement question, ReCAST is a measurement tool with defined operating characteristics, best supported for the immune and stromal compartments. Its state anchors reproduce independent cell-type definitions: markers selected without any outcome or annotation input were enriched for Human Protein Atlas single-cell-type annotations in all 13 states, with 47–85% of markers per state annotated to the matching type and fold enrichments up to 30. The same states were recovered when donors were withheld, when anchors were rebuilt from an entirely different study, and when applied to mixtures assembled from another cohort’s cells and myeloid markers replicated across the two studies far beyond chance. On genuine-cell mixtures from held-out donors, ReCAST estimated composition more accurately than BisqueRNA, simplex-constrained least squares on the same design, and entropic unbalanced optimal transport, and it tracked measured lymphocyte infiltration and leukocyte fraction in real tumours as closely as MuSiC, BisqueRNA and DWLS, with BayesPrism, added during review, reaching similar or higher values. For the malignant compartment, purity estimates from copy-number data or count-based methods remain preferable (ReCAST ρ = 0.11, DWLS 0.38), so the ReCAST malignant fraction is best used for composition profiling rather than as a purity measure. Two properties make the framework practical: it needs only summarised anchor profiles rather than cell-level data at projection time, which allows references to be shared where cell-level data cannot be; and because it operates on within-sample ranks, it applies unchanged to count-derived, variance-stabilised, or array-based expression, whereas count-based deconvolution requires raw counts.

The controlled component analysis identified which design choice did the work. Donor-hierarchical aggregation—summarising within donor before combining donors—was the component that mattered, and its benefit was the largest precisely where the reference and target differed most, reducing errors by 0.009 to 0.016 in a cross-study transfer against 0.001 within a study. This is a general point for reference construction: pooling cells directly lets deeply sampled donors define a state, and the resulting anchors transfer less well. The donor-number simulation shows where this design pays off. Within a single study, donor balancing changed held-out errors by only 0.001 and donor-hierarchical and cell-pooled anchors performed alike at every reference size, whereas across studies, the benefit appeared consistently from about six reference donors and approached its full size from about 12. ReCAST is therefore best used with references of six or more donors, with at least two per state being required by construction, and the simplified ReCAST-core performs as well as the full estimator, offering a lighter implementation for routine use. Huber reweighting gave a smaller consistent gain in the most dissimilar transfer setting. Protein-evidence weighting, by contrast, was neutral in every design, and 20 shuffled-weight controls bracketed the locked value; as implemented over a narrow reliability range, this prior neither helps nor harms, and a stronger external prior would be needed for it to contribute. Reporting this explicitly, rather than assuming that each component earns its place, is what the component analysis was for. Accuracy was otherwise stable across the parameter grid, and the estimator converged well within the locked five iterations, so the framework’s behaviour did not depend on fine parameter choices. Where accuracy mattered most and raw counts were available, MuSiC, DWLS, BayesPrism and the ν-SVR core of CIBERSORT achieved lower errors than the rank-based projection, and they were the natural choice in that setting; the complementary strengths observed here—ReCAST for data that were not counts and for immune profiling across studies, count-based methods, and BayesPrism in particular, for overall composition and purity when raw counts were available—suggest that reporting more than one estimator is prudent in studies where composition was a primary endpoint.

The unknown-expression score was characterised rather than assumed. It responded strongly and monotonically to sequencing depth, gene-wise noise, platform bias, and the use of another study’s data and only weakly to omission of a state from the anchors. It is therefore a reference–target mismatch indicator, useful as a quality-control signal when a reference is transported to a new platform or cohort, and it should not be reported as an unknown cell fraction. Naming what such a residual actually measures is a small but transferable point for deconvolution methods that retain one.

On the clinical question, the answer is equally clear. Seven routinely recorded variables achieved the strongest performance among the evaluated models, transferred to an independent cohort with a Uno C-index of 0.61, and showed net benefit over both default strategies across the threshold range relevant to three-year surveillance decisions. The cell-state features, whose measurements had been checked independently, added no further prognostic information: the paired difference was −0.033 in Uno C-index, favoured the clinical panel in all ten repetitions, and its dependence-aware interval included zero. The interval matters. Treating the 50 outer folds of repeated cross-validation as independent observations produces intervals roughly three times narrower than the corrected interval, and would have licensed a confident claim of harm from the same data. Analyses that compare feature panels across repeated cross-validation should report dependence-aware uncertainty, whichever direction the point estimate takes.

That a biologically faithful representation adds nothing to a clinical model is informative rather than contradictory. Cell-state composition and stage capture overlapping prognostic information in this disease, and the states estimated here are averages over a resected tumour, which may be too coarse a summary to refine risk beyond stage in a cohort of 182 patients and 77 events. That the design allows that conclusion to be drawn at all is because the representation had cleared independent accuracy and interpretation checks before any outcome model was fitted, and because no outcome information reached feature construction, the null result was attributable to the biology-to-outcome relationship rather than to an untested preprocessing step. Transcriptome-derived immune scores have been built into prognostic models in other tumours, for example, the ssGSEA-based immune model in glioblastoma [19]; the present results suggest that such scores should also be judged by what they add to established clinical predictors, not only by their association with outcome. The same sequence—validate the measurement against known composition, independent annotations, and measured composition, then test incremental value against established clinical predictors with dependence-aware uncertainty and external evaluation—applies to any molecular biomarker proposed for prognosis, and is inexpensive relative to the cost of a spurious prognostic claim.

The limitations of this study mainly concern its scope. The main single-cell reference was derived from ESCC, and its transfer to EAC was therefore confirmed in an independent EAC cohort, in which immune anchors transferred well; with eight patients and relatively few malignant cells, larger EAC references will refine the malignant and stromal anchors further. The external validation cohort comprised ESCC, so external confirmation in EAC, supported here by EAC-only internal analyses, is the natural next step. Malignant-cell estimates are best complemented by copy-number purity, T-cell results are reported at the compartment level, and tissue-level measurement by multiplex imaging or spatial transcriptomics would extend the validation further. Finally, with a median follow-up of about 20 months, the development cohort mainly informs risk over the first three years, the horizon over which the time-truncated analyses confirmed the main result.

Two studies should come first. The incremental-value test should be repeated in a multi-institutional cohort of several hundred patients with uniform staging and both histologies, including an external adenocarcinoma cohort, so that a modest increment could be detected or excluded with adequate power. Spatial transcriptomic or multiplex imaging studies of the same tumours should then measure the proportions of the major populations, such as malignant cells, CD8 T cells, macrophages and fibroblasts, and their spatial organisation directly, and test whether region-specific composition carries prognostic information that a whole-tumour average does not; patient-derived organoid and co-culture models, which preserve much of the cellular heterogeneity of the parent tumour, offer a controlled setting in which reference-based estimates can be checked against known compositions [69]. The reliability prior generalises to any external, target-independent gene annotation, and a prior with a wider effective range is a natural next test given the neutrality observed here. Finally, because ReCAST needs only summarised anchors and operates on ranks, it is directly applicable to archival array-based cohorts where immune compositions can be estimated in data that count-based methods cannot use.

## 5. Conclusions

As a measurement tool, ReCAST estimated cell-state composition from bulk tumour expression reproducibly across donors, independent studies and both histologies. Its anchors reproduced independent cell-type definitions, its immune estimates agreed with measured composition in real tumours, and donor-hierarchical aggregation made transfers between studies more accurate. Because it works on within-sample ranks, ReCAST extends cell-state profiling to variance-stabilised, microarray and other non-count data, and to settings in which only summarised references can be shared, complementing count-based methods, which are preferred when raw counts are available. The normalised residual it reports provides a quality-control signal for reference–target mismatch. As a prognostic biomarker, cell-state composition added no information beyond age, sex, histology and stage in 182 esophageal cancers, a result that held within each histology and with an adenocarcinoma-matched reference, while the clinical models transferred to an independent squamous-cell carcinoma cohort. Because the measurement was validated before any outcome model was fitted, this finding reflects the biology of the disease rather than the method, and it provides a clear benchmark for future tumour microenvironment biomarkers. The next priority is a multi-institutional cohort that includes both histologies and an external adenocarcinoma cohort, studied together with spatial transcriptomic profiling of the same tumours, to test whether region-specific cell-state composition carries a prognostic value that whole-tumour averages do not.

## Figures and Tables

**Table 1:** Characteristics of the TCGA-ESCA development cohort and the locked external validation cohort.

Characteristic	TCGA-ESCA (Development)	GSE53625 (Locked External)
Patients, *n*	182	60
Deaths, *n* (%)	77 (42.3)	33 (55.0)
Age, median (IQR), years	60.7 (54.3–71.9)	60.2 (53.9–66.3)
Male, *n* (%)	158 (86.8)	48 (80.0)
ESCC, *n* (%)	94 (51.6)	60 (100.0)
EAC, *n* (%)	88 (48.4)	0 (0.0)
Stage I/II/III/IV, *n*	20/76/67/8	4/30/26/0
Stage unknown, *n*	11	0
Observed time, median (IQR), days	396.5 (231.2–675.8)	1197.2 (385.3–1620.0)
Follow-up, median (reverse Kaplan–Meier), days	608	1666

Stage unknown: No AJCC stage recorded in any source (*n* = 10) or M category not assessed (*n* = 1); these patients were retained with an unknown-stage indicator (Section 2.2).

**Table 2:** Accuracy of cell-state estimation across validation designs. Values are mean absolute errors between estimated and true state fractions with donor-clustered 95% confidence intervals; lower is better. Cross-study designs are evaluated on the harmonised myeloid states.

Method	Signature Mixtures (1500)	Donor-Held-Out (720)	Cross-Study → GSE154763 (280)	Cross-Study → GSE160269 (240)
ReCAST	0.0250–0.0265	0.0501 (0.0489–0.0512)	0.1310 (0.1207–0.1418)	0.1048 (0.0969–0.1135)
MuSiC	not applicable	0.0327 (0.0307–0.0350)	0.0580 (0.0391–0.0786)	0.0687 (0.0589–0.0803)
DWLS	not applicable	0.0329 (0.0309–0.0349)	0.0580 (0.0381–0.0828)	0.0803 (0.0724–0.0892)
BayesPrism	not applicable	0.0385 (0.0363–0.0408)	0.0636 (0.0484–0.0771)	0.0893 (0.0799–0.1000)
ν-SVR (CIBERSORT core)	not applicable	0.0363 (0.0347–0.0379)	0.1039 (0.0717–0.1367)	0.1294 (0.1200–0.1392)
BisqueRNA	not applicable	0.0516 (0.0505–0.0527)	0.1342 (0.1269–0.1416)	0.1929 (0.1810–0.2050)
Simplex NNLS (ReCAST design)	not applicable	0.0538 (0.0527–0.0548)	0.1317 (0.1217–0.1411)	0.0961 (0.0884–0.1043)
Unbalanced optimal transport	0.0583–0.0612	0.0553 (0.0538–0.0566)	0.1886 (0.1785–0.1989)	0.1534 (0.1426–0.1651)

Signature mixtures are the range of scenario means (clean, platform shift, unknown component); count-based methods require raw counts and were not applied to that design. BayesPrism and the ν-SVR core of CIBERSORT were added during peer review; the cross-study columns use the same myeloid mixtures as the other methods.

**Table 3:** Agreement of estimated composition with independently measured composition in 182 TCGA-ESCA tumours (Spearman ρ with bootstrap 95% CI).

Measured Quantity (*n*)	ReCAST	MuSiC	DWLS	BisqueRNA	Simplex NNLS	BayesPrism	ν-SVR
ABSOLUTE purity (163)	0.11 (−0.06, 0.28)	0.21 (0.05, 0.36)	0.38 (0.22, 0.52)	0.30 (0.13, 0.45)	0.13 (−0.02, 0.29)	0.33 (0.18, 0.47)	0.00 (−0.15, 0.17)
Pathology tumour nuclei (169)	−0.03 (−0.19, 0.13)	0.02 (−0.15, 0.17)	−0.02 (−0.17, 0.14)	−0.06 (−0.21, 0.09)	−0.01 (−0.17, 0.15)	0.06 (−0.09, 0.22)	−0.10 (−0.25, 0.06)
Pathology lymphocyte infiltration (169)	0.19 (0.04, 0.32)	0.05 (−0.11, 0.21)	0.11 (−0.03, 0.26)	0.08 (−0.07, 0.24)	0.19 (0.04, 0.34)	0.20 (0.05, 0.35)	0.08 (−0.07, 0.24)
Estimated leukocyte fraction (171)	0.22 (0.06, 0.36)	0.00 (−0.15, 0.15)	0.26 (0.12, 0.40)	0.28 (0.13, 0.43)	0.24 (0.09, 0.38)	0.32 (0.18, 0.46)	−0.12 (−0.27, 0.04)

Malignant fraction is compared with purity and tumour-nuclei measures, lymphoid fraction with lymphocyte infiltration, and combined lymphoid plus myeloid fraction with the leukocyte fraction. Bold marks the strongest agreement per row.

**Table 4:** Controlled component analysis. Values are the change in state MAE when a component is removed, with a donor-clustered 95% CI; positive values indicate that the component improves accuracy.

Component Removed	Donor-Held-Out	Within-Study Myeloid	Cross-Study → GSE154763	Cross-Study → GSE160269
Donor balancing (cell-pooled anchors)	0.0010 (−0.0002, 0.0022)	0.0160 (0.0111, 0.0210)	0.0094 (0.0047, 0.0147)	0.0162 (0.0144, 0.0181)
Donor collapse (profile median)	0.0008 (0.0001, 0.0014)	−0.0021 (−0.0034, −0.0009)	−0.0016 (−0.0039, 0.0005)	−0.0043 (−0.0065, −0.0018)
Protein-evidence weighting	0.0002 (0.0001, 0.0004)	−0.0002 (−0.0009, 0.0004)	0.0006 (0.0003, 0.0010)	0.0000 (0.0000, 0.0000)
Huber reweighting	0.0004 (0.0001, 0.0006)	0.0019 (0.0008, 0.0028)	0.0003 (−0.0007, 0.0013)	0.0060 (0.0047, 0.0073)
All three components	0.0006 (−0.0007, 0.0019)	0.0157 (0.0112, 0.0205)	0.0099 (0.0050, 0.0153)	0.0215 (0.0195, 0.0237)

**Table 5:** Internal performance of the clinical panel over 10 × 5-fold nested cross-validation (50 outer folds), ranked by mean Uno C-index.

Model	Harrell C (Mean ± SD)	Uno C (Mean ± SD)	Integrated Brier (Mean ± SD)
Elastic-net Cox	0.599 ± 0.077	0.600 ± 0.088	0.227 ± 0.082
SurvivalPFN	0.615 ± 0.081	0.593 ± 0.097	0.224 ± 0.084
SurvPFN (June 2026)	0.592 ± 0.074	0.593 ± 0.088	0.238 ± 0.065
Random survival forest	0.581 ± 0.069	0.592 ± 0.085	0.221 ± 0.075
XGBoost-AFT	0.570 ± 0.083	0.544 ± 0.092	0.236 ± 0.108

**Table 6:** Incremental value of feature panels on the SurvivalPFN backbone. Paired differences are relative to the clinical panel on identical folds, with corrected repeated cross-validation 95% CIs.

Feature Panel	Uno C (Mean ± SD)	Integrated Brier	Paired ΔUno C (95% CI)	Repetitions Favouring Clinical
Clinical only	0.593 ± 0.097	0.224 ± 0.084	reference	–
Clinical + pathway	0.589 ± 0.096	0.226 ± 0.089	−0.004 (−0.119, 0.111)	6 of 10
Clinical + ReCAST	0.560 ± 0.118	0.234 ± 0.091	−0.033 (−0.089, 0.023)	10 of 10
Full feature set	0.571 ± 0.091	0.230 ± 0.096	−0.021 (−0.107, 0.065)	7 of 10

**Table 7:** Locked external validation of the clinical prediction models on the 60 non-overlapping GSE53625 patients with ESCC (33 deaths).

Model	Harrell C	Uno C (95% CI)	Integrated Brier	Calibration Slope (1 y)
SurvivalPFN	0.598	0.613 (0.477, 0.742)	0.509	0.45
Elastic-net Cox	0.582	0.609 (0.472, 0.738)	0.567	0.59
SurvPFN (June 2026)	0.544	0.574 (0.433, 0.708)	0.492	not available

## Data Availability

All data analysed in this study are publicly available. TCGA-ESCA expression, clinical, and composition annotations are available from the NCI Genomic Data Commons (https://portal.gdc.cancer.gov/, accessed on 13 June 2026). Single-cell references and external cohorts are available from the NCBI Gene Expression Omnibus under accessions GSE160269, GSE154763, GSE222078, GSE53624, and GSE53625. Somatic mutation calls for TCGA-ESCA were obtained from cBioPortal (study esca_tcga_pan_can_atlas_2018). Gene sets were obtained from MSigDB (2026.1) and protein-evidence and single-cell-type annotations from the Human Protein Atlas. The analysis code, locked configuration files, split manifests, revision analysis scripts, and generated results are available at https://github.com/nahlama/ReCAST.git, accessed on 13 June 2026.

## Supplementary Materials

The following supporting information can be downloaded at https://www.mdpi.com/article/10.3390/biomedicines14102298/s1. Figure S1. Data-resource inclusion and independence audit: development, reference, and locked external cohorts, with the leakage boundary. Figure S2. Composition of the donor-aware reference: cells per state, contributing donors per state, and profile-size distribution. Figure S3. Full simulated-mixture error distributions (500 mixtures per scenario): state MAE, Jensen-Shannon divergence, reconstruction cosine, and unknown-component recovery. Figure S4. Per-fold internal metric distributions across models on the clinical panel (Uno C, integrated Brier score, 3-year AUC). Figure S5. Time-specific discrimination, prediction error, and calibration slope at 1, 3, and 5 years. Figure S6. Complete marker-stability and exploratory-association analysis: selection-frequency distribution, stable pairs per state, and gene and pathway volcanoes. Figure S7. External-cohort overlap audit and platform-annotation traceability. Figure S8. Donor-held-out projection: accuracy and rank fidelity across estimators, with paired comparisons against robust NNLS. Figure S9. Versioned external molecular reannotation against GENCODE 50 with gene- and pathway-level confirmation. Figure S10. Exploratory IPCW decision-curve analysis, internal (50-fold mean) and external, at 1 and 3 years. Figure S11. Histology-subgroup discrimination and transferred external risk-group calibration. Figure S12. PROBAST+AI author risk-of-bias and applicability self-assessment by domain. Figure S13. Independent adenocarcinoma single-cell cohort (GSE222078). (a) Cells per state and patient after quality control and marker-based annotation; (b) standardised marker scores of the assigned clusters; (c) agreement of each EAC patient′s state profile with the locked ESCC-derived anchors, against held-out ESCC donors. Figure S14. Reference donor-number simulation. Composition error over random draws of reference donors (10 per size) for locked ReCAST (green), the simplified ReCAST-core (blue) and cell-pooled anchors (taupe): (a) held-out donors of the same study (five folds); (b) transfer of GSE160269 anchors to GSE154763 myeloid mixtures. Table S1. Clinical-adjusted exploratory survival associations for the 92 prominent marker genes; none passed FDR < 0.05. Table S2. Clinical-adjusted exploratory survival associations for the 24 prespecified pathways; none passed FDR < 0.05. Table S3. Donor-held-out projection validation of the reference estimator. Table S4. Projection-estimator comparators on donor-held-out mixtures. Table S5. External gene-level confirmation after GENCODE 50 reannotation. Table S6. External pathway-level confirmation after reannotation. Table S7. Exploratory external performance within histology subgroups. Table S8. Histology x risk-score interaction tests. Table S9. External risk-group calibration using TCGA-derived tertile cutpoints. Table S10. PROBAST+AI author risk-of-bias self-assessment by domain. Table S11. Follow-up maturity (a) and the 11 patients without an assignable stage (b). Table S12. BayesPrism and the ν-SVR core of CIBERSORT alongside the published comparators: (a) donor-held-out accuracy (720 mixtures, 60 donors); (b) per-state Pearson agreement for the two added methods; (c) agreement with measured composition in TCGA-ESCA overall and within each histology (Spearman ρ, bootstrap 95% CI). Table S13. Reference donor-number simulation. (a) Held-out donors of the same study: anchors rebuilt from random subsets of the training donors in each of five folds and applied to the same held-out mixtures. (b) Cross-study transfer: anchors from random subsets of GSE160269 donors applied to 280 GSE154763 myeloid mixtures. Table S14. Transfer to adenocarcinoma. (a) Agreement of each EAC patient's state profile (GSE222078) with the locked ESCC-derived anchors, against held-out ESCC donors; (b) accuracy on 240 EAC pseudo-bulk mixtures; (c) agreement with measured composition in TCGA-ESCA by histology for the locked, histology-matched and pooled references; (d) EAC–ESCC differences (rank-biserial, + = higher in EAC) under each reference. Table S15. Cell states, driver alterations and molecular subtypes in TCGA-ESCA (exploratory, descriptive). (a) Altered patients by histology; (b) EAC–ESCC differences in rank-transformed state fractions before and after adjustment for all 16 alterations; (c) the 20 smallest *p*-values among the 208 state-by-alteration tests (none with FDR < 0.10); (d) ESCC molecular subtypes C1 and C2 (Kruskal–Wallis). Table S16. Sensitivity analyses of the incremental prognostic value of ReCAST features. (a) Locked out-of-fold predictions evaluated with truncated horizons and within subgroups; (b) elastic-net Cox refitted under the locked nested 10 × 5-fold scheme within each cohort, and with features from the histology-matched reference.

## Abbreviations

The following abbreviations are used in this manuscript:

AJCC	American Joint Committee on Cancer
AUROC	Area under the receiver operating characteristic curve
CD4Tconv	Conventional CD4 T cells
CD8Tex	Exhausted CD8 T cells
DC	Dendritic cells
EAC	Esophageal adenocarcinoma
ESCC	Esophageal squamous-cell carcinoma
HPA	Human Protein Atlas
IBS	Integrated Brier score
MAE	Mean absolute error
MC3	Multi-Centre Mutation Calling in Multiple Cancers
Mono/Macro	Monocytes and macrophages
NNLS	Non-negative least squares
ν-SVR	Nu-support vector regression
ReCAST	Reliability-weighted, donor-aware cell-state projection
TCGA-ESCA	The Cancer Genome Atlas esophageal cancer cohort
Tprolif	Proliferating T cells
Treg	Regulatory T cells
UMI	Unique molecular identifier
