# Supplementary material for: ReCAST: Single-Cell-Informed Tumour Microenvironment Profiling for Translational Prognostic Assessment in Esophageal Cancer
Source: Biomedicines. 2026 Oct 9;14(10):2298. doi: 10.3390/biomedicines14102298 (PMC13701590; doi:10.3390/biomedicines14102298)
Supplement: Supplementary file 1 [file biomedicines-14-02298-s001.zip › biomedicines-4601291-supplementary.pdf]

Supplementary Materials

ReCAST: Single-Cell-Informed Tumour Microenvironment Profiling for Translational Prognostic Assessment in Esophageal Cancer

Nahlah Makki Almansour

Department of Biology, College of Science, University of Hafr Al Batin, Hafr Al Batin 31991, Saudi Arabia; nahlama@uhb.edu.sa

Supplementary Figures

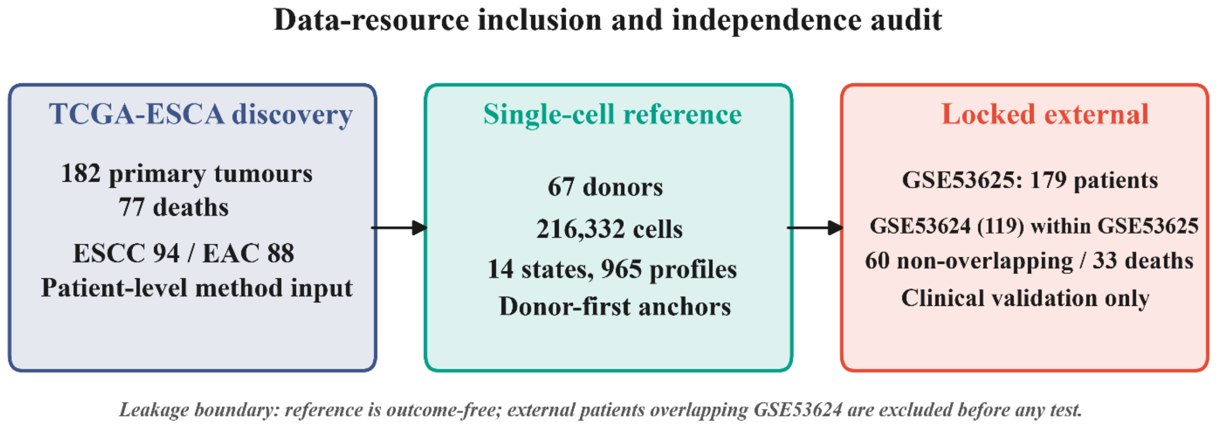

Figure S1. Data-resource inclusion and independence audit: development, reference, and locked external cohorts, with the leakage boundary.

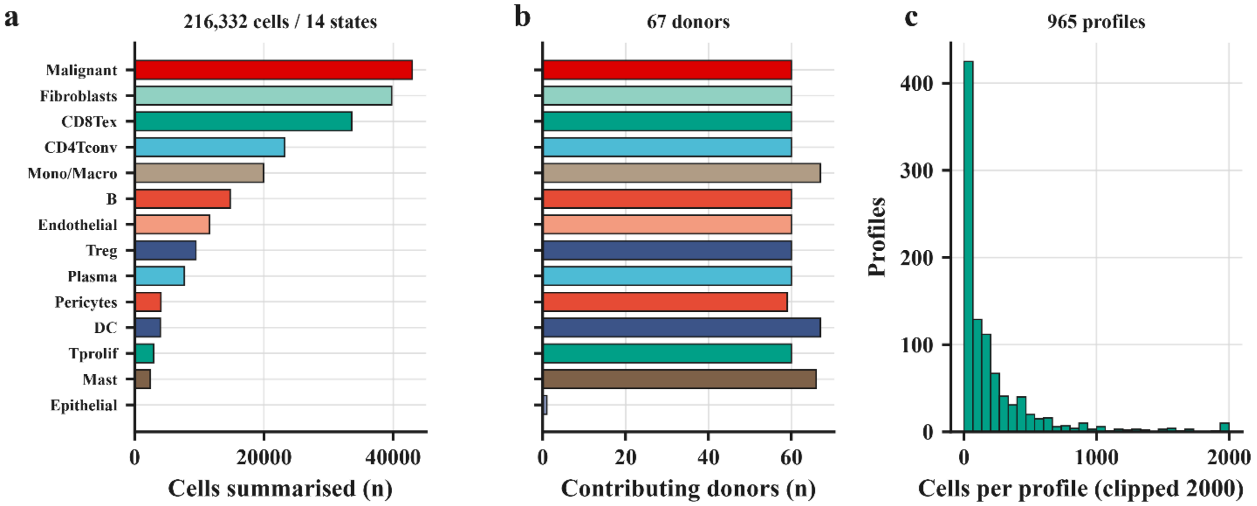

Figure S2. Composition of the donor-aware reference: cells per state, contributing donors per state, and profile-size distribution.

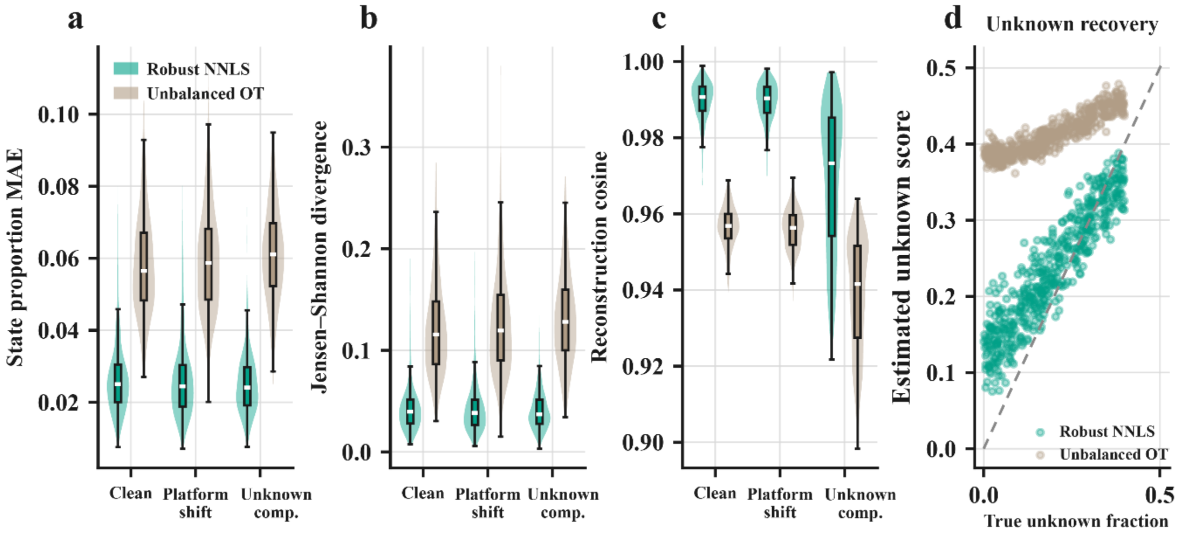

Figure S3. Full simulated-mixture error distributions (500 mixtures per scenario): state MAE, Jensen-Shannon divergence, reconstruction cosine, and unknown-component recovery.

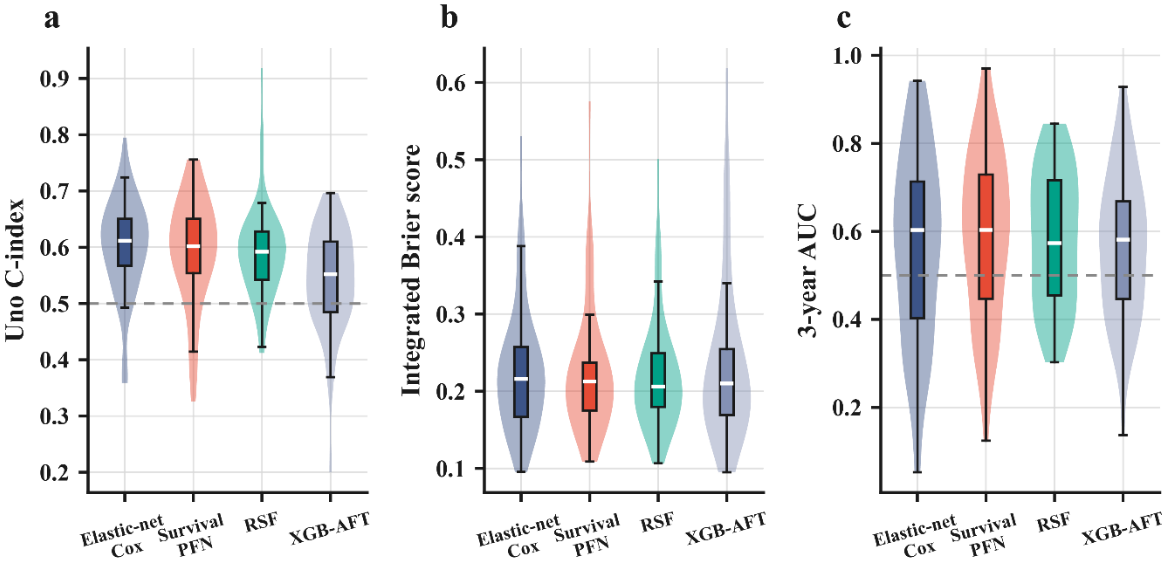

Figure S4. Per-fold internal metric distributions across models on the clinical panel (Uno C, integrated Brier score, 3-year AUC).

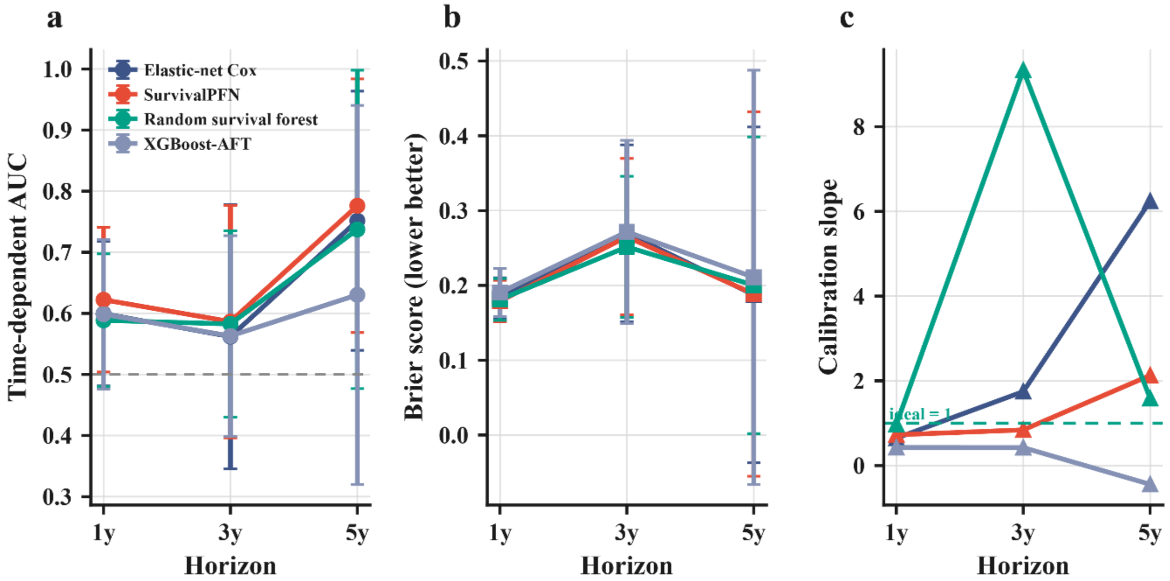

Figure S5. Time-specific discrimination, prediction error, and calibration slope at 1, 3, and 5 years.

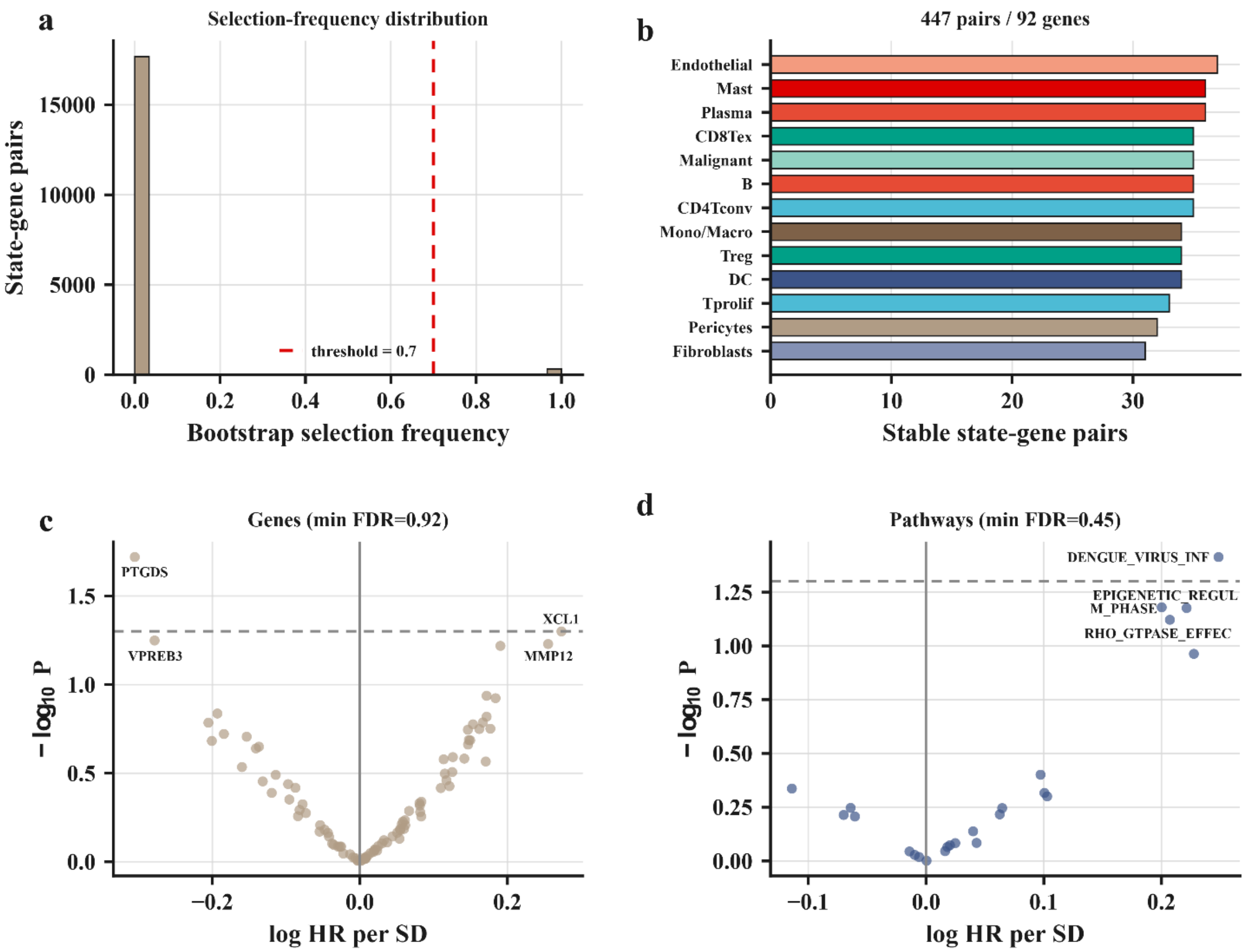

Figure S6. Complete marker-stability and exploratory-association analysis: selection-frequency distribution, stable pairs per state, and gene and pathway volcanoes.

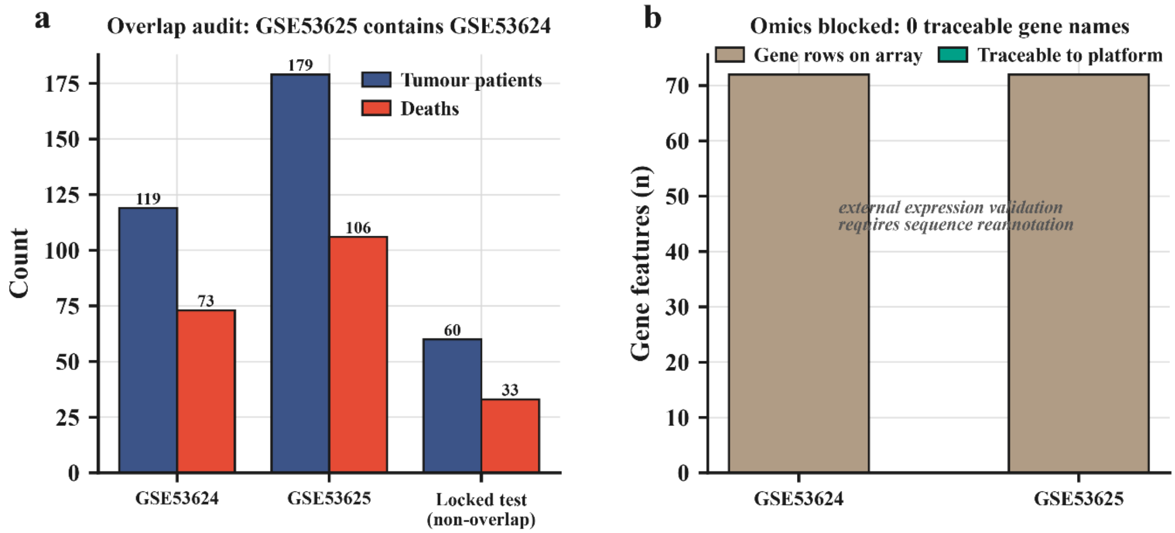

Figure S7. External-cohort overlap audit and platform-annotation traceability.

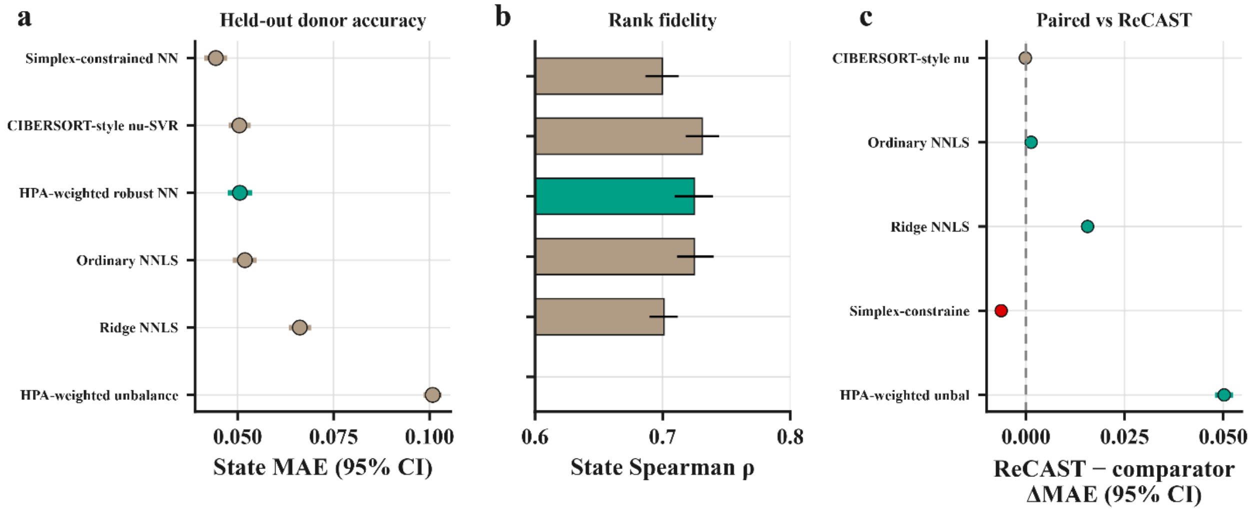

Figure S8. Donor-held-out projection: accuracy and rank fidelity across estimators, with paired comparisons against robust NNLS.

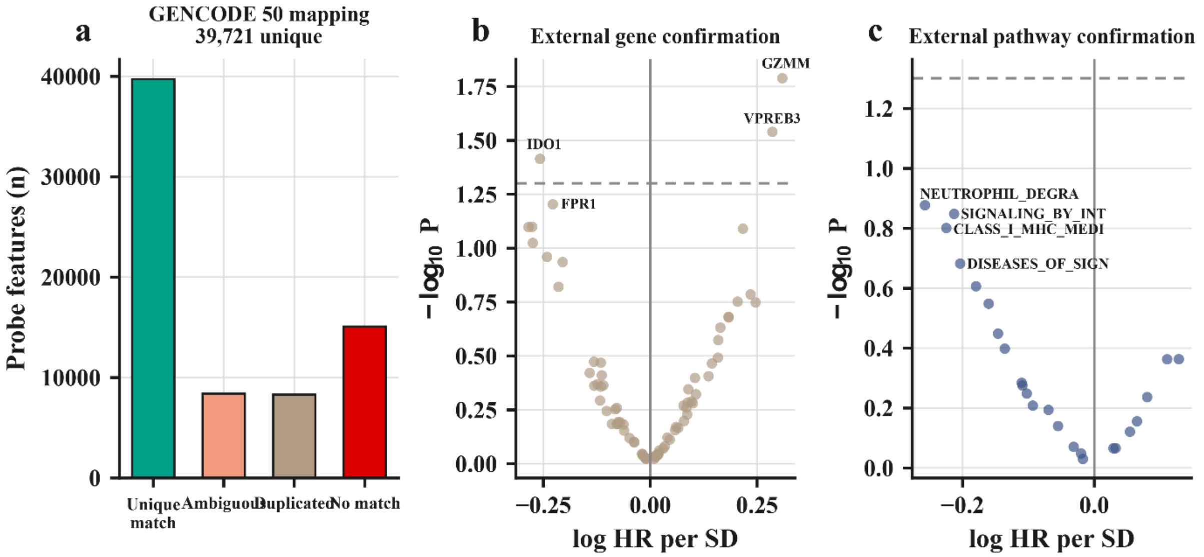

Figure S9. Versioned external molecular reannotation against GENCODE 50 with gene- and pathway-level confirmation.

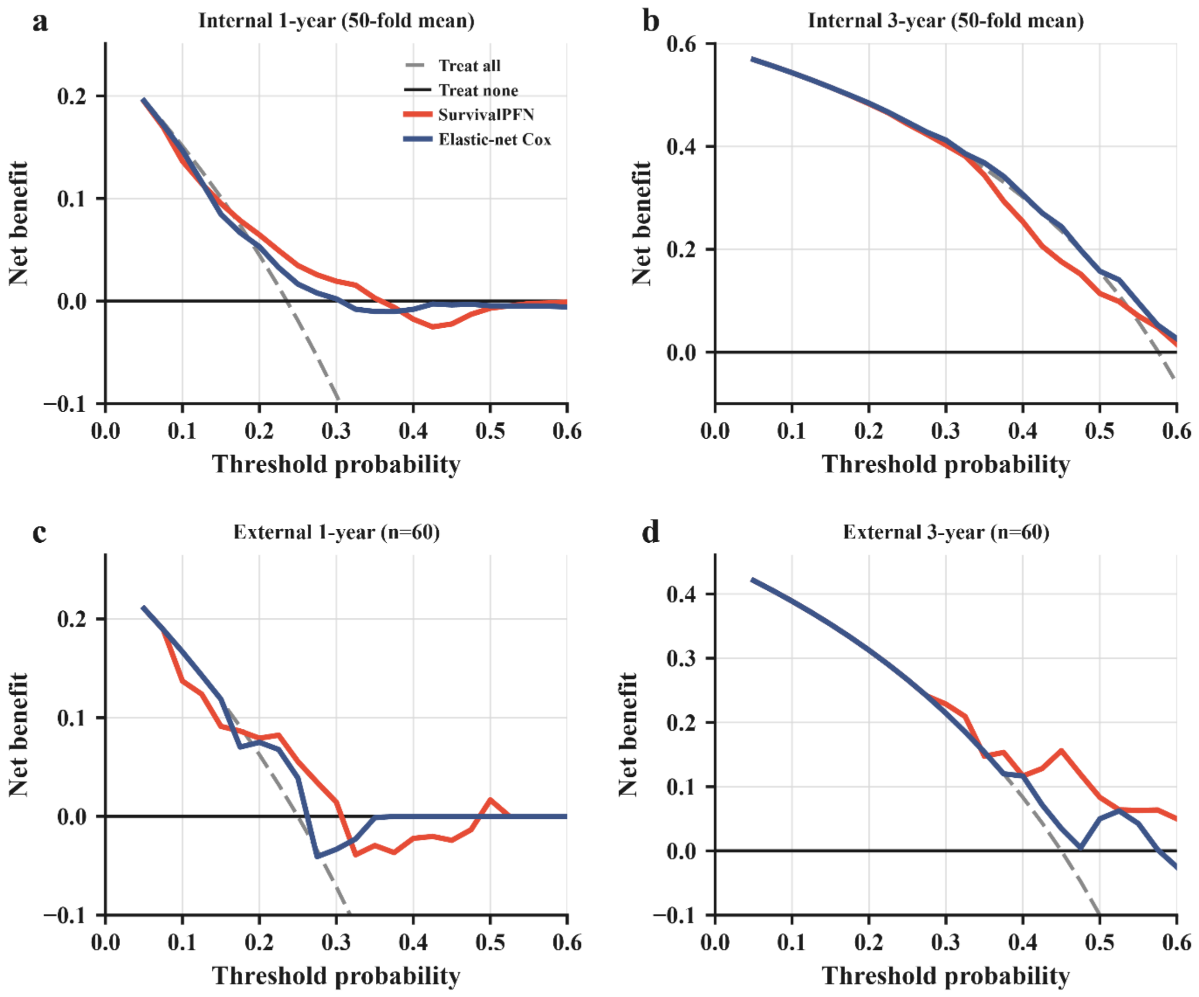

Figure S10. Exploratory IPCW decision-curve analysis, internal (50-fold mean) and external, at 1 and 3 years.

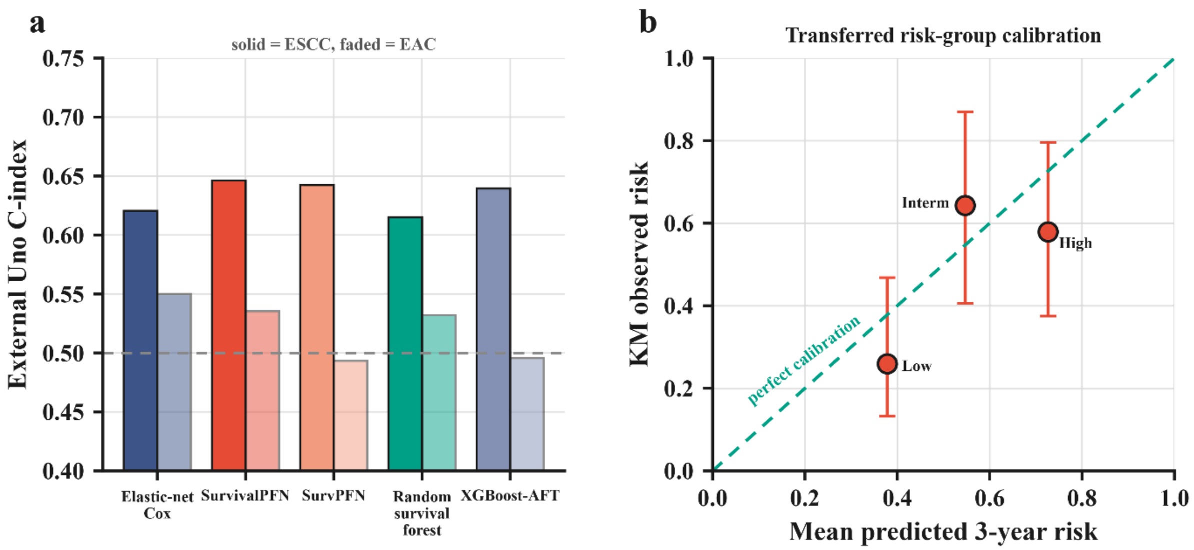

Figure S11. Histology-subgroup discrimination and transferred external risk-group calibration.

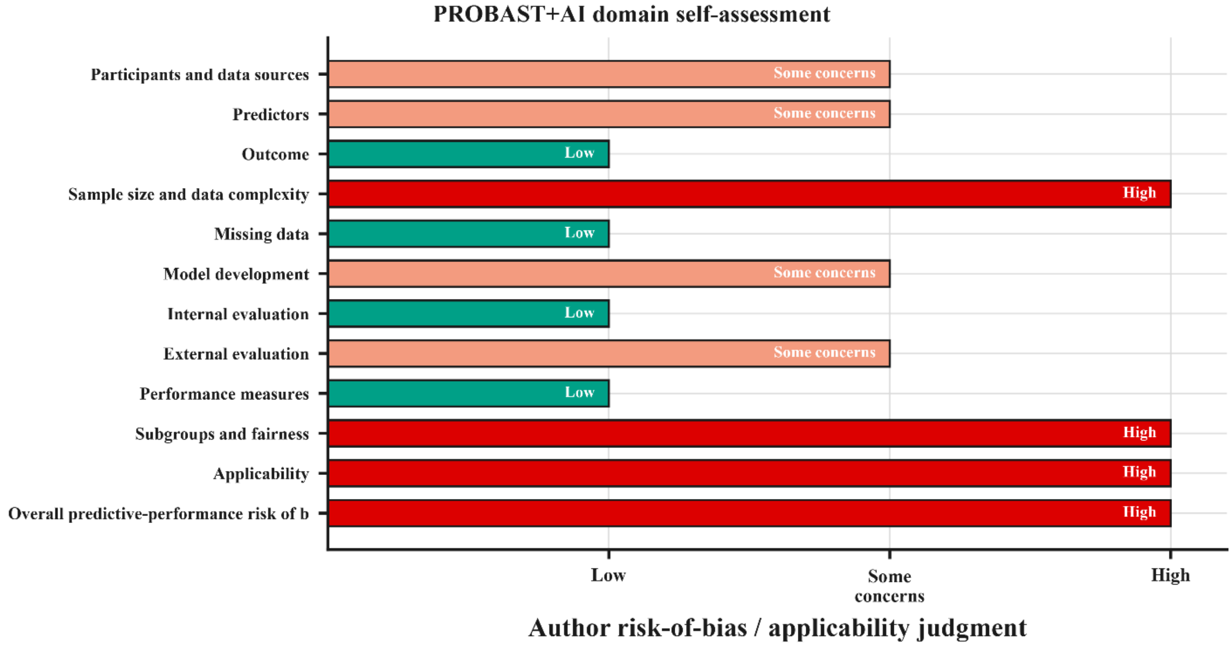

Figure S12. PROBAST+AI author risk-of-bias and applicability self-assessment by domain.

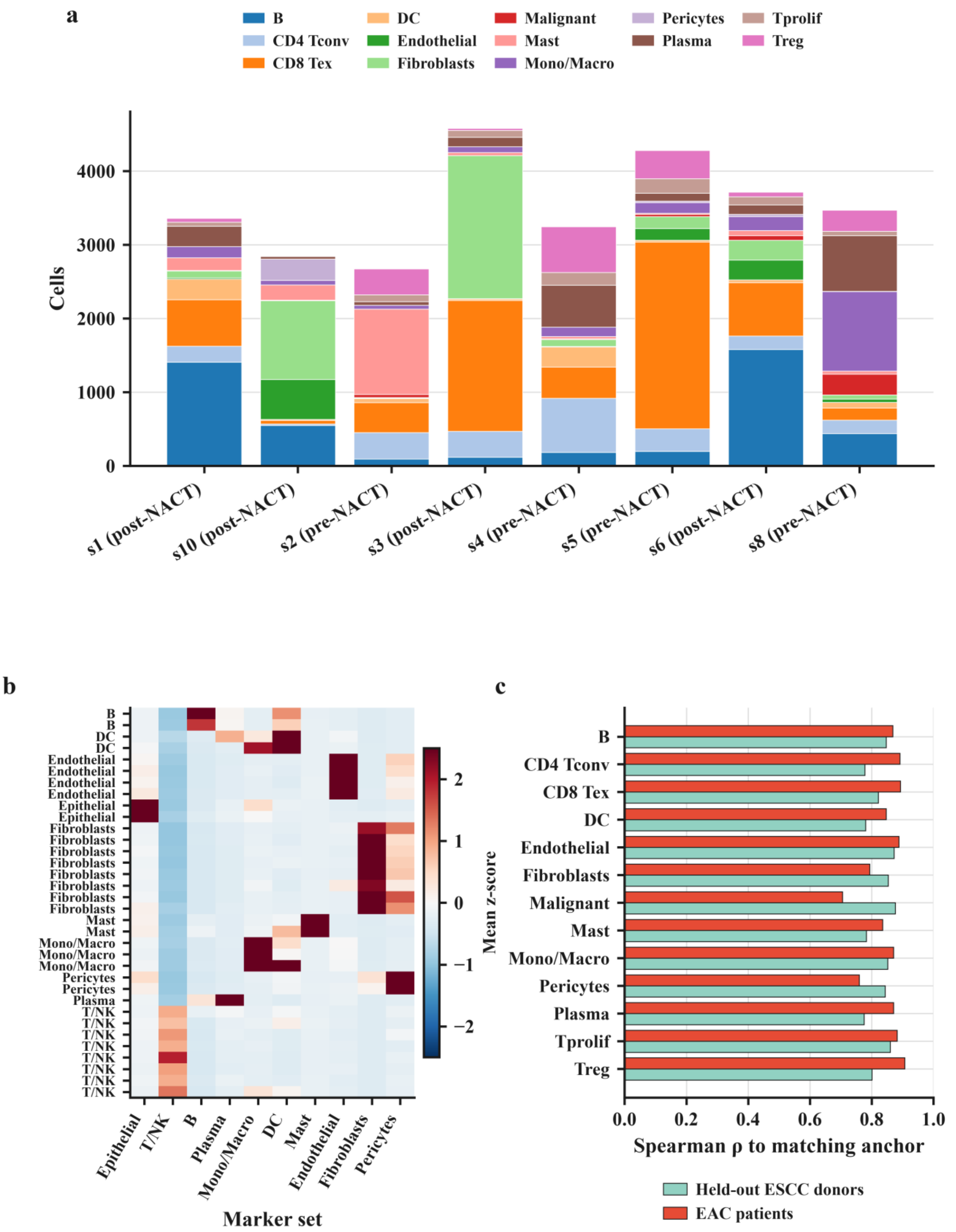

Figure S13. Independent adenocarcinoma single-cell cohort (GSE222078). (a) Cells per state and patient after quality control and marker-based annotation; (b) standardised marker scores of the assigned clusters; (c) agreement of each EAC patient's state profile with the locked ESCC-derived anchors, against held-out ESCC donors.

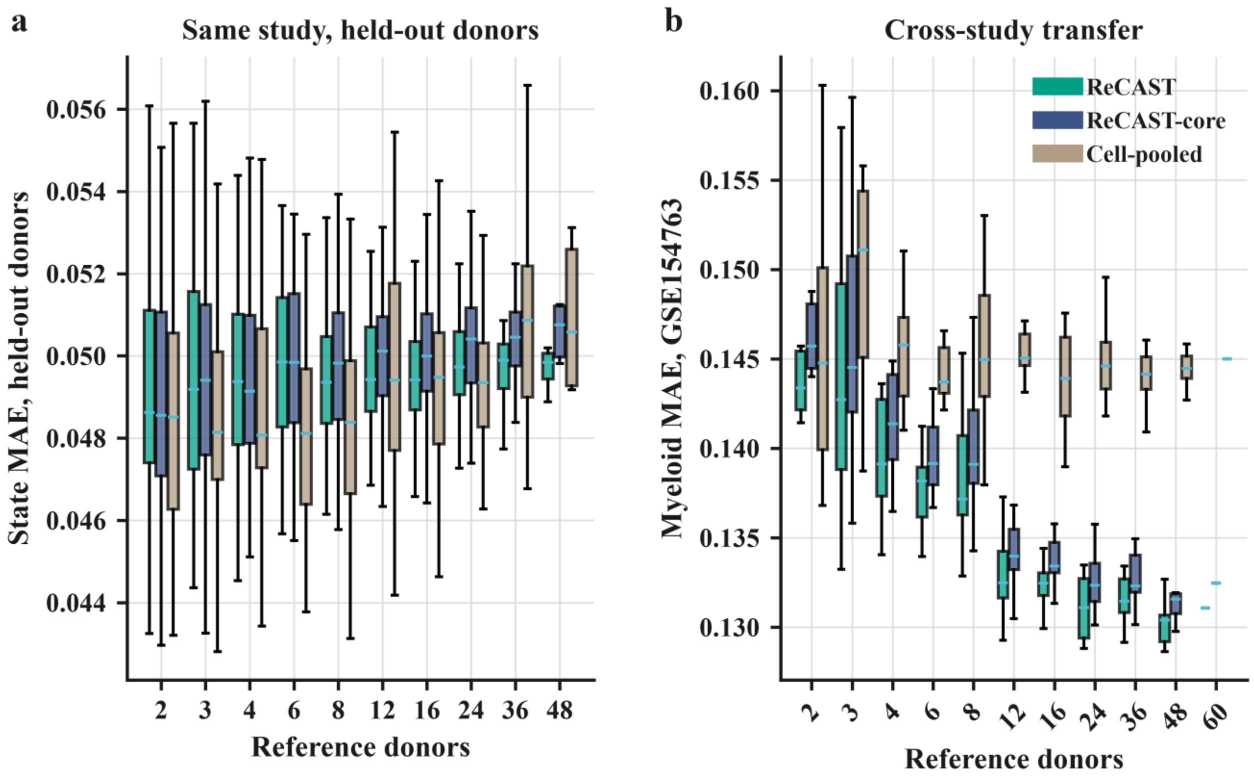

Figure S14. Reference donor-number simulation. Composition error over random draws of reference donors (10 per size) for locked ReCAST (green), the simplified ReCAST-core (blue) and cell-pooled anchors (taupe): (**a**) held-out donors of the same study (five folds); (**b**) transfer of GSE160269 anchors to GSE154763 myeloid mixtures.

Supplementary Tables

Table S1. Clinical-adjusted exploratory survival associations for the 92 prominent marker genes; none passed FDR < 0.05.

Feature	Family	Log hazard ratio per sd	Hazard ratio per sd	Ci95 low	Ci95 high	P value	N	Events	Error	Fdr bh	Valid training splits	Split log hr median	Split log hr q25	Split log hr q75	Direction consistency	Gene	State	State rank	Prominence score	Bootstrap selection frequency	Stable marker	Hpa evidence
PTGDS	gene	-0.3051	0.737	0.5711	0.9512	0.0191	182	77		0.9173	50	-0.3034	-0.3543	-0.2592	1.0	PTGDS	Fibroblasts	7	1.0218	1.0	True	Evidence at protein level
XCL1	gene	0.2733	1.3143	0.9999	1.7277	0.0501	182	77		0.9173	50	0.2618	0.1961	0.3319	1.0	XCL1	CD8Tex	3	1.0089	1.0	True	Evidence at protein level
VPREB3	gene	-0.2783	0.7571	0.5688	1.0076	0.0564	182	77		0.9173	50	-0.2843	-0.344	-0.2211	1.0	VPREB3	B	1	1.0383	1.0	True	Evidence at protein level
MMP12	gene	0.2553	1.2908	0.9903	1.6824	0.059	182	77		0.9173	50	0.2729	0.2209	0.357	1.0	MMP12	Mono/Macro	1	1.0368	1.0	True	Evidence at protein level
C5AR1	gene	0.1906	1.21	0.9916	1.4765	0.0605	182	77		0.9173	50	0.2007	0.1537	0.2516	1.0	C5AR1	Mono/Macro	3	1.0237	1.0	True	Evidence at protein level
CPVL	gene	0.1718	1.1875	0.9586	1.4709	0.1157	182	77		0.9173	50	0.1708	0.1243	0.2096	1.0	CPVL	DC	3	1.0053	1.0	True	Evidence at protein level
IDO1	gene	0.184	1.202	0.9536	1.515	0.1193	182	77		0.9173	50	0.1966	0.136	0.2274	1.0	IDO1	DC	8	0.9512	0.984	True	Evidence at protein level
MAOB	gene	-0.1932	0.8243	0.6355	1.0693	0.1457	182	77		0.9173	50	-0.2142	-0.2496	-0.1564	1.0	MAOB	Mast	9	1.005	1.0	True	Evidence at protein level
TREM2	gene	0.1718	1.1874	0.9388	1.5019	0.1518	182	77		0.9173	50	0.1791	0.1196	0.2305	1.0	TREM2	Mono/Macro	6	1.0114	1.0	True	Evidence at protein level
ICOS	gene	0.1668	1.1815	0.9344	1.494	0.1636	182	77		0.9173	50	0.1523	0.1214	0.2253	1.0	ICOS	Treg	3	0.9761	0.964	True	Evidence at protein level
LGALS7B	gene	-0.2053	0.8144	0.6098	1.0876	0.1642	182	77		0.9173	50	-0.1998	-0.2531	-0.1347	0.98	LGALS7B	Malignant	10	1.0187	1.0	True	Evidence at protein level
MS4A7	gene	0.1533	1.1657	0.9376	1.4494	0.1675	182	77		0.9173	50	0.1595	0.1001	0.2045	1.0	MS4A7	DC	6	0.9623	0.996	True	Evidence at protein level
CFP	gene	0.177	1.1936	0.9229	1.5436	0.1774	182	77		0.9173	50	0.1683	0.1056	0.2219	1.0	CFP	DC	5	0.9737	1.0	True	Evidence at protein level
CD5	gene	0.1619	1.1757	0.9289	1.4882	0.1782	182	77		0.9173	50	0.152	0.1281	0.1991	1.0	CD5	CD4Tconv	6	0.9613	1.0	True	Evidence at protein level
CALD1	gene	0.1464	1.1577	0.9347	1.434	0.1799	182	77		0.9173	50	0.1401	0.1043	0.1791	0.98	CALD1	Pericytes	7	1.0394	1.0	True	Evidence at protein level
TNFRSF13C	gene	-0.1843	0.8317	0.6313	1.0956	0.1899	182	77		0.9173	50	-0.2021	-0.2649	-0.1234	0.98	TNFRSF13C	B	6	0.9597	1.0	True	Evidence at protein level
BLNK	gene	-0.1534	0.8577	0.6794	1.0829	0.1969	182	77		0.9173	50	-0.1558	-0.1901	-0.1081	0.98	BLNK	B	8	0.9296	1.0	True	Evidence at protein level
GZMA	gene	0.1478	1.1593	0.9222	1.4573	0.2055	182	77		0.9173	50	0.161	0.0966	0.1937	0.98	GZMA	CD8Tex	4	1.0062	1.0	True	Evidence at protein level

Feature	Family	Log hazard ratio per sd	Hazard ratio per sd	Ci95 low	Ci95 high	P value	N	Events	Error	Fdr bh	Valid training splits	Split log hr median	Split log hr q25	Split log hr q75	Direction consistency	Gene	State	State rank	Prominence score	Bootstrap selection frequency	Stable marker	Hpa evidence
CD96	gene	0.1496	1.1614	0.9209	1.4646	0.2063	182	77		0.9173	50	0.1532	0.1037	0.197	1.0	CD96	CD8Tex	8	0.9651	1.0	True	Evidence at protein level
LY6D	gene	-0.2007	0.8182	0.5986	1.1181	0.2079	182	77		0.9173	50	-0.2078	-0.2816	-0.123	0.86	LY6D	Malignant	4	1.041	1.0	True	Evidence at protein level
TREM1	gene	0.1467	1.158	0.917	1.4624	0.2179	182	77		0.9173	50	0.1592	0.108	0.2023	0.96	TREM1	Mono/Macro	9	0.9989	1.0	True	Evidence at protein level
GATA2	gene	-0.1369	0.8721	0.6994	1.0874	0.2241	182	77		0.9173	50	-0.1377	-0.1724	-0.0861	1.0	GATA2	Mast	2	1.0502	1.0	True	Evidence at protein level
CCL19	gene	-0.1409	0.8686	0.6904	1.0929	0.2293	182	77		0.9173	50	-0.127	-0.1787	-0.1004	1.0	CCL19	DC	1	1.0254	1.0	True	Evidence at protein level
PLA2G7	gene	0.1261	1.1344	0.9123	1.4105	0.2566	182	77		0.9259	50	0.1419	0.0844	0.1807	1.0	PLA2G7	Mono/Macro	8	0.9999	1.0	True	Evidence at protein level
CD247	gene	0.1416	1.1521	0.8997	1.4754	0.2617	182	77		0.9259	50	0.1445	0.0929	0.1934	0.96	CD247	CD8Tex	7	0.9776	1.0	True	Evidence at protein level
PILRA	gene	0.1136	1.1203	0.9178	1.3675	0.2642	182	77		0.9259	50	0.1236	0.0714	0.1631	0.98	PILRA	Mono/Macro	10	0.9966	1.0	True	Evidence at protein level
PLVAP	gene	0.1707	1.1862	0.8748	1.6083	0.2717	182	77		0.9259	50	0.1713	0.1261	0.2043	0.96	PLVAP	Endothelial	3	1.0454	1.0	True	Evidence at protein level
KRT15	gene	-0.1598	0.8523	0.6331	1.1474	0.292	182	77		0.9541	50	-0.1712	-0.2203	-0.0837	0.86	KRT15	Malignant	1	1.0488	1.0	True	Evidence at protein level
ITK	gene	0.1253	1.1335	0.8892	1.4449	0.3116	182	77		0.9541	50	0.1102	0.0749	0.184	1.0	ITK	CD4Tconv	4	0.9673	1.0	True	Evidence at protein level
HCK	gene	0.1153	1.1222	0.8949	1.4071	0.3181	182	77		0.9541	50	0.1249	0.0765	0.1701	0.96	HCK	DC	7	0.9546	0.99	True	Evidence at protein level
SLPI	gene	-0.1141	0.8922	0.7115	1.1188	0.3232	182	77		0.9541	50	-0.1138	-0.1548	-0.0491	0.94	SLPI	Malignant	6	1.0306	1.0	True	Evidence at protein level
CD6	gene	0.1173	1.1245	0.8806	1.4359	0.3469	182	77		0.9541	50	0.1042	0.0736	0.1815	0.96	CD6	CD4Tconv	7	0.9578	1.0	True	Evidence at protein level
CD19	gene	-0.1316	0.8767	0.6645	1.1566	0.3518	182	77		0.9541	50	-0.1416	-0.2032	-0.0756	1.0	CD19	B	2	1.0283	1.0	True	Evidence at protein level
MGP	gene	-0.0972	0.9073	0.7354	1.1195	0.3644	182	77		0.9541	50	-0.1045	-0.1283	-0.081	0.98	MGP	Pericytes	5	1.05	1.0	True	Evidence at protein level
DNAAF1	gene	0.1215	1.1292	0.8636	1.4764	0.3746	182	77		0.9541	50	0.1219	0.0876	0.17	0.94	DNAAF1	Plasma	3	1.0325	1.0	True	Evidence at protein level
RAB27B	gene	-0.0873	0.9164	0.7533	1.1148	0.3824	182	77		0.9541	50	-0.072	-0.135	-0.0183	0.86	RAB27B	Mast	10	0.9987	0.996	True	Evidence at protein level
S100B	gene	0.1098	1.116	0.8718	1.4287	0.3837	182	77		0.9541	50	0.1177	0.0647	0.1737	0.9	S100B	DC	4	0.995	1.0	True	Evidence at protein level
KRT6B	gene	-0.1195	0.8874	0.6686	1.1779	0.4083	182	77		0.9865	50	-0.1182	-0.1792	-0.0543	0.84	KRT6B	Malignant	5	1.0312	1.0	True	Evidence at protein level
KIT	gene	-0.0956	0.9088	0.7109	1.1618	0.4453	182	77		0.9865	50	-0.0845	-0.1305	-0.053	0.94	KIT	Mast	5	1.0238	1.0	True	Evidence at

Feature	Family	Log hazard ratio per sd	Hazard ratio per sd	Ci95 low	Ci95 high	P value	N	Events	Error	Fdr bh	Valid training splits	Split log hr median	Split log hr q25	Split log hr q75	Direction consistency	Gene	State	State rank	Prominence score	Bootstrap selection frequency	Stable marker	Hpa evidence
																						protein level
CSF2RA	gene	0.0833	1.0869	0.8721	1.3546	0.4582	182	77		0.9865	50	0.0905	0.0468	0.1388	0.92	CSF2RA	DC	2	1.0236	1.0	True	Evidence at protein level
MMP19	gene	0.0807	1.0841	0.8718	1.348	0.4677	182	77		0.9865	50	0.0778	0.048	0.128	0.9	MMP19	Mono/Macro	5	1.014	1.0	True	Evidence at protein level
NTRK1	gene	-0.0776	0.9253	0.7483	1.1442	0.4735	182	77		0.9865	50	-0.0826	-0.1159	-0.0461	0.88	NTRK1	Mast	7	1.013	1.0	True	Evidence at protein level
IGHV3-7	gene	0.0814	1.0848	0.8648	1.3608	0.4815	182	77		0.9865	50	0.0756	0.0299	0.1296	0.9	IGHV3-7	Plasma	2	1.0367	1.0	True	Evidence at protein level
GZMK	gene	-0.0818	0.9215	0.7225	1.1751	0.5098	182	77		0.9865	50	-0.0772	-0.119	-0.0437	0.88	GZMK	CD8Tex	2	1.0191	1.0	True	Evidence at protein level
LUM	gene	0.0668	1.069	0.8737	1.308	0.5166	182	77		0.9865	50	0.0572	0.0186	0.1062	0.84	LUM	Pericytes	8	1.0377	1.0	True	Evidence at protein level
COL4A2	gene	0.0818	1.0852	0.8438	1.3957	0.5242	182	77		0.9865	50	0.0849	0.047	0.1283	0.94	COL4A2	Pericytes	9	1.03	1.0	True	Evidence at protein level
CD22	gene	-0.0732	0.9295	0.7392	1.1686	0.5312	182	77		0.9865	50	-0.069	-0.1238	-0.018	0.84	CD22	B	4	0.9725	1.0	True	Evidence at protein level
GPX2	gene	-0.0839	0.9195	0.6967	1.2136	0.5533	182	77		0.9865	50	-0.0658	-0.1253	-0.0303	0.88	GPX2	Malignant	7	1.0274	1.0	True	Evidence at protein level
IL1RL1	gene	0.0832	1.0868	0.8244	1.4326	0.555	182	77		0.9865	50	0.0861	0.0435	0.1286	0.88	IL1RL1	Mast	4	1.0436	1.0	True	Evidence at protein level
IGFBP5	gene	0.0611	1.0631	0.8545	1.3225	0.5831	182	77		0.9865	50	0.0567	0.0139	0.0987	0.8	IGFBP5	Endothelial	7	1.026	1.0	True	Evidence at protein level
CSF1R	gene	0.0583	1.06	0.8555	1.3134	0.5941	182	77		0.9865	50	0.0607	0.0145	0.1075	0.86	CSF1R	Mono/Macro	4	1.0181	1.0	True	Evidence at protein level
IGLV6-57	gene	0.0569	1.0586	0.8488	1.3201	0.6135	182	77		0.9865	50	0.0534	0.0068	0.0912	0.8	IGLV6-57	Plasma	7	1.0094	1.0	True	Evidence at protein level
RARRES2	gene	-0.0538	0.9476	0.7655	1.1731	0.6212	182	77		0.9865	50	-0.0642	-0.0912	-0.0166	0.82	RARRES2	Fibroblasts	6	1.0266	1.0	True	Evidence at protein level
GZMM	gene	0.0615	1.0635	0.8327	1.3582	0.622	182	77		0.9865	50	0.0645	0.0159	0.1032	0.82	GZMM	CD4Tconv	2	0.9857	1.0	True	Evidence at protein level
JSRP1	gene	0.0599	1.0618	0.8182	1.3778	0.6522	182	77		0.9865	50	0.0653	-0.0109	0.1212	0.68	JSRP1	Plasma	5	1.0288	1.0	True	Evidence at protein level
ZAP70	gene	0.0545	1.056	0.8318	1.3407	0.6543	182	77		0.9865	50	0.0458	0.0038	0.1122	0.78	ZAP70	CD4Tconv	10	0.9506	1.0	True	Evidence at protein level
GNG11	gene	-0.0477	0.9534	0.771	1.179	0.6599	182	77		0.9865	50	-0.0598	-0.0832	0.0015	0.74	GNG11	Endothelial	10	1.017	1.0	True	Evidence at protein level
MEF2B	gene	0.0546	1.0562	0.8257	1.351	0.6636	182	77		0.9865	50	0.0489	0.0085	0.1022	0.8	MEF2B	Plasma	9	0.9871	1.0	True	Evidence at protein level
FXYD3	gene	-0.0546	0.9468	0.7328	1.2234	0.6761	182	77		0.9865	50	-0.0439	-0.1189	0.0115	0.7	FXYD3	Malignant	9	1.0249	1.0	True	Evidence at protein level

Feature	Family	Log hazard ratio per sd	Hazard ratio per sd	Ci95 low	Ci95 high	P value	N	Events	Error	Fdr bh	Valid training splits	Split log hr median	Split log hr q25	Split log hr q75	Direction consistency	Gene	State	State rank	Prominence score	Bootstrap selection frequency	Stable marker	Hpa evidence
CTLA4	gene	0.0507	1.052	0.8232	1.3444	0.6852	182	77		0.9865	50	0.0429	0.0084	0.0888	0.8	CTLA4	Treg	1	0.9958	1.0	True	Evidence at protein level
KLRD1	gene	-0.0433	0.9576	0.7757	1.1823	0.6871	182	77		0.9865	50	-0.0379	-0.0947	-0.004	0.78	KLRD1	CD8Tex	5	1.0055	1.0	True	Evidence at protein level
CD8B	gene	0.0442	1.0452	0.8212	1.3303	0.7193	182	77		0.9865	50	0.0474	-0.0061	0.0948	0.72	CD8B	CD8Tex	1	1.0197	1.0	True	Evidence at protein level
TPSAB1	gene	-0.0418	0.959	0.7623	1.2066	0.721	182	77		0.9865	50	-0.0498	-0.0713	0.0077	0.68	TPSAB1	Mast	1	1.0553	1.0	True	Evidence at protein level
RASSF6	gene	0.0537	1.0552	0.7671	1.4515	0.7413	182	77		0.9865	50	0.0339	-0.0263	0.1023	0.58	RASSF6	Plasma	8	1.0045	1.0	True	Evidence at protein level
COL3A1	gene	0.0326	1.0331	0.8413	1.2686	0.756	182	77		0.9865	50	0.0368	-0.0127	0.0696	0.68	COL3A1	Pericytes	4	1.0503	1.0	True	Evidence at protein level
CD24	gene	0.0369	1.0376	0.8032	1.3404	0.7777	182	77		0.9865	50	0.0368	-0.0086	0.1082	0.7	CD24	B	7	0.9543	1.0	True	Evidence at protein level
BTK	gene	0.03	1.0304	0.829	1.2808	0.7869	182	77		0.9865	50	0.0167	-0.0207	0.078	0.62	BTK	Mast	8	1.0083	0.998	True	Evidence at protein level
S100A7	gene	-0.0375	0.9632	0.7314	1.2684	0.7895	182	77		0.9865	50	-0.0476	-0.0847	0.0147	0.66	S100A7	Malignant	3	1.0439	1.0	True	Evidence at protein level
RGS5	gene	-0.0353	0.9653	0.7317	1.2733	0.8025	182	77		0.9865	50	-0.0381	-0.0797	0.0033	0.7	RGS5	Pericytes	6	1.044	1.0	True	Evidence at protein level
COL1A2	gene	0.0248	1.0251	0.8303	1.2657	0.8174	182	77		0.9865	50	0.0262	-0.022	0.0625	0.68	COL1A2	Pericytes	1	1.0512	1.0	True	Evidence at protein level
IGHG4	gene	-0.0254	0.9749	0.7827	1.2143	0.8205	182	77		0.9865	50	-0.0313	-0.0634	0.0264	0.64	IGHG4	Plasma	4	1.0305	1.0	True	Evidence at protein level
SLC18A2	gene	-0.0296	0.9709	0.7517	1.254	0.8208	182	77		0.9865	50	-0.0311	-0.0768	0.0097	0.7	SLC18A2	Mast	3	1.0485	1.0	True	Evidence at protein level
COL15A1	gene	-0.0269	0.9734	0.7706	1.2295	0.8211	182	77		0.9865	50	-0.0183	-0.0501	0.0106	0.72	COL15A1	Endothelial	4	1.0436	1.0	True	Evidence at protein level
CTHRC1	gene	0.0206	1.0208	0.825	1.263	0.8498	182	77		0.9865	50	0.0082	-0.0349	0.0644	0.62	CTHRC1	Fibroblasts	9	1.0152	1.0	True	Evidence at protein level
SPIB	gene	0.0234	1.0237	0.7825	1.3392	0.8643	182	77		0.9865	50	0.0166	-0.0258	0.073	0.56	SPIB	B	5	0.9677	1.0	True	Evidence at protein level
IGLL5	gene	0.0196	1.0198	0.8141	1.2775	0.8644	182	77		0.9865	50	0.0026	-0.0299	0.0526	0.52	IGLL5	Plasma	6	1.0103	1.0	True	Evidence at protein level
OLR1	gene	0.0182	1.0183	0.8041	1.2897	0.8801	182	77		0.9865	50	0.0239	-0.0215	0.0709	0.64	OLR1	Mono/Macro	2	1.0251	1.0	True	Evidence at protein level
IGHV4-34	gene	0.0143	1.0145	0.8223	1.2516	0.8935	182	77		0.9865	50	0.0123	-0.0268	0.0562	0.56	IGHV4-34	Plasma	1	1.042	1.0	True	Evidence at protein level
CALML3	gene	-0.0224	0.9779	0.6913	1.3832	0.8994	182	77		0.9865	50	-0.0257	-0.0652	0.0821	0.62	CALML3	Malignant	8	1.0272	1.0	True	Evidence at protein level
FPR1	gene	-0.0132	0.9869	0.789	1.2343	0.9077	182	77		0.9865	50	0.0013	-0.0609	0.0415	0.48	FPR1	Mono/Macro	7	1.002	1.0	True	Evidence at

Feature	Family	Log hazard ratio per sd	Hazard ratio per sd	Ci95 low	Ci95 high	P value	N	Events	Error	Fdr bh	Valid training splits	Split log hr median	Split log hr q25	Split log hr q75	Direction consistency	Gene	State	State rank	Prominence score	Bootstrap selection frequency	Stable marker	Hpa evidence
																						protein level
BGN	gene	0.01	1.01	0.8098	1.2598	0.9295	182	77		0.9865	50	0.0015	-0.0366	0.0569	0.56	BGN	Pericytes	3	1.0508	1.0	True	Evidence at protein level
BANK1	gene	-0.0091	0.991	0.7666	1.2811	0.9449	182	77		0.9865	50	-0.0056	-0.051	0.0216	0.54	BANK1	B	3	0.9995	1.0	True	Evidence at protein level
VMO1	gene	0.0058	1.0058	0.8412	1.2027	0.9495	182	77		0.9865	50	0.0054	-0.0333	0.0385	0.54	VMO1	DC	9	0.9493	1.0	True	Evidence at protein level
FCGR2B	gene	0.0078	1.0078	0.7841	1.2954	0.9514	182	77		0.9865	50	0.0203	-0.0317	0.0684	0.54	FCGR2B	Plasma	10	0.9821	1.0	True	Evidence at protein level
RAMP2	gene	0.0079	1.008	0.7798	1.3028	0.9517	182	77		0.9865	50	0.0118	-0.047	0.0568	0.56	RAMP2	Endothelial	5	1.0411	1.0	True	Evidence at protein level
CNRIP1	gene	-0.0059	0.9941	0.8113	1.2182	0.9545	182	77		0.9865	50	-0.0076	-0.0381	0.0252	0.54	CNRIP1	Mast	6	1.019	1.0	True	Evidence at protein level
TM4SF1	gene	0.0066	1.0066	0.7749	1.3076	0.9607	182	77		0.9865	50	0.0103	-0.0408	0.055	0.58	TM4SF1	Endothelial	9	1.0171	0.982	True	Evidence at protein level
ESAM	gene	0.0077	1.0077	0.7334	1.3846	0.9623	182	77		0.9865	50	0.0144	-0.0382	0.0923	0.56	ESAM	Endothelial	8	1.0218	1.0	True	Evidence at protein level
SPARCL1	gene	0.0027	1.0027	0.8072	1.2455	0.9805	182	77		0.9865	50	0.0049	-0.0379	0.0302	0.54	SPARCL1	Pericytes	2	1.051	1.0	True	Evidence at protein level
PKMYT1	gene	-0.0029	0.9971	0.7801	1.2744	0.9813	182	77		0.9865	50	0.0164	-0.0345	0.0472	0.46	PKMYT1	Tprolif	8	0.9318	0.986	True	Evidence at protein level
DCN	gene	0.0016	1.0016	0.8301	1.2086	0.9865	182	77		0.9865	50	-0.0064	-0.0351	0.0407	0.44	DCN	Fibroblasts	5	1.0268	1.0	True	Evidence at protein level
CLDN4	gene	-0.0029	0.9971	0.7104	1.3994	0.9865	182	77		0.9865	50	0.0068	-0.0565	0.0702	0.48	CLDN4	Malignant	2	1.0457	1.0	True	Evidence at protein level

Table S2. Clinical-adjusted exploratory survival associations for the 24 prespecified pathways; none passed FDR < 0.05.

Feature	Family	Log hazard ratio per sd	Hazard ratio per sd	Ci95 low	Ci95 high	P value	N	Events	Error	Fdr bh	Valid training splits	Split log hr median	Split log hr q25	Split log hr q75	Direction consistency
pathway__REACTOME_DENGUE_VIRUS_INFECTION	pathway	0.2485	1.282	1.0131	1.6224	0.0386	182	77		0.4536	50	0.2417	0.2043	0.3029	1.0
pathway__REACTOME_M_PHASE	pathway	0.2002	1.2216	0.9867	1.5125	0.0662	182	77		0.4536	50	0.205	0.1572	0.2503	1.0
pathway__REACTOME_EPIGENETIC_REGULATION_OF_GENE_EXPRESSION	pathway	0.2213	1.2477	0.9849	1.5807	0.0667	182	77		0.4536	50	0.2186	0.1832	0.2794	1.0
pathway__REACTOME_RHO_GTPASE_EFFECTORS	pathway	0.2071	1.2301	0.9789	1.5458	0.0756	182	77		0.4536	50	0.211	0.1658	0.251	1.0
pathway__REACTOME_CELL_CELL_COMMUNICATION	pathway	0.2275	1.2555	0.9506	1.658	0.1089	182	77		0.5226	50	0.2288	0.1833	0.2656	1.0
pathway__REACTOME_DNA_REPAIR	pathway	0.0973	1.1022	0.88	1.3805	0.3971	182	77		0.9974	50	0.1012	0.0543	0.1379	0.98
pathway__REACTOME_METABOLISM_OF_AMINO_ACIDS_AND_DERIVATIVES	pathway	-0.1139	0.8923	0.6593	1.2078	0.4607	182	77		0.9974	50	-0.1045	-0.1755	-0.0504	0.94
pathway__REACTOME_CLASS_I_MHC_MEDIATED_ANTIGEN_PROCESSING_PRESENTATION	pathway	0.1006	1.1058	0.8351	1.4643	0.4827	182	77		0.9974	50	0.1136	0.0714	0.1556	0.92

Feature	Family	Log hazard ratio per sd	Hazard ratio per sd	Ci95 low	Ci95 high	P value	N	Events	Error	Fdr bh	Valid training splits	Split log hr median	Split log hr q25	Split log hr q75	Direction consistency
pathway__REACTOME_ASPARAGINE_N_LINKED_GLYCOSYLATION	pathway	0.1029	1.1084	0.8214	1.4956	0.5008	182	77		0.9974	50	0.1081	0.0461	0.1745	0.86
pathway__REACTOME_RHO_GTPASE_CYCLE	pathway	-0.064	0.938	0.7536	1.1675	0.5665	182	77		0.9974	50	-0.0638	-0.107	-0.0337	0.94
pathway__REACTOME_TRANSCRIPTIONAL_REGULATION_BY_TP53	pathway	0.0647	1.0668	0.8545	1.332	0.5677	182	77		0.9974	50	0.0733	0.0284	0.1155	0.88
pathway__REACTOME_SIGNALING_BY_INTERLEUKINS	pathway	0.0626	1.0646	0.8386	1.3516	0.6072	182	77		0.9974	50	0.0608	0.0213	0.1313	0.86
pathway__REACTOME_OLFACTORY_SIGNALING_PATHWAY	pathway	-0.07	0.9324	0.7121	1.221	0.611	182	77		0.9974	50	-0.0887	-0.1257	-0.0459	0.8
pathway__REACTOME_NEURONAL_SYSTEM	pathway	-0.0604	0.9414	0.741	1.196	0.6212	182	77		0.9974	50	-0.0583	-0.1132	-0.0293	0.88
pathway__REACTOME_G_ALPHA_I_SIGNALLING_EVENTS	pathway	0.04	1.0408	0.8309	1.3038	0.7278	182	77		0.9974	50	0.0342	0.0043	0.0879	0.8
pathway__REACTOME_SIGNALING_BY_WNT	pathway	0.043	1.0439	0.7147	1.5248	0.8241	182	77		0.9974	50	0.0296	-0.0194	0.0855	0.58
pathway__REACTOME_SARS_COV_INFECTIONS	pathway	0.0249	1.0252	0.8215	1.2795	0.8258	182	77		0.9974	50	0.0102	-0.021	0.0812	0.62
pathway__REACTOME_GPCR_LIGAND_BINDING	pathway	0.0203	1.0206	0.8324	1.2513	0.8449	182	77		0.9974	50	0.0198	-0.0134	0.0663	0.6
pathway__REACTOME_TRANSLATION	pathway	0.0182	1.0183	0.8309	1.2481	0.861	182	77		0.9974	50	0.0146	-0.017	0.056	0.66
pathway__REACTOME_NEUTROPHIL_DEGRANULATION	pathway	0.0162	1.0164	0.7884	1.3103	0.9003	182	77		0.9974	50	0.0279	-0.0436	0.091	0.62
pathway__REACTOME_EXTRACELLULAR_MATRIX_ORGANIZATION	pathway	-0.014	0.9861	0.7864	1.2364	0.9032	182	77		0.9974	50	-0.0237	-0.0564	0.0192	0.6
pathway__REACTOME_DISEASES_OF_SIGNAL_TRANSDUCTION_BY_GROWTH_FACTOR_RECEPTORS_AND_SECOND_MESSENGERS	pathway	-0.0096	0.9905	0.7799	1.2578	0.9374	182	77		0.9974	50	-0.0075	-0.049	0.0336	0.52
pathway__REACTOME_CLASS_A_1_RHODOPSIN_LIKE_RECEPTORS	pathway	-0.0059	0.9941	0.7967	1.2404	0.9582	182	77		0.9974	50	-0.0111	-0.0415	0.0351	0.52
pathway__REACTOME_MAPK_FAMILY_SIGNALING_CASCADES	pathway	0.0005	1.0005	0.7633	1.3113	0.9974	182	77		0.9974	50	0.0083	-0.0254	0.0423	0.56

Table S3. Donor-held-out projection validation of the reference estimator.

Scenario	Method	Method label	Donors	Mixtures	State mae mean	State mae ci95 low	State mae ci95 high	Jensen shannon mean	Jensen shannon ci95 low	Jensen shannon ci95 high	State spearman mean	State spearman ci95 low	State spearman ci95 high
heldout_donor_clean	nu_svr	CIBERSORT-style nu-SVR	67	335	0.0504	0.0484	0.0527	0.1138	0.1071	0.1206	0.731	0.7181	0.7442
heldout_donor_clean	ordinary_nnls	Ordinary NNLS	67	335	0.0519	0.0494	0.0543	0.1195	0.1111	0.1272	0.7249	0.7113	0.7399
heldout_donor_clean	ridge_nnls	Ridge NNLS	67	335	0.0662	0.0641	0.0685	0.1739	0.1662	0.181	0.701	0.6899	0.7118
heldout_donor_clean	robust_nnls_hpa	HPA-weighted robust NNLS (ReCAST)	67	335	0.0506	0.0481	0.0531	0.1149	0.1069	0.1228	0.7249	0.7094	0.7396
heldout_donor_clean	simplex_nnls	Simplex-constrained NNLS	67	335	0.0443	0.042	0.0466	0.0942	0.0871	0.101	0.6998	0.6866	0.7125
heldout_donor_clean	unbalanced_ot_hpa	HPA-weighted unbalanced OT	67	335	0.1008	0.0992	0.1024	0.3253	0.318	0.3333	0.4704	0.4442	0.4944
heldout_donor_platform_shift	nu_svr	CIBERSORT-style nu-SVR	67	335	0.0489	0.047	0.0508	0.1093	0.1034	0.115	0.7244	0.7119	0.7364
heldout_donor_platform_shift	ordinary_nnls	Ordinary NNLS	67	335	0.0516	0.0493	0.054	0.1195	0.1116	0.1271	0.7142	0.6977	0.731
heldout_donor_platform_shift	ridge_nnls	Ridge NNLS	67	335	0.0659	0.0638	0.0679	0.1735	0.1659	0.1811	0.6958	0.6853	0.7066
heldout_donor_platform_shift	robust_nnls_hpa	HPA-weighted robust NNLS (ReCAST)	67	335	0.0508	0.0486	0.0532	0.1169	0.1091	0.1247	0.7149	0.6986	0.7315

Scenario	Method	Method label	Donors	Mixtures	State mae mean	State mae ci95 low	State mae ci95 high	Jensen shannon mean	Jensen shannon ci95 low	Jensen shannon ci95 high	State spearman mean	State spearman ci95 low	State spearman ci95 high
heldout_donor_platform_shift	simplex_nnls	Simplex-constrained NNLS	67	335	0.0443	0.0419	0.0467	0.0952	0.0884	0.1022	0.6816	0.6672	0.696
heldout_donor_platform_shift	unbalanced_ot_hpa	HPA-weighted unbalanced OT	67	335	0.101	0.0994	0.1027	0.3265	0.3191	0.3339	0.4538	0.428	0.4785

Table S4. Projection-estimator comparators on donor-held-out mixtures.

Scenario	Comparator	Comparator label	Donors	Mean mae improvement primary	Ci95 low	Ci95 high	Wilcoxon p	Fdr bh
heldout_donor_clean	nu_svr	CIBERSORT-style nu-SVR	67	-0.0001	-0.001	0.0007	0.793	0.793
heldout_donor_clean	ordinary_nnls	Ordinary NNLS	67	0.0013	0.0011	0.0015	0.0	0.0
heldout_donor_clean	ridge_nnls	Ridge NNLS	67	0.0156	0.0148	0.0165	0.0	0.0
heldout_donor_clean	simplex_nnls	Simplex-constrained NNLS	67	-0.0063	-0.007	-0.0055	0.0	0.0
heldout_donor_clean	unbalanced_ot_hpa	HPA-weighted unbalanced OT	67	0.0502	0.0486	0.0518	0.0	0.0
heldout_donor_platform_shift	nu_svr	CIBERSORT-style nu-SVR	67	-0.002	-0.0032	-0.0008	0.0058	0.0058
heldout_donor_platform_shift	ordinary_nnls	Ordinary NNLS	67	0.0008	0.0007	0.001	0.0	0.0
heldout_donor_platform_shift	ridge_nnls	Ridge NNLS	67	0.0151	0.0143	0.0159	0.0	0.0
heldout_donor_platform_shift	simplex_nnls	Simplex-constrained NNLS	67	-0.0066	-0.0073	-0.0058	0.0	0.0
heldout_donor_platform_shift	unbalanced_ot_hpa	HPA-weighted unbalanced OT	67	0.0502	0.0487	0.0517	0.0	0.0

Table S5. External gene-level confirmation after GENCODE 50 reannotation.

Feature	Family	Log hazard ratio per sd	Hazard ratio per sd	Ci95 low	Ci95 high	P value	Penalizer	N	Events	Error	Fdr bh	Internal log hr	Internal fdr bh	Direction concordant
PTGDS	external_gene	0.2045	1.2269	0.9117	1.6512	0.1771	0.05	60	33		0.9507	-0.3051	0.9173	False
XCL1	external_gene	0.0145	1.0146	0.7579	1.3581	0.9226	0.05	60	33		0.9507	0.2733	0.9173	True
VPREB3	external_gene	0.2859	1.3309	1.0299	1.7199	0.0289	0.05	60	33		0.8702	-0.2783	0.9173	False
MMP12	external_gene	-0.1155	0.8909	0.7028	1.1295	0.34	0.05	60	33		0.9507	0.2553	0.9173	False
C5AR1	external_gene	-0.0625	0.9394	0.713	1.2379	0.6571	0.05	60	33		0.9507	0.1906	0.9173	False
CPVL	external_gene	-0.1156	0.8908	0.6646	1.194	0.4391	0.05	60	33		0.9507	0.1718	0.9173	False
IDO1	external_gene	-0.2579	0.7727	0.6052	0.9865	0.0385	0.05	60	33		0.8702	0.184	0.9173	False
MAOB	external_gene	-0.1134	0.8928	0.6899	1.1555	0.3889	0.05	60	33		0.9507	-0.1932	0.9173	True
TREM2	external_gene	-0.011	0.9891	0.7261	1.3472	0.9444	0.05	60	33		0.9507	0.1718	0.9173	False
MS4A7	external_gene	0.1444	1.1553	0.8573	1.5569	0.3428	0.05	60	33		0.9507	0.1533	0.9173	True
CD5	external_gene	-0.0372	0.9635	0.7258	1.2791	0.7971	0.05	60	33		0.9507	0.1619	0.9173	False
CALD1	external_gene	-0.038	0.9628	0.7275	1.2741	0.7906	0.05	60	33		0.9507	0.1464	0.9173	False
TNFRSF13C	external_gene	-0.0096	0.9905	0.7316	1.341	0.9507	0.05	60	33		0.9507	-0.1843	0.9173	True
BLNK	external_gene	-0.1174	0.8892	0.6274	1.2604	0.5095	0.05	60	33		0.9507	-0.1534	0.9173	True
GZMA	external_gene	0.0893	1.0934	0.8664	1.3798	0.4519	0.05	60	33		0.9507	0.1478	0.9173	True
CD96	external_gene	0.1047	1.1103	0.8702	1.4167	0.3999	0.05	60	33		0.9507	0.1496	0.9173	True
LY6D	external_gene	-0.0782	0.9248	0.7151	1.196	0.5512	0.05	60	33		0.9507	-0.2007	0.9173	True
TREM1	external_gene	-0.1315	0.8768	0.6706	1.1464	0.3366	0.05	60	33		0.9507	0.1467	0.9173	False
GATA2	external_gene	0.2348	1.2647	0.9086	1.7603	0.1639	0.05	60	33		0.9507	-0.1369	0.9173	False
CCL19	external_gene	0.0605	1.0624	0.8005	1.4101	0.6752	0.05	60	33		0.9507	-0.1409	0.9173	False

Feature	Family	Log hazard ratio per sd	Hazard ratio per sd	Ci95 low	Ci95 high	P value	Penalizer	N	Events	Error	Fdr bh	Internal log hr	Internal fdr bh	Direction concordant
PLA2G7	external_gene	-0.0611	0.9407	0.688	1.2863	0.7019	0.05	60	33		0.9507	0.1261	0.9259	False
CD247	external_gene	0.0133	1.0134	0.7875	1.304	0.9176	0.05	60	33		0.9507	0.1416	0.9259	True
PILRA	external_gene	0.0584	1.0601	0.7892	1.4241	0.6983	0.05	60	33		0.9507	0.1136	0.9259	True
PLVAP	external_gene	0.0342	1.0348	0.756	1.4164	0.831	0.05	60	33		0.9507	0.1707	0.9259	True
KRT15	external_gene	-0.2747	0.7598	0.5506	1.0485	0.0946	0.05	60	33		0.8702	-0.1598	0.9541	True
ITK	external_gene	-0.0115	0.9886	0.7455	1.311	0.9365	0.05	60	33		0.9507	0.1253	0.9541	False
HCK	external_gene	-0.1235	0.8839	0.6512	1.1995	0.4282	0.05	60	33		0.9507	0.1153	0.9541	False
SLPI	external_gene	-0.2761	0.7587	0.5572	1.0332	0.0796	0.05	60	33		0.8702	-0.1141	0.9541	True
CD6	external_gene	-0.1098	0.896	0.6809	1.1791	0.433	0.05	60	33		0.9507	0.1173	0.9541	False
CD19	external_gene	0.0778	1.0809	0.8439	1.3845	0.5377	0.05	60	33		0.9507	-0.1316	0.9541	False
MGP	external_gene	0.1834	1.2012	0.9028	1.5983	0.2082	0.05	60	33		0.9507	-0.0972	0.9541	False
DNAAF1	external_gene	-0.019	0.9812	0.7302	1.3185	0.8998	0.05	60	33		0.9507	0.1215	0.9541	False
RAB27B	external_gene	-0.2415	0.7854	0.5842	1.0561	0.1098	0.05	60	33		0.8702	-0.0873	0.9541	True
S100B	external_gene	0.0996	1.1047	0.8129	1.5014	0.5245	0.05	60	33		0.9507	0.1098	0.9541	True
KRT6B	external_gene	-0.0815	0.9217	0.701	1.2119	0.5593	0.05	60	33		0.9507	-0.1195	0.9865	True
KIT	external_gene	-0.0485	0.9527	0.6977	1.3009	0.7604	0.05	60	33		0.9507	-0.0956	0.9865	True
MMP19	external_gene	-0.0184	0.9817	0.7216	1.3357	0.9066	0.05	60	33		0.9507	0.0807	0.9865	False
NTRK1	external_gene	-0.1311	0.8772	0.6312	1.2189	0.435	0.05	60	33		0.9507	-0.0776	0.9865	True
LUM	external_gene	0.0881	1.0921	0.835	1.4283	0.52	0.05	60	33		0.9507	0.0668	0.9865	True
COL4A2	external_gene	-0.0897	0.9142	0.6176	1.3533	0.6541	0.05	60	33		0.9507	0.0818	0.9865	False
CD22	external_gene	-0.0104	0.9897	0.7429	1.3184	0.9434	0.05	60	33		0.9507	-0.0732	0.9865	True
GPX2	external_gene	-0.0779	0.925	0.6571	1.3023	0.6553	0.05	60	33		0.9507	-0.0839	0.9865	True
IL1RL1	external_gene	-0.2848	0.7522	0.5469	1.0345	0.0799	0.05	60	33		0.8702	0.0832	0.9865	False
IGFBP5	external_gene	0.0867	1.0905	0.7948	1.4962	0.5912	0.05	60	33		0.9507	0.0611	0.9865	True
CSF1R	external_gene	0.0198	1.02	0.7577	1.3732	0.896	0.05	60	33		0.9507	0.0583	0.9865	True
RARRES2	external_gene	0.0121	1.0122	0.762	1.3445	0.9334	0.05	60	33		0.9507	-0.0538	0.9865	False
GZMM	external_gene	0.3092	1.3623	1.0586	1.7532	0.0163	0.05	60	33		0.8702	0.0615	0.9865	True
JSRP1	external_gene	0.1365	1.1463	0.8381	1.5677	0.3928	0.05	60	33		0.9507	0.0599	0.9865	True
GNG11	external_gene	-0.017	0.9831	0.7113	1.3589	0.9179	0.05	60	33		0.9507	-0.0477	0.9865	True
FXYD3	external_gene	-0.102	0.903	0.6352	1.2837	0.5697	0.05	60	33		0.9507	-0.0546	0.9865	True
CTLA4	external_gene	0.0215	1.0217	0.7929	1.3164	0.8682	0.05	60	33		0.9507	0.0507	0.9865	True
KLRD1	external_gene	0.0655	1.0677	0.7807	1.4601	0.6818	0.05	60	33		0.9507	-0.0433	0.9865	False
CD8B	external_gene	0.0458	1.0468	0.7675	1.4278	0.7726	0.05	60	33		0.9507	0.0442	0.9865	True
CD24	external_gene	-0.205	0.8146	0.6309	1.052	0.116	0.05	60	33		0.8702	0.0369	0.9865	False
BTK	external_gene	-0.0748	0.9279	0.678	1.27	0.6404	0.05	60	33		0.9507	0.03	0.9865	False
S100A7	external_gene	-0.0702	0.9322	0.6928	1.2543	0.6429	0.05	60	33		0.9507	-0.0375	0.9865	True
RGS5	external_gene	0.1586	1.1719	0.8561	1.6042	0.3222	0.05	60	33		0.9507	-0.0353	0.9865	False
COL1A2	external_gene	0.0966	1.1015	0.8237	1.473	0.5146	0.05	60	33		0.9507	0.0248	0.9865	True
SLC18A2	external_gene	0.0184	1.0185	0.7431	1.3961	0.9091	0.05	60	33		0.9507	-0.0296	0.9865	False
COL15A1	external_gene	0.0792	1.0824	0.7812	1.4998	0.634	0.05	60	33		0.9507	-0.0269	0.9865	False
CTHRC1	external_gene	0.1642	1.1785	0.8993	1.5443	0.2337	0.05	60	33		0.9507	0.0206	0.9865	True
SPIB	external_gene	0.0308	1.0313	0.7489	1.4201	0.8503	0.05	60	33		0.9507	0.0234	0.9865	True
OLR1	external_gene	-0.0763	0.9265	0.6633	1.2941	0.6543	0.05	60	33		0.9507	0.0182	0.9865	False
FPR1	external_gene	-0.2278	0.7963	0.6265	1.0121	0.0626	0.05	60	33		0.8702	-0.0132	0.9865	True
BGN	external_gene	0.2467	1.2798	0.8933	1.8336	0.1787	0.05	60	33		0.9507	0.01	0.9865	True
BANK1	external_gene	0.0151	1.0152	0.7822	1.3176	0.9095	0.05	60	33		0.9507	-0.0091	0.9865	False

Feature	Family	Log hazard ratio per sd	Hazard ratio per sd	Ci95 low	Ci95 high	P value	Penalizer	N	Events	Error	Fdr bh	Internal log hr	Internal fdr bh	Direction concordant
VMO1	external_gene	-0.2147	0.8068	0.6017	1.0816	0.1512	0.05	60	33		0.9507	0.0058	0.9865	False
FCGR2B	external_gene	-0.1416	0.868	0.633	1.1901	0.3792	0.05	60	33		0.9507	0.0078	0.9865	False
RAMP2	external_gene	0.1072	1.1131	0.8287	1.4952	0.4765	0.05	60	33		0.9507	0.0079	0.9865	True
CNRIP1	external_gene	0.0093	1.0094	0.7626	1.3359	0.9481	0.05	60	33		0.9507	-0.0059	0.9865	False
TM4SF1	external_gene	0.217	1.2423	0.9734	1.5854	0.0813	0.05	60	33		0.8702	0.0066	0.9865	True
ESAM	external_gene	0.0847	1.0884	0.8243	1.4372	0.5501	0.05	60	33		0.9507	0.0077	0.9865	True
SPARCL1	external_gene	0.1836	1.2015	0.9019	1.6006	0.2096	0.05	60	33		0.9507	0.0027	0.9865	True
DCN	external_gene	0.0403	1.0411	0.807	1.343	0.7567	0.05	60	33		0.9507	0.0016	0.9865	True
CLDN4	external_gene	0.1592	1.1726	0.8852	1.5534	0.2671	0.05	60	33		0.9507	-0.0029	0.9865	False

Table S6. External pathway-level confirmation after reannotation.

Feature	Family	Log hazard ratio per sd	Hazard ratio per sd	Ci95 low	Ci95 high	P value	Penalizer	N	Events	Error	Fdr bh
pathway__REACTOME_NEUTROPHIL_DEGRANULATION	external_pathway	-0.257	0.7733	0.5531	1.0812	0.1327	0.05	60	33		0.9334
pathway__REACTOME_SARS_COV_INFECTIONS	external_pathway	0.0648	1.067	0.7683	1.4818	0.6988	0.05	60	33		0.9334
pathway__REACTOME_GPCR_LIGAND_BINDING	external_pathway	-0.1796	0.8356	0.6164	1.1329	0.2475	0.05	60	33		0.9334
pathway__REACTOME_SIGNALING_BY_INTERLEUKINS	external_pathway	-0.2128	0.8083	0.6085	1.0737	0.1418	0.05	60	33		0.9334
pathway__REACTOME_DISEASES_OF_SIGNAL_TRANSDUCTION_BY_GROWTH_FACTOR_RECEPTORS_AND_SECOND_MESSENGERS	external_pathway	-0.2036	0.8158	0.5942	1.1199	0.2079	0.05	60	33		0.9334
pathway__REACTOME_RHO_GTPASE_CYCLE	external_pathway	-0.1358	0.873	0.6364	1.1976	0.3998	0.05	60	33		0.9334
pathway__REACTOME_OLFACTORY_SIGNALING_PATHWAY	external_pathway	0.0803	1.0836	0.8153	1.4401	0.5801	0.05	60	33		0.9334
pathway__REACTOME_NEURONAL_SYSTEM	external_pathway	-0.0201	0.9801	0.7282	1.3191	0.8946	0.05	60	33		0.9334
pathway__REACTOME_M_PHASE	external_pathway	-0.0313	0.9692	0.7006	1.3408	0.85	0.05	60	33		0.9334
pathway__REACTOME_DENGUE_VIRUS_INFECTION	external_pathway	0.1106	1.117	0.8467	1.4735	0.4337	0.05	60	33		0.9334
pathway__REACTOME_CLASS_I_MHC_MEDIATED_ANTIGEN_PROCESSING_PRESENTATION	external_pathway	-0.2247	0.7988	0.5847	1.0913	0.1581	0.05	60	33		0.9334
pathway__REACTOME_TRANSLATION	external_pathway	0.0324	1.033	0.7221	1.4777	0.859	0.05	60	33		0.9334
pathway__REACTOME_TRANSCRIPTIONAL_REGULATION_BY_TP53	external_pathway	-0.1025	0.9026	0.6369	1.2791	0.5645	0.05	60	33		0.9334
pathway__REACTOME_METABOLISM_OF_AMINO_ACIDS_AND_DERIVATIVES	external_pathway	-0.1102	0.8956	0.6402	1.2529	0.5198	0.05	60	33		0.9334
pathway__REACTOME_CELL_CELL_COMMUNICATION	external_pathway	-0.0173	0.9828	0.6547	1.4755	0.9334	0.05	60	33		0.9334
pathway__REACTOME_CLASS_A_1_RHODOPSIN_LIKE_RECEPTORS	external_pathway	-0.1462	0.864	0.6334	1.1786	0.3562	0.05	60	33		0.9334
pathway__REACTOME_DNA_REPAIR	external_pathway	-0.0933	0.9109	0.6303	1.3164	0.6193	0.05	60	33		0.9334
pathway__REACTOME_EXTRACELLULAR_MATRIX_ORGANIZATION	external_pathway	0.0541	1.0556	0.7489	1.488	0.7572	0.05	60	33		0.9334
pathway__REACTOME_RHO_GTPASE_EFFECTORS	external_pathway	-0.0551	0.9464	0.6965	1.286	0.7248	0.05	60	33		0.9334
pathway__REACTOME_EPIGENETIC_REGULATION_OF_GENE_EXPRESSION	external_pathway	0.0291	1.0295	0.7458	1.4211	0.8597	0.05	60	33		0.9334
pathway__REACTOME_SIGNALING_BY_WNT	external_pathway	0.1283	1.1369	0.8247	1.5674	0.4335	0.05	60	33		0.9334

Feature	Family	Log hazard ratio per sd	Hazard ratio per sd	Ci95 low	Ci95 high	P value	Penalizer	N	Events	Error	Fdr bh
pathway__REACTOME_G_ALPHA_I_SIGNALLING_EVENTS	external_pathway	-0.0696	0.9328	0.6969	1.2486	0.6401	0.05	60	33		0.9334
pathway__REACTOME_MAPK_FAMILY_SIGNALING_CASCADES	external_pathway	-0.1091	0.8966	0.6374	1.2611	0.5306	0.05	60	33		0.9334
pathway__REACTOME_ASPARAGINE_N_LINKED_GLYCOSYLATION	external_pathway	-0.1603	0.8519	0.6357	1.1415	0.283	0.05	60	33		0.9334

Table S7. Exploratory external performance within histology subgroups.

Model	Histology	Patients	Events	Harrell c	Uno c
Elastic-net Cox	EAC	88	45	0.5249	0.55
Elastic-net Cox	ESCC	94	32	0.6657	0.6205
Random survival forest	EAC	88	45	0.5135	0.532
Random survival forest	ESCC	94	32	0.6495	0.6151
SurvivalPFN	EAC	88	45	0.523	0.5355
SurvivalPFN	ESCC	94	32	0.704	0.6462
SurvPFN (June 2026)	EAC	88	45	0.4917	0.4933
SurvPFN (June 2026)	ESCC	94	32	0.6747	0.6424
XGBoost-AFT	EAC	88	45	0.4936	0.4956
XGBoost-AFT	ESCC	94	32	0.6171	0.6395

Table S8. Histology x risk-score interaction tests.

Model	Interaction log hr	Interaction hr	Ci95 low	Ci95 high	P value	Fdr bh
Elastic-net Cox	0.3691	1.4464	0.9464	2.2105	0.0881	0.1754
Random survival forest	0.1948	1.2151	0.7792	1.8948	0.3901	0.3901
SurvivalPFN	0.3181	1.3745	0.875	2.159	0.1674	0.2093
SurvPFN (June 2026)	0.4998	1.6484	1.0643	2.5529	0.0251	0.1257
XGBoost-AFT	0.3704	1.4483	0.9252	2.267	0.1052	0.1754

Table S9. External risk-group calibration using TCGA-derived tertile cutpoints.

Model	Horizon days	Risk group	Tcga cutpoint low	Tcga cutpoint high	Patients	Events total followup	Mean predicted risk	Km observed risk	Km ci95 low	Km ci95 high
Elastic-net Cox	365	low	0.1851	0.2794	24	9	0.1638	0.1667	0.0661	0.3852
Elastic-net Cox	365	intermediate	0.1851	0.2794	15	11	0.215	0.4667	0.2562	0.7368
Elastic-net Cox	365	high	0.1851	0.2794	21	13	0.3225	0.1905	0.0761	0.4311
Elastic-net Cox	1095	low	0.5052	0.6709	25	9	0.4573	0.24	0.1157	0.458
Elastic-net Cox	1095	intermediate	0.5052	0.6709	14	11	0.5629	0.7857	0.5521	0.9479
Elastic-net Cox	1095	high	0.5052	0.6709	21	13	0.7332	0.4762	0.2912	0.7033
SurvivalPFN	365	low	0.1419	0.2858	29	12	0.1052	0.2069	0.0987	0.4036
SurvivalPFN	365	intermediate	0.1419	0.2858	11	6	0.2064	0.1818	0.0488	0.5526
SurvivalPFN	365	high	0.1419	0.2858	20	15	0.3708	0.35	0.1847	0.597
SurvivalPFN	1095	low	0.449	0.654	27	10	0.3785	0.2593	0.133	0.4681
SurvivalPFN	1095	intermediate	0.449	0.654	14	9	0.5471	0.6429	0.4056	0.8697
SurvivalPFN	1095	high	0.449	0.654	19	14	0.7266	0.5789	0.3751	0.7963
SurvPFN (June 2026)	365	low	0.196	0.3321	31	14	0.1719	0.1935	0.092	0.3809
SurvPFN (June 2026)	365	intermediate	0.196	0.3321	12	7	0.2677	0.4167	0.1991	0.7299
SurvPFN (June 2026)	365	high	0.196	0.3321	17	12	0.374	0.2353	0.0955	0.5117
SurvPFN (June 2026)	1095	low	0.4046	0.594	32	15	0.3612	0.375	0.2332	0.5648
SurvPFN (June 2026)	1095	intermediate	0.4046	0.594	11	6	0.5175	0.5455	0.2931	0.8334
SurvPFN (June 2026)	1095	high	0.4046	0.594	17	12	0.6413	0.5294	0.3203	0.7704

Table S10. PROBAST+AI author risk-of-bias self-assessment by domain.

Domain	Author judgment	Evidence	Mitigation or claim boundary
Participants and data sources	Some concerns	Retrospective public cohorts; eligibility and overlap exclusions are reproducible.	Report selection mechanisms and cohort differences; avoid deployment claims.
Predictors	Some concerns	Clinical predictors are available in both cohorts, but external gene annotation is untraceable.	Restrict external claims to clinical variables until versioned reannotation passes.
Outcome	Low	Overall survival time and death status are defined consistently and without predictor knowledge.	Report administrative differences in follow-up.
Sample size and data complexity	High	The development set has 182 patients and 77 deaths relative to multiple candidate models.	Use compact predictors, penalization, nested validation, and acknowledge imprecision.
Missing data	Low	Median imputation is fitted inside each training fold.	Publish fold-local preprocessing code and missingness counts.
Model development	Some concerns	Candidate models and tuning are prespecified, but complex learners remain unstable in small samples.	Retain elastic-net Cox and prohibit post-result model switching.
Internal evaluation	Low	Ten repeated five-fold outer splits are locked; preprocessing and tuning are training-only.	Report paired fold uncertainty and all models, including negative results.
External evaluation	Some concerns	The 60-patient test is non-overlapping but small and ESCC-only; calibration is poor.	No external recalibration or deployment claim; show confidence intervals and applicability limits.
Performance measures	Low	Uno C, Harrell C, dynamic AUC, IPCW Brier/IBS, calibration and decision curves are reported.	Interpret clinical utility as exploratory because thresholds were not prospectively chosen.
Subgroups and fairness	High	Sex, ancestry and histology strata are too small for reliable fairness conclusions.	Provide descriptive EAC/ESCC results and interaction tests; state that fairness is unresolved.
Applicability	High	Public retrospective cohorts and platform discordance limit transportability to contemporary clinical practice.	Frame as a methodological study, not a clinical decision aid.
Overall predictive-performance risk of bias	High	Strong validation safeguards do not eliminate small-sample and external-applicability limitations.	Require independent prospective or larger multicentre evaluation before clinical use.

Table S11. Follow-up maturity (a) and the 11 patients without an assignable stage (b).

Cohort	Patients	Deaths	Median observed time, d	Reverse-KM median follow-up, d	At risk 1/2/3/5 y	Deaths by 3 y	KM OS 3 y
TCGA-ESCA	182	77	396.5	608	116/41/17/4	69	0.41
TCGA-ESCA ESCC	94	32	384.5	407	65/15/5/1	29	0.43
TCGA-ESCA EAC	88	45	443.5	882	51/26/12/3	40	0.39
GSE53625 external	60	33	1197.2	1666	45/39/33/0	27	0.55

KM, Kaplan–Meier; reverse KM treats death as censoring and censoring as the event.

Patient	Histology	Tissue-source site	Death	Time, d	Recorded stage fields	pT / pN / pM
TCGA-R6-A6L4	EAC	R6	1	496	none recorded	– / – / –
TCGA-R6-A8W5	EAC	R6	1	480	none recorded	– / – / –

TCGA-R6-A6XG	EAC	R6	0	1168	none recorded	– / – / –
TCGA-R6-A8WG	EAC	R6	1	386	none recorded	– / – / –
TCGA-2H-A9GG	EAC	2H	1	610	none recorded	– / – / –
TCGA-L5-A88T	EAC	L5	0	694	none recorded	– / – / –
TCGA-R6-A6DN	EAC	R6	1	243	none recorded	– / – / –
TCGA-R6-A6Y2	EAC	R6	1	283	none recorded	– / – / –
TCGA-JY-A6FA	ESCC	JY	1	1361	none recorded	– / – / –
TCGA-L7-A56G	ESCC	L7	1	330	none recorded	– / – / –
TCGA-X8-AAAR	EAC	X8	0	554	Unknown	T1 / N1 / Unknown

Stage sources were checked in the order marker paper, GDC pathological AJCC, GDC clinical AJCC.

Table S12. BayesPrism and the ν-SVR core of CIBERSORT alongside the published comparators: (a) donor-held-out accuracy (720 mixtures, 60 donors); (b) per-state Pearson agreement for the two added methods; (c) agreement with measured composition in TCGA-ESCA overall and within each histology (Spearman ϱ, bootstrap 95% CI).

**Method**	**State MAE (95% CI)**	**Within-mixture r**
MuSiC	0.0327 (0.0307–0.0350)	0.797
DWLS	0.0329 (0.0309–0.0349)	0.795
ν-SVR (CIBERSORT core)	0.0363 (0.0347–0.0379)	0.766
BayesPrism	0.0385 (0.0363–0.0408)	0.748
ReCAST (locked)	0.0501 (0.0489–0.0512)	0.607
BisqueRNA	0.0516 (0.0505–0.0527)	0.527
Simplex NNLS (ReCAST design)	0.0538 (0.0527–0.0548)	0.601
Unbalanced OT (ReCAST design)	0.0553 (0.0538–0.0566)	0.301

Donor-clustered bootstrap intervals (2000 replicates).

**Method**	**B**	**CD4Tconv**	**CD8Tex**	**DC**	**Endothelial**	**Fibroblasts**	**Malignant**	**Mast**	**Mono/Macro**	**Pericytes**	**Plasma**	**Tprolif**	**Treg**
BayesPrism	0.68	0.32	0.68	0.91	0.94	0.87	0.88	0.95	0.95	0.93	0.89	0.89	0.71
ν-SVR	0.63	0.45	0.75	0.89	0.94	0.88	0.79	0.95	0.91	0.91	0.91	0.92	0.78

**Subset**	**Measured quantity**	**BayesPrism**	**BisqueRNA**	**DWLS**	**MuSiC**	**ReCAST**	**Simplex NNLS**	**ν-SVR**
EAC	ABSOLUTE purity	0.55 (0.35, 0.71)	0.53 (0.30, 0.71)	0.48 (0.24, 0.67)	0.42 (0.20, 0.63)	0.37 (0.14, 0.57)	0.38 (0.14, 0.59)	0.10 (-0.13, 0.32)
EAC	Estimated leukocyte fraction	0.51 (0.30, 0.70)	0.43 (0.22, 0.61)	0.37 (0.16, 0.55)	-0.18 (-0.39, 0.04)	0.35 (0.14, 0.54)	0.36 (0.15, 0.55)	-0.35 (-0.54, -0.13)
EAC	Pathology lymphocyte infiltration (%)	0.30 (0.07, 0.52)	0.05 (-0.18, 0.28)	0.17 (-0.05, 0.39)	0.02 (-0.22, 0.26)	0.24 (0.03, 0.46)	0.25 (0.04, 0.46)	0.03 (-0.18, 0.24)
EAC	Pathology tumour cells (%)	-0.20 (-0.40, 0.03)	-0.14 (-0.37, 0.09)	-0.10 (-0.33, 0.12)	-0.20 (-0.42, 0.02)	-0.17 (-0.39, 0.06)	-0.19 (-0.42, 0.05)	-0.02 (-0.25, 0.23)
EAC	Pathology tumour nuclei (%)	0.11 (-0.14, 0.34)	-0.05 (-0.29, 0.20)	0.06 (-0.17, 0.31)	0.04 (-0.20, 0.27)	-0.03 (-0.27, 0.21)	-0.01 (-0.25, 0.23)	-0.19 (-0.40, 0.04)
ESCC	ABSOLUTE purity	0.21 (-0.00, 0.43)	0.18 (-0.04, 0.39)	0.32 (0.09, 0.52)	0.18 (-0.05, 0.37)	0.05 (-0.16, 0.27)	0.08 (-0.14, 0.30)	0.07 (-0.15, 0.28)
ESCC	Estimated leukocyte fraction	0.36 (0.16, 0.53)	0.20 (-0.01, 0.40)	0.33 (0.12, 0.51)	0.14 (-0.06, 0.33)	0.30 (0.09, 0.49)	0.36 (0.15, 0.53)	-0.03 (-0.24, 0.16)
ESCC	Pathology lymphocyte infiltration (%)	0.07 (-0.13, 0.27)	0.14 (-0.08, 0.35)	-0.02 (-0.22, 0.18)	-0.00 (-0.22, 0.21)	0.09 (-0.11, 0.29)	0.09 (-0.12, 0.29)	-0.06 (-0.26, 0.15)
ESCC	Pathology tumour cells (%)	0.04 (-0.17, 0.24)	-0.07 (-0.28, 0.14)	0.01 (-0.20, 0.22)	0.03 (-0.19, 0.24)	-0.08 (-0.29, 0.13)	-0.09 (-0.30, 0.11)	-0.01 (-0.21, 0.21)
ESCC	Pathology tumour nuclei (%)	-0.02 (-0.22, 0.19)	-0.11 (-0.31, 0.11)	-0.09 (-0.29, 0.12)	-0.06 (-0.28, 0.17)	-0.06 (-0.27, 0.16)	-0.06 (-0.28, 0.16)	-0.21 (-0.40, -0.00)
all	ABSOLUTE purity	0.33 (0.18, 0.47)	0.30 (0.14, 0.45)	0.37 (0.22, 0.51)	0.21 (0.03, 0.36)	0.11 (-0.05, 0.27)	0.13 (-0.03, 0.29)	0.00 (-0.15, 0.17)

all	Estimated leukocyte fraction	0.32 (0.18, 0.46)	0.28 (0.13, 0.42)	0.26 (0.11, 0.39)	-0.00 (-0.14, 0.15)	0.22 (0.06, 0.36)	0.24 (0.08, 0.39)	-0.12 (-0.27, 0.04)
all	Pathology lymphocyte infiltration (%)	0.20 (0.05, 0.35)	0.08 (-0.09, 0.24)	0.11 (-0.03, 0.26)	0.05 (-0.12, 0.20)	0.19 (0.03, 0.33)	0.19 (0.04, 0.33)	0.08 (-0.07, 0.24)
all	Pathology tumour cells (%)	-0.11 (-0.25, 0.05)	-0.16 (-0.31, -0.01)	-0.07 (-0.22, 0.10)	-0.15 (-0.30, 0.01)	-0.21 (-0.35, -0.06)	-0.21 (-0.35, -0.06)	-0.14 (-0.28, 0.01)
all	Pathology tumour nuclei (%)	0.06 (-0.09, 0.22)	-0.06 (-0.21, 0.09)	-0.02 (-0.16, 0.13)	0.02 (-0.13, 0.18)	-0.03 (-0.19, 0.14)	-0.01 (-0.16, 0.14)	-0.10 (-0.25, 0.06)

**Design**	**Method**	**Myeloid MAE (95% CI)**	**Mixtures**
Within-study myeloid	BayesPrism	0.0910 (0.0815–0.1008)	360
Within-study myeloid	ν-SVR	0.1118 (0.1012–0.1227)	360
GSE160269 → GSE154763	BayesPrism	0.0636 (0.0484–0.0771)	280
GSE160269 → GSE154763	ν-SVR	0.1039 (0.0717–0.1367)	280
GSE154763 → GSE160269	BayesPrism	0.0893 (0.0799–0.1000)	240
GSE154763 → GSE160269	ν-SVR	0.1294 (0.1200–0.1392)	240

(d) Myeloid transfer designs.

Table S13. Reference donor-number simulation. (a) Held-out donors of the same study: anchors rebuilt from random subsets of the training donors in each of five folds and applied to the same held-out mixtures. (b) Cross-study transfer: anchors from random subsets of GSE160269 donors applied to 280 GSE154763 myeloid mixtures.

**Method**	**Donors**	**Draws**	**State MAE, mean (2.5–97.5%)**	**States modelled**
Cell-pooled anchors	2	50	0.0486 (0.0438–0.0551)	13.0
Cell-pooled anchors	3	50	0.0485 (0.0432–0.0537)	13.0
Cell-pooled anchors	4	50	0.0485 (0.0435–0.0532)	13.0
Cell-pooled anchors	6	50	0.0481 (0.0447–0.0513)	13.0
Cell-pooled anchors	8	50	0.0485 (0.0446–0.0531)	13.0
Cell-pooled anchors	12	50	0.0497 (0.0444–0.0550)	13.0
Cell-pooled anchors	16	50	0.0495 (0.0456–0.0540)	13.0
Cell-pooled anchors	24	50	0.0495 (0.0467–0.0528)	13.0
Cell-pooled anchors	36	50	0.0508 (0.0475–0.0536)	13.0
Cell-pooled anchors	48	5	0.0509 (0.0492–0.0531)	13.0
ReCAST (locked)	2	50	0.0491 (0.0439–0.0557)	13.0
ReCAST (locked)	3	50	0.0496 (0.0457–0.0551)	13.0
ReCAST (locked)	4	50	0.0495 (0.0455–0.0540)	13.0
ReCAST (locked)	6	50	0.0498 (0.0460–0.0530)	13.0
ReCAST (locked)	8	50	0.0495 (0.0465–0.0532)	13.0
ReCAST (locked)	12	50	0.0497 (0.0476–0.0524)	13.0
ReCAST (locked)	16	50	0.0496 (0.0467–0.0532)	13.0
ReCAST (locked)	24	50	0.0498 (0.0475–0.0521)	13.0
ReCAST (locked)	36	50	0.0497 (0.0482–0.0508)	13.0
ReCAST (locked)	48	5	0.0497 (0.0489–0.0502)	13.0
ReCAST-core (donor-hierarchical anchors + NNLS)	2	50	0.0489 (0.0439–0.0549)	13.0
ReCAST-core (donor-hierarchical anchors + NNLS)	3	50	0.0496 (0.0453–0.0558)	13.0
ReCAST-core (donor-hierarchical anchors + NNLS)	4	50	0.0495 (0.0458–0.0543)	13.0
ReCAST-core (donor-hierarchical anchors + NNLS)	6	50	0.0498 (0.0462–0.0532)	13.0

ReCAST-core (donor-hierarchical anchors + NNLS)	8	50	0.0498 (0.0465–0.0532)	13.0
ReCAST-core (donor-hierarchical anchors + NNLS)	12	50	0.0500 (0.0472–0.0527)	13.0
ReCAST-core (donor-hierarchical anchors + NNLS)	16	50	0.0501 (0.0468–0.0533)	13.0
ReCAST-core (donor-hierarchical anchors + NNLS)	24	50	0.0503 (0.0480–0.0527)	13.0
ReCAST-core (donor-hierarchical anchors + NNLS)	36	50	0.0504 (0.0486–0.0519)	13.0
ReCAST-core (donor-hierarchical anchors + NNLS)	48	5	0.0506 (0.0498–0.0512)	13.0

ReCAST-core keeps donor-hierarchical anchors and omits protein weighting and Huber reweighting.

**Method**	**Donors**	**Draws**	**Myeloid MAE, mean (2.5–97.5%)**	**Cell-pooled minus locked**	**Draws favouring locked**
Cell-pooled anchors	2	10	0.1481 (0.1368–0.1730)	0.0006	0.60
Cell-pooled anchors	3	10	0.1496 (0.1400–0.1557)	0.0026	0.60
Cell-pooled anchors	4	10	0.1455 (0.1413–0.1505)	0.0046	0.70
Cell-pooled anchors	6	10	0.1446 (0.1422–0.1500)	0.0067	1.00
Cell-pooled anchors	8	10	0.1454 (0.1388–0.1521)	0.0067	0.90
Cell-pooled anchors	12	10	0.1453 (0.1433–0.1470)	0.0124	1.00
Cell-pooled anchors	16	10	0.1444 (0.1394–0.1520)	0.0116	1.00
Cell-pooled anchors	24	10	0.1447 (0.1419–0.1488)	0.0136	1.00
Cell-pooled anchors	36	10	0.1440 (0.1413–0.1460)	0.0128	1.00
Cell-pooled anchors	48	10	0.1444 (0.1427–0.1458)	0.0142	1.00
Cell-pooled anchors	60	1	0.1450 (0.1450–0.1450)	0.0139	1.00
ReCAST (locked)	2	10	0.1476 (0.1416–0.1668)	0.0006	0.60
ReCAST (locked)	3	10	0.1470 (0.1341–0.1757)	0.0026	0.60
ReCAST (locked)	4	10	0.1410 (0.1341–0.1522)	0.0046	0.70
ReCAST (locked)	6	10	0.1379 (0.1344–0.1412)	0.0067	1.00
ReCAST (locked)	8	10	0.1387 (0.1330–0.1471)	0.0067	0.90
ReCAST (locked)	12	10	0.1329 (0.1294–0.1370)	0.0124	1.00
ReCAST (locked)	16	10	0.1328 (0.1303–0.1370)	0.0116	1.00
ReCAST (locked)	24	10	0.1311 (0.1288–0.1334)	0.0136	1.00
ReCAST (locked)	36	10	0.1313 (0.1281–0.1333)	0.0128	1.00
ReCAST (locked)	48	10	0.1302 (0.1287–0.1323)	0.0142	1.00
ReCAST (locked)	60	1	0.1311 (0.1311–0.1311)	0.0139	1.00
ReCAST-core (donor-hierarchical anchors + NNLS)	2	10	0.1492 (0.1440–0.1658)	0.0006	0.60
ReCAST-core (donor-hierarchical anchors + NNLS)	3	10	0.1485 (0.1365–0.1743)	0.0026	0.60
ReCAST-core (donor-hierarchical anchors + NNLS)	4	10	0.1428 (0.1365–0.1533)	0.0046	0.70
ReCAST-core (donor-hierarchical anchors + NNLS)	6	10	0.1397 (0.1368–0.1433)	0.0067	1.00
ReCAST-core (donor-hierarchical anchors + NNLS)	8	10	0.1401 (0.1344–0.1471)	0.0067	0.90
ReCAST-core (donor-hierarchical anchors + NNLS)	12	10	0.1344 (0.1307–0.1393)	0.0124	1.00

ReCAST-core (donor-hierarchical anchors + NNLS)	16	10	0.1341 (0.1315–0.1384)	0.0116	1.00
ReCAST-core (donor-hierarchical anchors + NNLS)	24	10	0.1325 (0.1302–0.1355)	0.0136	1.00
ReCAST-core (donor-hierarchical anchors + NNLS)	36	10	0.1326 (0.1302–0.1348)	0.0128	1.00
ReCAST-core (donor-hierarchical anchors + NNLS)	48	10	0.1315 (0.1299–0.1335)	0.0142	1.00
ReCAST-core (donor-hierarchical anchors + NNLS)	60	1	0.1325 (0.1325–0.1325)	0.0139	1.00

Paired columns compare cell-pooled with donor-hierarchical (locked) anchors on identical donor subsets.

Table S14. Transfer to adenocarcinoma. (a) Agreement of each EAC patient's state profile (GSE222078) with the locked ESCC-derived anchors, against held-out ESCC donors; (b) accuracy on 240 EAC pseudo-bulk mixtures; (c) agreement with measured composition in TCGA-ESCA by histology for the locked, histology-matched and pooled references; (d) EAC–ESCC differences (rank-biserial, + = higher in EAC) under each reference.

**State**	**EAC patients**	**EAC ρ to ESCC anchor (median)**	**EAC matching anchor first**	**Held-out ESCC ρ (median)**	**Held-out ESCC matching anchor first**
B	8	0.87	1.00	0.85	1.00
CD4Tconv	8	0.89	1.00	0.78	0.60
CD8Tex	8	0.89	0.75	0.82	0.82
DC	8	0.85	1.00	0.78	1.00
Endothelial	5	0.89	1.00	0.87	1.00
Fibroblasts	8	0.79	0.00	0.85	1.00
Malignant	5	0.71	1.00	0.88	1.00
Mast	8	0.84	1.00	0.78	1.00
Mono/Macro	8	0.87	1.00	0.85	1.00
Pericytes	3	0.76	1.00	0.84	1.00
Plasma	8	0.87	1.00	0.77	1.00
Tprolif	7	0.88	0.86	0.86	1.00
Treg	7	0.91	0.86	0.80	0.92

**Method**	**Lineage MAE (95% CI)**	**State MAE (95% CI)**	**Malignant**	**Lymphoid**	**Myeloid**	**Stromal**
DWLS (ESCC cell reference)	0.0585 (0.0486–0.0681)	0.0578 (0.0526–0.0621)	0.051	0.048	0.041	0.094
BayesPrism (ESCC cell reference)	0.0598 (0.0485–0.0715)	0.0574 (0.0543–0.0609)	0.049	0.085	0.039	0.066
MuSiC (ESCC cell reference)	0.0655 (0.0598–0.0712)	0.0790 (0.0764–0.0813)	0.043	0.105	0.069	0.045
v-SVR (ESCC cell reference)	0.0742 (0.0653–0.0825)	0.0769 (0.0708–0.0827)	0.033	0.105	0.100	0.060
BisqueRNA (ESCC cell reference)	0.0991 (0.0881–0.1107)	0.0572 (0.0536–0.0606)	0.064	0.122	0.092	0.119
ESCC-derived anchors (locked reference)	0.1022 (0.0922–0.1105)	0.0568 (0.0535–0.0599)	0.031	0.159	0.051	0.168
ESCC + EAC anchors (leave-one-donor-out)	0.1035 (0.0941–0.1116)	0.0571 (0.0541–0.0597)	0.030	0.164	0.051	0.169
EAC-derived anchors (leave-one-donor-out)	0.1159 (0.1076–0.1250)	0.0545 (0.0509–0.0574)	0.034	0.217	0.068	0.145

Patient-clustered bootstrap intervals; lineages are malignant, lymphoid, myeloid and stromal.

Histology	Measure	Histology-matched (EAC malignant anchor in EAC)	Locked (ESCC-derived)	Pooled ESCC + EAC reference
EAC	ABSOLUTE purity	0.32 (0.10, 0.52)	0.37 (0.14, 0.56)	0.38 (0.14, 0.58)
EAC	Estimated leukocyte fraction	0.23 (0.00, 0.43)	0.35 (0.12, 0.53)	0.37 (0.15, 0.56)
EAC	Pathology lymphocyte infiltration (%)	0.25 (0.03, 0.45)	0.24 (0.03, 0.45)	0.25 (0.02, 0.45)
EAC	reconstruction cosine (mean)	0.900	0.901	0.903
EAC	unknown-expression score (mean)	0.436	0.433	0.428
ESCC	ABSOLUTE purity	0.05 (-0.17, 0.26)	0.05 (-0.17, 0.26)	0.06 (-0.17, 0.27)
ESCC	Estimated leukocyte fraction	0.30 (0.09, 0.49)	0.30 (0.10, 0.49)	0.35 (0.15, 0.52)
ESCC	Pathology lymphocyte infiltration (%)	0.09 (-0.11, 0.28)	0.09 (-0.12, 0.28)	0.10 (-0.10, 0.30)
ESCC	reconstruction cosine (mean)	0.919	0.919	0.920
ESCC	unknown-expression score (mean)	0.393	0.393	0.391

State	Histology-matched (EAC malignant anchor in EAC)	Locked (ESCC-derived)	Pooled ESCC + EAC reference
B	0.65 (FDR 3.5e-13)	0.48 (FDR 7.2e-08)	0.47 (FDR 1.3e-07)
DC	-0.22 (FDR 1.2e-04)	0.43 (FDR 1.5e-07)	0.39 (FDR 1.8e-06)
Endothelial	0.84 (FDR 3.0e-21)	0.77 (FDR 5.7e-18)	0.74 (FDR 9.9e-17)
Fibroblasts	-0.07 (FDR 5.8e-01)	-0.28 (FDR 2.7e-03)	-0.32 (FDR 3.3e-04)
Malignant	-0.54 (FDR 1.3e-09)	-0.67 (FDR 4.9e-14)	-0.65 (FDR 2.9e-13)
Mast	0.05 (FDR 6.7e-01)	0.61 (FDR 4.4e-12)	0.62 (FDR 2.8e-12)

Table S15. Cell states, driver alterations and molecular subtypes in TCGA-ESCA (exploratory, descriptive). (a) Altered patients by histology; (b) EAC–ESCC differences in rank-transformed state fractions before and after adjustment for all 16 alterations; (c) the 20 smallest p-values among the 208 state-by-alteration tests (none with FDR < 0.10); (d) ESCC molecular subtypes C1 and C2 (Kruskal–Wallis).

Alteration	EAC	ESCC
ARID1A mutation	10	3
CDKN2A mutation	13	1
CDKN2A silencing	40	3
EP300 mutation	4	5
ERBB2 amplification	25	3
ERBB2 mutation	11	0
FBXW7 mutation	8	2
KMT2D mutation	5	16
NFE2L2 mutation	1	17
NOTCH1 mutation	1	13
PIK3CA mutation	5	11
SMAD4 mutation	9	3
SMARCA4 mutation	5	7
TGFBR2 mutation	2	8
TP53 mutation	70	87
ZNF750 mutation	1	11

Mutations are non-synonymous MC3 calls (cBioPortal); ERBB2 amplification and CDKN2A silencing from the TCGA marker paper.

State	Histology effect, unadjusted	Histology effect, adjusted for 16 alterations	Adjusted p	Adjusted FDR
B	0.240	0.238	4.1e-05	1.1e-04
CD4Tconv	0.033	0.047	4.2e-01	5.0e-01
CD8Tex	0.001	-0.010	5.3e-01	5.7e-01
DC	0.216	0.251	9.3e-06	3.0e-05
Endothelial	0.383	0.401	2.0e-14	2.6e-13
Fibroblasts	-0.138	-0.078	2.2e-01	3.6e-01

Malignant	-0.334	-0.303	4.5e-08	2.0e-07
Mast	0.306	0.324	2.5e-08	1.6e-07
Mono/Macro	-0.114	-0.158	1.3e-02	2.9e-02
Pericytes	0.062	0.058	3.6e-01	5.0e-01
Plasma	0.131	0.081	2.0e-01	3.6e-01
Tprolif	0.025	-0.024	7.1e-01	7.1e-01
Treg	-0.095	-0.044	3.9e-01	5.0e-01

**State**	**Alteration**	**Altered, n**	**Rank effect (adjusted for histology)**	**p**	**FDR**
Pericytes	KMT2D mutation	21	-0.178	0.008	0.74
Tprolif	ERBB2 mutation	11	0.236	0.011	0.74
Endothelial	PIK3CA mutation	16	-0.137	0.016	0.74
Endothelial	SMAD4 mutation	12	-0.154	0.018	0.74
B	NFE2L2 mutation	18	-0.159	0.019	0.74
CD8Tex	TP53 mutation	157	-0.038	0.021	0.74
DC	CDKN2A mutation	14	-0.146	0.038	0.87
B	KMT2D mutation	21	-0.123	0.047	0.87
B	SMAD4 mutation	12	-0.156	0.050	0.87
CD4Tconv	ARID1A mutation	13	-0.145	0.062	0.87
Pericytes	NOTCH1 mutation	14	0.152	0.066	0.87
B	TGFBR2 mutation	10	-0.158	0.067	0.87
CD4Tconv	ERBB2 mutation	11	-0.156	0.070	0.87
Malignant	KMT2D mutation	21	0.100	0.070	0.87
Mast	NFE2L2 mutation	18	0.114	0.074	0.87
B	CDKN2A silencing	43	-0.096	0.075	0.87
CD4Tconv	KMT2D mutation	21	0.111	0.077	0.87
Plasma	ERBB2 mutation	11	0.160	0.080	0.87
CD8Tex	FBXW7 mutation	10	0.043	0.080	0.87
Endothelial	ERBB2 mutation	11	-0.120	0.087	0.90

**Subtype system**	**Groups**	**State**	**p**	**FDR**
ESCC TCGA subtype (ESCC1/2/3)	C1=50;C2=36	B	0.531	0.668
ESCC TCGA subtype (ESCC1/2/3)	C1=50;C2=36	CD4Tconv	0.005	0.034
ESCC TCGA subtype (ESCC1/2/3)	C1=50;C2=36	CD8Tex	0.396	0.572
ESCC TCGA subtype (ESCC1/2/3)	C1=50;C2=36	DC	0.250	0.406
ESCC TCGA subtype (ESCC1/2/3)	C1=50;C2=36	Endothelial	0.047	0.087
ESCC TCGA subtype (ESCC1/2/3)	C1=50;C2=36	Fibroblasts	0.016	0.053
ESCC TCGA subtype (ESCC1/2/3)	C1=50;C2=36	Malignant	0.027	0.059
ESCC TCGA subtype (ESCC1/2/3)	C1=50;C2=36	Mast	0.005	0.034
ESCC TCGA subtype (ESCC1/2/3)	C1=50;C2=36	Mono/Macro	0.011	0.046
ESCC TCGA subtype (ESCC1/2/3)	C1=50;C2=36	Pericytes	0.026	0.059
ESCC TCGA subtype (ESCC1/2/3)	C1=50;C2=36	Plasma	0.834	0.834
ESCC TCGA subtype (ESCC1/2/3)	C1=50;C2=36	Tprolif	0.618	0.669
ESCC TCGA subtype (ESCC1/2/3)	C1=50;C2=36	Treg	0.565	0.668

EAC subtypes were not compared: 78 tumours were CIN, 2 MSI and none GS.

Table S16. Sensitivity analyses of the incremental prognostic value of ReCAST features. (a) Locked out-of-fold predictions evaluated with truncated horizons and within subgroups; (b) elastic-net Cox refitted under the locked nested 10 × 5-fold scheme within each cohort, and with features from the histology-matched reference.

Model	Analysis	Clinical Uno C	Clinical + ReCAST	ΔUno C (95% CI)	Repetitions favouring clinical
elastic_net_cox	EAC subgroup	0.554	0.513	-0.040 (-0.160 to 0.079)	7
random_survival_forest	EAC subgroup	0.558	0.455	-0.103 (-0.331 to 0.126)	9
survivalpfn	EAC subgroup	0.543	0.493	-0.049 (-0.134 to 0.035)	8
xgb_aft	EAC subgroup	0.485	0.488	0.003 (-0.151 to 0.156)	6
elastic_net_cox	ESCC subgroup	0.674	0.625	-0.049 (-0.135 to 0.037)	10
random_survival_forest	ESCC subgroup	0.633	0.510	-0.124 (-0.282 to 0.035)	10
survivalpfn	ESCC subgroup	0.685	0.658	-0.027 (-0.130 to 0.075)	9
xgb_aft	ESCC subgroup	0.641	0.493	-0.148 (-0.295 to -0.001)	10
elastic_net_cox	Truncated at 1 y	0.587	0.538	-0.049 (-0.105 to 0.008)	10
random_survival_forest	Truncated at 1 y	0.576	0.485	-0.091 (-0.202 to 0.019)	10
survivalpfn	Truncated at 1 y	0.609	0.561	-0.048 (-0.107 to 0.010)	10
xgb_aft	Truncated at 1 y	0.582	0.511	-0.071 (-0.187 to 0.045)	10
elastic_net_cox	Truncated at 2 y	0.601	0.565	-0.036 (-0.083 to 0.011)	10
random_survival_forest	Truncated at 2 y	0.570	0.495	-0.075 (-0.165 to 0.014)	10
survivalpfn	Truncated at 2 y	0.607	0.578	-0.029 (-0.074 to 0.016)	9
xgb_aft	Truncated at 2 y	0.557	0.516	-0.041 (-0.130 to 0.048)	10
elastic_net_cox	Truncated at 3 y	0.600	0.563	-0.037 (-0.081 to 0.006)	10
random_survival_forest	Truncated at 3 y	0.571	0.499	-0.072 (-0.155 to 0.012)	10
survivalpfn	Truncated at 3 y	0.608	0.579	-0.029 (-0.075 to 0.017)	9
xgb_aft	Truncated at 3 y	0.550	0.513	-0.037 (-0.130 to 0.057)	10
elastic_net_cox	All patients	0.600	0.555	-0.045 (-0.135 to 0.044)	10
random_survival_forest	All patients	0.592	0.486	-0.106 (-0.255 to 0.044)	10
survivalpfn	All patients	0.593	0.560	-0.033 (-0.089 to 0.023)	10
xgb_aft	All patients	0.544	0.494	-0.049 (-0.155 to 0.057)	9
elastic_net_cox	Recorded stage only	0.611	0.560	-0.051 (-0.147 to 0.045)	10
random_survival_forest	Recorded stage only	0.602	0.474	-0.129 (-0.281 to 0.024)	10
survivalpfn	Recorded stage only	0.598	0.566	-0.032 (-0.093 to 0.028)	9
xgb_aft	Recorded stage only	0.553	0.493	-0.060 (-0.175 to 0.055)	9

Intervals are corrected repeated cross-validation t intervals (50 outer folds).

Cohort	Patients	Deaths	Metric	Clinical	Clinical + ReCAST	Improvement (95% CI)
all_patients_refit	182	77	uno_c	0.600	0.555	-0.045 (-0.135 to 0.044)
all_patients_refit	182	77	harrell_c	0.599	0.557	-0.042 (-0.088 to 0.004)
all_patients_refit	182	77	integrated_brier	0.227	0.238	-0.011 (-0.028 to 0.006)
ESCC_only	94	32	uno_c	0.642	0.617	-0.025 (-0.110 to 0.060)
ESCC_only	94	32	harrell_c	0.688	0.636	-0.053 (-0.139 to 0.034)
ESCC_only	94	32	integrated_brier	0.214	0.243	-0.029 (-0.094 to 0.037)
EAC_only	88	45	uno_c	0.514	0.456	-0.058 (-0.151 to 0.035)
EAC_only	88	45	harrell_c	0.490	0.432	-0.058 (-0.141 to 0.026)
EAC_only	88	45	integrated_brier	0.228	0.249	-0.021 (-0.054 to 0.013)
recorded_stage_only	171	69	uno_c	0.587	0.552	-0.035 (-0.105 to 0.035)
recorded_stage_only	171	69	harrell_c	0.611	0.575	-0.036 (-0.080 to 0.009)
recorded_stage_only	171	69	integrated_brier	0.234	0.240	-0.006 (-0.029 to 0.016)

all patients, histology-matched features	182	77	uno_c	0.600	0.559	-0.041 (-0.135 to 0.054)
all patients, histology-matched features	182	77	harrell_c	0.599	0.569	-0.030 (-0.076 to 0.015)
all patients, histology-matched features	182	77	integrated_brier	0.227	0.235	-0.008 (-0.040 to 0.025)
EAC only, histology-matched features	88	45	uno_c	0.514	0.521	0.007 (-0.098 to 0.112)
EAC only, histology-matched features	88	45	harrell_c	0.490	0.508	0.018 (-0.082 to 0.117)
EAC only, histology-matched features	88	45	integrated_brier	0.228	0.231	-0.003 (-0.059 to 0.053)

Improvement is candidate minus clinical for C-indices and clinical minus candidate for the integrated Brier score.
